# Sterically controlled reductive oligomerisations of CO by activated magnesium(i) compounds: deltate *vs.* ethenediolate formation†

**DOI:** 10.1039/d0sc00836b

**Published:** 2020-03-06

**Authors:** K. Yuvaraj, Iskander Douair, Dafydd D. L. Jones, Laurent Maron, Cameron Jones

**Affiliations:** School of Chemistry, Monash University PO Box 23 VIC 3800 Australia cameron.jones@monash.edu http://www.monash.edu/science/research-groups/chemistry/jonesgroup; Université de Toulouse et CNRS, INSA, UPS UMR 5215, LPCNO, 135 Avenue de Rangueil F-31077 Toulouse France laurent.maron@irsamc.ups-tlse.fr

## Abstract

An extremely bulky, symmetrical three-coordinate magnesium(i) complex, [{(^TCHP^Nacnac)Mg}_2_] (^TCHP^Nacnac = [{(TCHP)NCMe}_2_CH]^−^, TCHP = 2,4,6-tricyclohexylphenyl) has been prepared and shown to have an extremely long Mg–Mg bond (3.021(1) Å) for such a complex. It was shown not to react with either DMAP (4-dimethylaminopyridine) or CO. Three unsymmetrical 1 : 1 DMAP adducts of less bulky Mg–Mg bonded species have been prepared, *viz.* [(^Ar^Nacnac)Mg–Mg(DMAP)(^Ar^Nacnac)] (^Ar^Nacnac = [(ArNCMe)_2_CH]^−^ Ar = 2,6-xylyl (Xyl), mesityl (Mes) or 2,6-diethylphenyl (Dep)), and their reactivity toward CO explored. Like the previously reported bulkier complex, [(^Dip^Nacnac)Mg–Mg(DMAP)(^Dip^Nacnac)] (Dip = 2,6-diisopropylphenyl), [(^Dep^Nacnac)Mg–Mg(DMAP)(^Dep^Nacnac)] reductively trimerises CO to give a rare example of a deltate complex, [{(^Dep^Nacnac)Mg(μ-C_3_O_3_)Mg(DMAP)(^Dep^Nacnac)}_2_]. In contrast, the two smaller adduct complexes react with only two CO molecules, ultimately giving unusual ethenediolate complexes [{(^Ar^Nacnac)Mg{μ-OC(H)=C(DMAP^−H^)O}Mg(^Ar^Nacnac)}_2_] (Ar = Xyl or Mes). DFT calculations show the latter reactions to proceed *via* reductive dimerizations of CO, and subsequent intramolecular C–H activation of Mg-ligated DMAP by “zig–zag” [C_2_O_2_]^2−^ fragments of reaction intermediates. Calculations also suggest that magnesium deltate complexes are kinetic products in these reactions, while the magnesium ethenediolates are thermodynamic products. This study shows that subtle changes to the bulk of the reacting 1 : 1 DMAP–magnesium(i) adduct complexes can lead to fine steric control over the products arising from their CO reductive oligomerisations. Furthermore, it is found that the more activated nature of the adduct complexes, relative to their symmetrical, three-coordinate counterparts, [{(^Ar^Nacnac)Mg}_2_], likely derives more from the polarisation of the Mg–Mg bonds of the former, than the elongated nature of those bonds.

## Abstract 2

Subtle changes to the bulk of 1 : 1 adducts of DMAP with magnesium(i) complexes leads to steric control over the products arising from their reductive oligomerisations of carbon monoxide.

## Graphical abstract

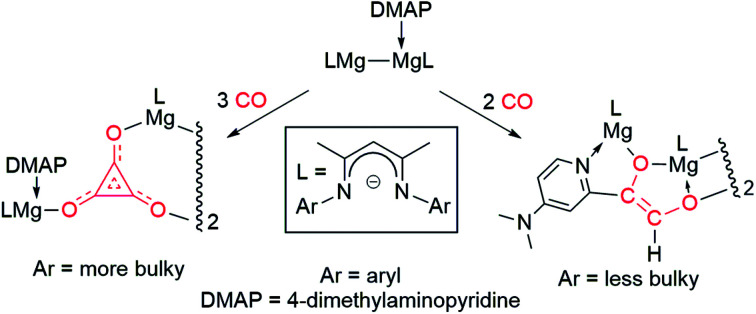

## Introduction

Carbon monoxide is a readily available C_1_ feedstock gas, that is used in many industrial processes for the production of hundreds of millions of tonnes of commodity chemicals each year. One of the most important of these processes is Fischer–Tropsch (F–T), which typically utilises heterogeneous transition metal catalysts to transform synthesis gas (CO/H_2_) into liquid hydrocarbons and oxygenates on a massive scale.^1^ Considering the importance of F–T, a great deal of effort has focussed on investigating the mechanisms by which it operates. These investigations have increasingly involved the use of low-valent organometallic complexes as soluble models to shed light on the fundamental steps, *e.g.* C–C bond formations, that are central to the process.^2^ Such studies have the potential to enhance the selectivity and energy efficiency of F–T, and to aid the development of new homogeneous catalysts of commercial importance.

In the realm of homogeneous organometallic models for the F–T process, considerable recent interest has been directed towards the activation and reductive homologation of CO, a normally inert gas which possesses one of the strongest bonds known (BDE = 257 kcal mol^−1^ (ref. 3)). For example, this work has led to the discovery that low-valent metal and non-metal compounds, from across the periodic table, can reductively oligomerise CO to ethynediolate, [OC≡CO]^2−^, aromatic oxocarbon dianions, [C_*n*_O_*n*_]^2−^ (*n* = 2–6), and related species, under mild conditions.^4–6^ From a historical perspective, it should be noted that alkali metals have been known to reductively oligomerise CO to salts of oxocarbon dianions since the early 19th century, though those salts have been poorly characterised.^7^

**Scheme 1:**
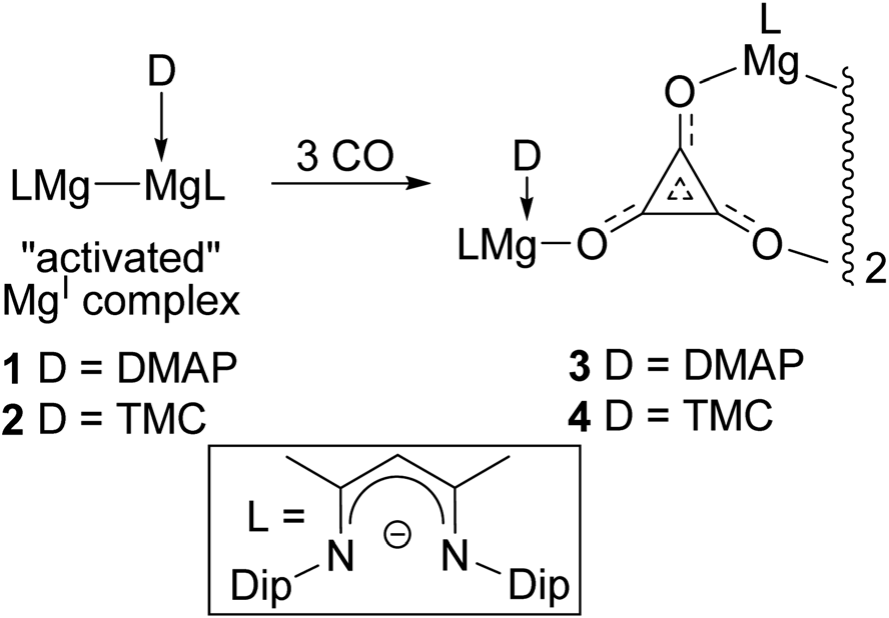
Previously reported syntheses of magnesium deltate complexes, **3** and **4** (Dip = 2,6-diisopropylphenyl, DMAP = 4-dimethylaminopyridine, TMC = :C{(MeNCMe)_2_}).^8^

In order to access well-defined s-block metal complexes of oxocarbon anions derived from CO, we have recently reported on reactions of this gas with reducing β-diketiminate coordinated magnesium(i) compounds.^8,9^ Initially, it was found that three-coordinate examples of these Mg–Mg bonded species, *viz.* [{(^Ar^Nacnac)Mg}_2_] ^Ar^Nacnac = [(ArNCMe)_2_CH]^−^; (Ar = xylyl (Xyl), mesityl (Mes), 2,6-diethylphenyl (Dep) or 2,6-diisopropylphenyl (Dip)), were largely unreactive towards CO under ambient conditions.^10^ However, addition of sub-stoichiometric amounts of Lewis bases (4-dimethylaminopyridine (DMAP) or N-heterocyclic carbenes (NHCs)) to the compounds led to the formation of 1 : 1 adduct complexes, [(^Ar^Nacnac)Mg–Mg(L)(^Ar^Nacnac)] (L = DMAP or NHC), which have substantially elongated Mg–Mg bonds (*ca.* 2.94–3.09 Å). The consequential activation of these magnesium(i) systems was borne out by the fact that two ^Dip^Nacnac substituted examples, **1** and **2**, were shown to selectively reductively trimerise CO to give the deltate complexes **3** and **4** under ambient conditions (Scheme 1).^8^ The only prior example of a structurally authenticated deltate complex was reported by Cloke and co-workers to arise from reductive trimerisation of CO by an organo-uranium(iii) complex.^6*a*^ In light of our preparations of **3** and **4**, we were interested in investigating the effect that the steric profile of a magnesium(i) reductant has on the outcome of its reaction with CO. Here, we show that magnesium deltate or ethenediolate complexes can be selectively prepared, simply by altering the β-diketiminate *N*-aryl substituent. Computational studies have been used to probe the mechanisms of the observed reactions, and the origins of reaction product selectivity.

## Results and discussion

At the outset, a magnesium(i) adduct complex bearing substantially bulkier β-diketiminate ligands than the ^Dip^Nacnac substituents in **1** and **2**, was targeted. The reasoning here stemmed from recent work by Harder and co-workers, who showed that the extremely bulky magnesium(i) compound, [(^DiPep^Nacnac)Mg–Mg(^DiPep^Nacnac)] (DiPep = 2,6-diisopentylphenyl), has an Mg–Mg bond (3.0513(8) Å) that is *ca.* 0.2 Å longer than in any other symmetrical three-coordinate complex, and similar in length to those in the activated adduct complexes, **1** and **2**.^11^ If a 1 : 1 adduct of such a bulky magnesium(i) compound could be formed, its Mg–Mg bond would likely be even longer, and more activated.

**Fig. 1:**
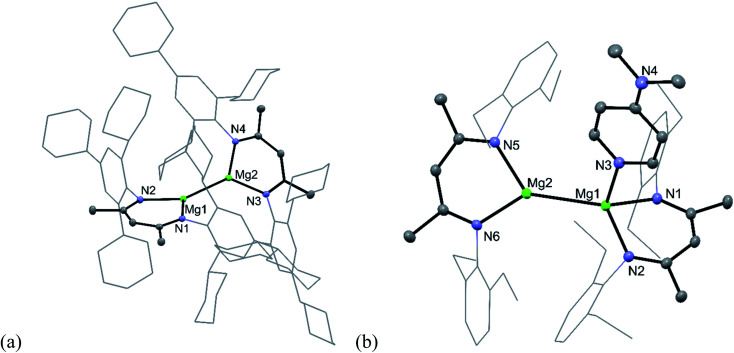
Molecular structures of (a) **5** and (b) **8** (25% thermal ellipsoids are shown; hydrogen atoms omitted; aryl substituents shown as wireframe for clarity). Selected bond lengths (Å) and angles (°) for **5**: Mg(1)–Mg(2) 3.0208(9), N(1)–Mg(1)–N(2) 91.00(7), N(4)–Mg(2)–N(3) 90.86(7). Selected bond lengths (Å) and angles (°) for **8**: Mg(1)–N(1) 2.1037(14), Mg(1)–N(2) 2.1084(13), Mg(1)–N(3) 2.1790(14), Mg(1)–Mg(2) 2.9336(7), Mg(2)–N(6) 2.0894(13), Mg(2)–N(5) 2.1004(13), N(1)–Mg(1)–N(2) 89.18(5), N(1)–Mg(1)–N(3) 96.38(5), N(2)–Mg(1)–N(3) 101.97(5), N(6)–Mg(2)–N(5) 89.20(5).

To test this hypothesis the very hindered magnesium(i) complex [{(^TCHP^Nacnac)Mg}_2_] (^TCHP^Nacnac = [{(TCHP)NCMe}_2_CH]^−^, TCHP = 2,4,6-tricyclohexylphenyl) **5** was prepared in good isolated yield, by reduction of a toluene/diethyl ether solution of [(^TCHP^Nacnac)MgI(OEt_2_)] over a sodium mirror (see ESI† for full details).^12^ However, subsequent treatment of **5** with one equivalent of either DMAP or the NHC, :C{(MeNCMe)_2_}, led to no reaction, presumably due to steric inaccessibility of its Mg centres, as is evident from its molecular structure (Fig. 1a). This is in contrast to Harder's similarly bulky magnesium(i) dimer, which did show evidence of adduct formation when treated with DMAP.^11^ Despite compound **5** possessing an Mg–Mg bond similar in length (3.021(1) Å) to that in Harder's compound, and to those in activated **1** and **2**, it proved unreactive towards CO, even when the mixture was heated at 60 °C for hours. Considering the size of the CO molecule, it seems unlikely that this lack of reactivity derives solely from the steric bulk of **5**, and perhaps indicates that unsymmetrical adduct complexes, [(^Ar^Nacnac)Mg–Mg(L)(^Ar^Nacnac)], are required to enable CO reduction (see below).

So as to explore this in more detail, attention turned to the preparation of magnesium(i) compounds, related to **1** and **2**, but in this case, bearing smaller β-diketiminate ligands. To this end the magnesium(i) compounds, [{(^Ar^Nacnac)Mg}_2_] (Ar = Xyl, Mes or Dep),^10^ were all treated with one equivalent of DMAP, in two cases affording the 1 : 1 adduct complexes, [(^Ar^Nacnac)Mg–Mg(DMAP)(^Ar^Nacnac)] (Ar = Xyl **6**, or Dep **8**), in moderate to good isolated yields as red or red-orange crystalline solids.^13^ While the mesityl substituted analogue of these compounds, [(^Mes^Nacnac)Mg–Mg(DMAP)(^Mes^Nacnac)] **7**, could not be isolated, it could be generated *in situ* and used for further reactions. Compounds **6** and **8** are oxygen sensitive, but stable in the solid state and solution for days at room temperature. Similar to the situation for solutions of **1** and **2**,^8^ variable temperature NMR spectroscopic studies revealed fluxional behaviour for **6** and **8**, which is believed to originate from rapid “hopping” of the Lewis base donor between the two Mg centres of the adduct complexes at room temperature (see ESI† for further discussion).

The solid-state molecular structures of the adducts (see Fig. 1b for the molecular structure of **8**) are also reminiscent of those for **1** and **2**, and show both to possess one trigonal planar, and one distorted tetrahedral, magnesium centre. Interestingly, their Mg–Mg bond lengths (**6**: 2.8925(9) Å; **8**: 2.9336(7) Å), while longer than those typically seen for symmetrical, three-coordinate magnesium(i) compounds (*e.g.* 2.875(1) Å for [{(^Dep^Nacnac)Mg}_2_]^10^), are not as long as the metal–metal interaction in **1** (*viz.* 3.0886(6) Å). Indeed, the Mg–Mg distances in this trio of compounds are loosely proportional to the size of their β-diketiminate ligands, and cover a range of more than 0.2 Å. This observation is fully consistent with the previously computed shallow potential energy surface for the elongation of Mg–Mg bonds in compounds such as [{(^Ar^Nacnac)Mg}_2_].^13*a*^

**Scheme 2:**
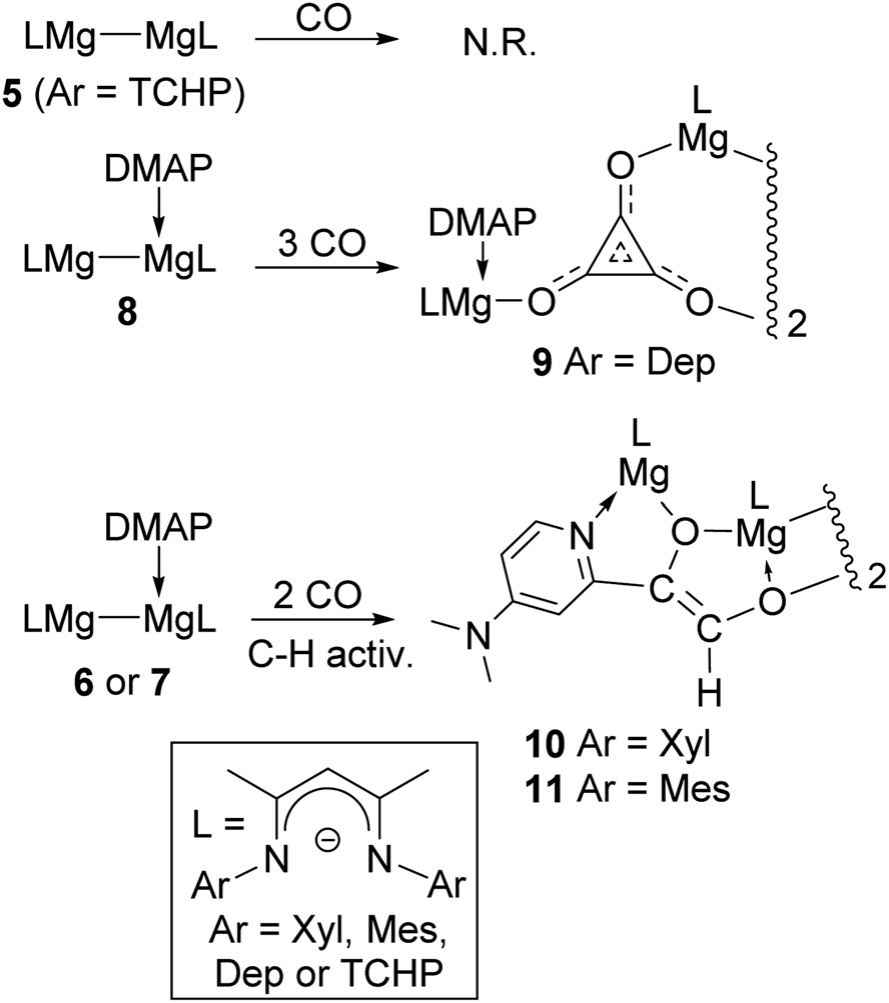
Syntheses of compounds **9–11**.

With **6** and **8** in hand, toluene solutions of the compounds, and of *in situ* generated **7**, were stirred under atmospheres of CO, in order to investigate if their steric differences had an influence on the outcomes of these reactions. This seemed to be the case, as the most hindered adduct, **8**, behaved similarly to **1**, in that it reductively trimerised CO to give a low isolated yield of the magnesium deltate complex, **9**, as a colourless crystalline solid (Scheme 2). In contrast, the two smaller adduct complexes, **6** and **7**, only consumed two equivalents of CO to give moderate isolated yields of the unusual, thermally stable ethenediolate complexes, **10** and **11**. These are presumably formed *via* an initial reductive dimerisation of CO, followed by activation of one of the *ortho*-C–H bonds of the coordinating DMAP molecule by the generated [C_2_O_2_]^2−^ fragment (see below). It is noteworthy that, when the progress of all of these reactions was monitored by ^1^H NMR spectroscopy, no intermediates in the formations of the CO coupled products were observed, and there was no evidence for mixtures of deltate or ethenediolate products in any case. Furthermore, treating the adduct complexes, **6–8**, with 1 : 1 mixtures of CO/H_2_ did not lead to involvement of dihydrogen in the reactions, which instead returned **9–11** in yields similar to those in its absence.^14^

Once crystallised, **9–11** are poorly soluble in most commonly used organic solvents. With that said, compounds **9** and **11** had sufficient solubility in *d*_8_-THF for their ^1^H and ^13^C NMR spectra to be recorded. The spectra for deltate complex, **9**, are consistent with its proposed formulation, and comparable to the spectra for **3**,^8^ in that they display two sets of signals for chemically inequivalent β-diketiminate ligands, and one set of DMAP resonances. The NMR spectra for **11** also exhibit two sets of β-diketiminate signals, while its ^1^H NMR spectrum shows three chemically inequivalent aromatic proton signals for the DMAP ligand, and a singlet resonance at *δ* 6.27 ppm for the ethenediolate proton.

**Fig. 2:**
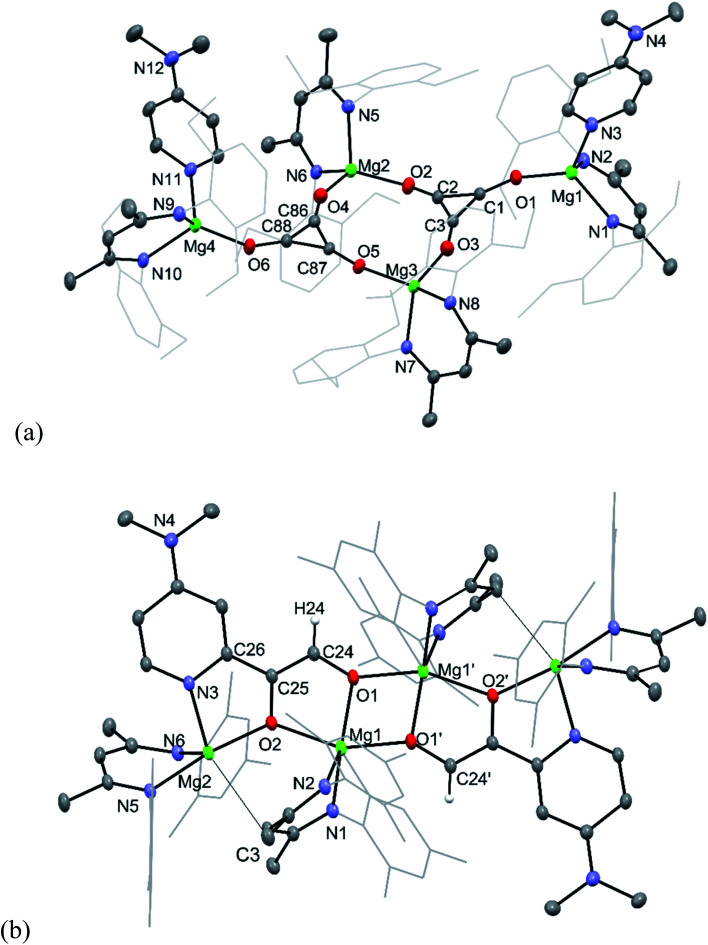
Molecular structures of (a) **9** and (b) **11** (25% thermal ellipsoids are shown; hydrogen atoms, except alkenic protons omitted; aryl substituents shown as wireframe for clarity). Selected bond lengths (Å) and angles (°) for **9**: Mg(1)–O(1) 1.882(3), O(1)–C(1) 1.279(4), C(1)–C(3) 1.391(6), C(1)–C(2) 1.396(5), Mg(2)–O(4) 1.899(3), Mg(2)–O(2) 1.904(3), O(2)–C(2) 1.273(5), C(2)–C(3) 1.399(6), Mg(3)–O(3) 1.898(3), Mg(3)–O(5) 1.907(3), O(3)–C(3) 1.278(5), Mg(4)–O(6) 1.877(3), O(4)–C(86) 1.276(5), O(5)–C(87) 1.274(4), O(6)–C(88) 1.276(5), C(86)–C(88) 1.393(6), C(86)–C(87) 1.398(6), C(87)–C(88) 1.385(5), C(1)–O(1)–Mg(1) 157.5(3), O(4)–Mg(2)–O(2) 116.28(14), O(3)–Mg(3)–O(5) 113.76(14), C(86)–O(4)–Mg(2) 156.0(3). Selected bond lengths (Å) and angles (°) for **11**: Mg(1)–O(1) 2.0035(14), Mg(1)–O(1)′ 2.0238(16), Mg(1)–O(2) 2.0963(15), Mg(2)–O(2) 1.9822(14), Mg(2)–N(3) 2.1512(18), Mg(2)–C(3) 2.838(2), O(1)–C(24) 1.334(2), O(2)–C(25) 1.365(2), N(3)–C(26) 1.359(2), C(24)–C(25) 1.352(3), C(25)–C(26) 1.453(3), O(1)–Mg(1)–O(1)′ 75.14(7), O(1)′–Mg(1)–O(2) 152.99(7), O(1)–Mg(1)–O(2) 80.11(6), O(2)–Mg(2)–N(3) 79.49(6).

The molecular structures of **9** and **11** are depicted in Fig. 2, while that for **10** can be found in the ESI.† As compound **9** is essentially isostructural to **3**,^8^ little comment will be passed on it here, except to point out that its deltate dianions are close to planar, with nearly equivalent C–C and C–O bond lengths, that lie between those for localised single and double bonds.^15^ It is apparent, therefore, that there is a significant degree of electronic delocalisation over the compound's aromatic deltate dianions.^16^ In contrast, the only other previously structurally characterised deltate complex of a non s-block metal, [{U(COT^†^)(Cp*)}_2_(μ-C_3_O_3_)] (COT^†^ = [C_8_H_6_Pr^i^_2_-1,4]^−^; Cp* = [C_5_Me_5_]^−^), exhibits differing C–C and C–O interactions, and partial electronic delocalisation over the dianion.^6*a*^

Compound **11** is isostructural to **10**, and its molecular structure reveals it to be a centrosymmetric dimer. The ethenediolate units possess localised C(24)–C(25) bonds, which are substituted in *cis*-positions by a proton and C(26) of the C–H activated DMAP unit. One magnesium centre Mg(1) of each monomeric unit is chelated by both *O*-centres of the ethenediolate, while Mg(2) is *N*,*O*-chelated by that dianion. Oxygen atoms O(1) coordinate Mg atoms on the opposing monomeric units, giving rise to a central four-membered Mg_2_O_2_ ring. The bond lengths within the N_2_C_3_ backbone of each chelating β-diketiminate suggest electronic delocalisation over those ligands. Magnesium atoms Mg(1) have distorted trigonal bipyramidal geometries, which is also the case for Mg(2), when the long interaction between that atom and C(3) of one of the β-diketiminate ligands (2.838(2) Å, *cf. Σ* van der Waals radii for Mg/C = 3.43 Å (ref. 17)) is taken into account.

It seems likely that the mechanism of formation of **9** is similar to that previously calculated for the closely related NHC coordinated deltate complex, **4**.^8^ In that case, there were two sequential insertions of CO into the Mg–Mg bond of the magnesium(i) starting material, yielding an intermediate with a *trans*-bent (“zig–zag”) [C_2_O_2_]^2−^ dianion, bridging two [(^Dip^Nacnac)(DMAP)_0 or 1_Mg]^+^ fragments. This reacts with a third CO molecule, ultimately leading to deltate complex **4**. Interestingly, a very similar mechanism, *via* a “zig–zag” intermediate, was computed by Cloke and co-workers for the formation of [{U(COT^†^)(Cp*)}_2_(μ-C_3_O_3_)].^6*f*,18^ They also showed that controlling the stoichiometry of the reaction between [U(COT^†^)(Cp*)(THF)] and CO led to the linear ethynediolate system, [{U(COT^†^)(Cp*)}_2_(μ-OC≡CO)], which did not react with CO to give [{U(COT^†^)(Cp*)}_2_(μ-C_3_O_3_)].^6*f*^ This result gave credence to the importance of the “zig–zag” intermediate in the formation of the latter complex. That is not to say that uranium ethynediolate complexes are unreactive, as Arnold and co-workers showed when they heated solutions of [{[(Me_3_Si)_2_N]_2_U}_2_(μ-OC≡CO)]. This led to an intramolecular C–H activation of one of its methyl substituents, yielding an ethenediolate species, related to **10** and **11**.^6*g*^ This raised the question as to whether the mechanism of formation of **10** and **11** proceeds *via* reactive ethynediolate intermediates, [{(^Ar^Nacnac)Mg}_2_(μ-OC≡CO)], or by another process.

**Fig. 3:**
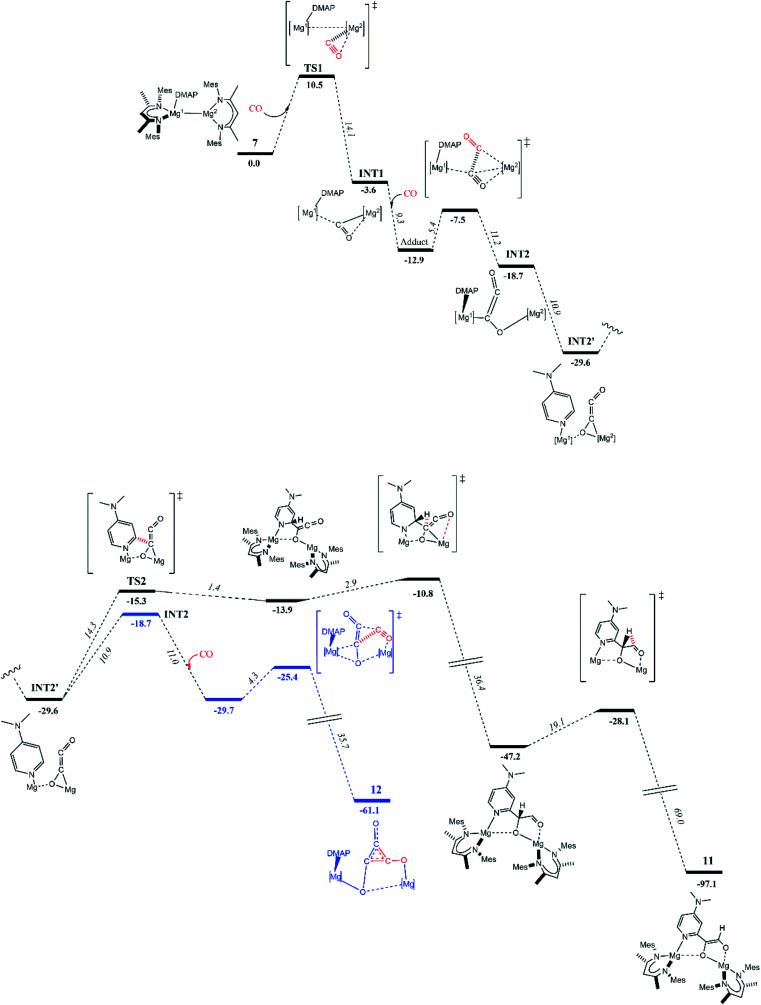
Computed (B3PW91) enthalpy profile at 298 K for the formation of ethenediolate complex **11**, or deltate complex **12**, from magnesium(i)-adduct complex **7**, and two or three molecules of CO, respectively.

To explore these possibilities, DFT calculations (B3PW91) were carried out to determine the reaction profile that led to **11** (Fig. 3). The initial stages of the reaction were found to be similar to that calculated for the formation of **4**.^8^ That is, the first step involves nucleophilic attack of the three-coordinate Mg^2^ centre of polarized **7** (Natural Bond Orbitals, NBO, charges: Mg^1^ 0.45; Mg^2^ 0.19) at one of the π*-orbitals of CO (see ESI† for further details), giving adduct **TS1** (10.5 kcal mol^−1^). The coordinated CO then inserts into the Mg–Mg bond affording stable intermediate **INT1** (−3.6 kcal mol^−1^). From **INT1**, a second CO insertion leads to a “zig–zag” intermediate **INT2** (−18.7 kcal mol^−1^) *via* a low kinetic barrier (5.4 kcal mol^−1^). It is of note that an alternative pathway was explored, whereby the DMAP C–H activation process occurred from **INT1**, but this was found not to be kinetically viable (barrier = 21.4 kcal mol^−1^). Instead, the favoured intermediate **INT2** readily isomerised to the more stable (by 10.9 kcal mol^−1^) “zig–zag” intermediate **INT2′**. Interestingly, this isomer appears more amenable to DMAP C–H activation, as one DMAP *ortho*-proton is pointing in the direction of the [C_2_O_2_]^2−^ moiety. From **INT2′**, two kinetically reasonable pathways were examined. Firstly, isomerization back to **INT2** and reaction with a third molecule of CO led to the deltate complex **12** (−61.1 kcal mol^−1^) *via* a pathway similar to that calculated for the formation of **4** (blue pathway). Secondly, **INT2′** undergoes an intramolecular DMAP C–H activation *via* a number of kinetically accessible steps, ultimately giving the experimentally observed product, **11** (black pathway). While compound **11** is significantly more stable (by 31.4 kcal mol^−1^) than the alternative deltate product, **12**, the overall kinetic barrier to its formation is higher.

Taken as a whole, the experimental and computational studies indicate that **11** is the thermodynamic product of the reaction of **7** with excess CO, while deltate complex **12**, is the kinetic product. Despite this, in the experimental situation, compound **11** is formed in preference to the deltate complex, **12**, even when the reaction that afforded it is carried out at low temperature. Moreover, it is clear that **11** is not formed *via* an ethynediolate intermediate, [{(^Mes^Nacnac)Mg}_2_(μ-OC≡CO)] (which was never experimentally observed), in contrast to Arnold's aforementioned report on intramolecular C–H activation of a uranium ethynediolate complex.^6*g*^ It can additionally be speculated from the results of these and prior calculations, that ethenediolate complexes, **10** and **11**, result when less hindered activated magnesium(i) adduct complexes, **6** and **7**, are treated with CO, because an *ortho*-C–H bond of the ligating DMAP molecule can approach the [C_2_O_2_]^2−^ fragment in the “zig–zag” transition state **TS2**, more readily than in reactions of **1** and **8** with CO. If so, in those latter cases, the kinetic barrier to ethenediolate formation should be raised sufficiently to favour formation of the experimentally observed deltate complexes, **3** and **9**. In the case of the extremely bulky magnesium(i) compound **5**, no DMAP adduct can be formed, but its Mg–Mg bond is very long, yet it does not react with CO under ambient conditions. This suggests that the enhanced reactivity of 1 : 1 adduct complexes **1** and **2**, and **6–8**, towards CO arises more from the polarised nature of their Mg–Mg bonds, than the elongation of those bonds.

## Conclusions

In summary, an extremely bulky, symmetrical three-coordinate magnesium(i) complex has been prepared and shown to have a very long Mg–Mg bond for a such a species. This does not react with either DMAP or CO. Three 1 : 1 DMAP adducts of less bulky Mg–Mg bonded species have been prepared (one *in situ*), and their enhanced reactivity toward CO explored. It was found that when the compounds incorporate bulkier β-diketiminate ligands, they reductively trimerise CO to give magnesium deltate complexes. When substituted with smaller β-diketiminates, the magnesium(i) adducts react with only two CO molecules, ultimately giving unusual ethenediolate complexes. DFT calculations show that these reactions proceed *via* reductive dimerization of CO, and subsequent intramolecular C–H activation of Mg-ligated DMAP by “zig–zag” [C_2_O_2_]^2−^ fragments of reaction intermediates. It is apparent that magnesium deltate complexes are kinetic products in these reactions, while magnesium ethenediolates are thermodynamic products. As a result, subtle changes to the bulk of the 1 : 1 DMAP–magnesium(i) adducts can lead to fine steric control over the products arising from their CO reductive oligomerisations. We continue to investigate the reactivity of activated magnesium(i) compounds towards CO and other small molecules, and how selectivity in the products of those reactions can be achieved.

## Conflicts of interest

There are no conflicts to declare.

## Supplementary Material

SC-011-D0SC00836B-s001

SC-011-D0SC00836B-s002
