# Supplementary material for: Sterically controlled reductive oligomerisations of CO by activated magnesium(i) compounds: deltate vs. ethenediolate formation
Source: Chem Sci. 2020 Mar 6;11(13):3516–22. doi: 10.1039/d0sc00836b (PMC8152598; doi:10.1039/d0sc00836b)
Supplement: SC-011-D0SC00836B-s001 [file SC-011-D0SC00836B-s001.pdf]

Electronic Supplementary Material (ESI) for Chemical Science.
This journal is © The Royal Society of Chemistry 2020

Sterically Controlled Reductive Oligomerisations of CO by Activated Magnesium(I) Compounds: Deltate *vs.* Ethenediolate Formation

K. Yuvaraj,[a] Iskander Douair,[b] Dafydd D. L. Jones,[a] Laurent Maron*,[b] and Cameron Jones*,[a]

[a] School of Chemistry, PO Box 23, Monash University, VIC, 3800, Australia.
[b] Université de Toulouse et CNRS, INSA, UPS, UMR 5215, LPCNO, 135 Avenue de Rangueil, F-31077 Toulouse, France.

Electronic Supplementary Information (75 pages)

Contents

**1. Experimental**	**S2**
**2. X-Ray Crystallographic Studies**	**S26**
**3. Computational Studies**	**S34**
**4. References**	**S74**

1. Experimental

General considerations.

All manipulations were carried out using standard Schlenk and glove box techniques under an atmosphere of high purity dinitrogen. Pentane and diethyl ether were distilled over Na/K alloy (50:50), while hexane, cyclohexane, toluene and THF were distilled over molten potassium. ^{1}H and ^{13}C{^{1}H} NMR spectra were recorded on Bruker Avance III 400 or Bruker Avance III 600 spectrometers and were referenced to the resonances of the solvent used or external $SiMe_4$. Mass spectra were collected using an Agilent Technologies 5975D inert MSD with a solid-state probe. FTIR spectra were collected for solid samples or Nujol mulls on an Agilent Cary 630 attenuated total reflectance (ATR) spectrometer. Microanalyses were carried out at the Science Centre, London Metropolitan University. Melting points were determined in sealed glass capillaries under dinitrogen, and are uncorrected. The compounds (TCHP)NH_2,[1] and [{(^{Ar}Nacnac)Mg}$_2$] (Ar = Xyl[2], Mes[3] or Dep[4]), were prepared according to the literature procedures. CO gas was dried over P_2O_5 in prior to use. All other reagents were used as received.

Synthesis of ^{TCHP}NacnacH. 2,4,6-tricyclohexylaniline (10.0 g, 29.5 mmol), p-tolylsulfonic acid monohydrate (2.80 g, 14.7 mmol) and acetylacetone (1.50 mL, 14.7 mmol) were dissolved in toluene (150 mL) in a round bottom flask. A Dean-Stark apparatus was attached and the mixture heated at reflux for 72h. After cooling, NEt_3 (2.1 mL, 15.0 mmol) was added and the mixture allowed to stir for 1h. The organic phase was washed with water (2 x 30 mL), dried over $MgSO_4$ and evaporated to yield a dark red oil. Cold methanol was added to the oil to yield the title compound as an off-white solid after filtration and drying (9.16 g, 83 %). Crystals suitable for X-ray crystallographic studies were obtained by slow evaporation of a solution of ^{TCHP}NacnacH in diethyl ether. M.p. 103-105 °C. ^{1}H NMR (400 MHz, C_6D_6, 298 K): N.B. integration of resonances for cyclohexyl groups are estimated due to complex overlapping signals, and small amounts of unknown impurities. δ 1.21-2.03 (m, 60H, Cy-CH_2), 1.72 (s, 6H, NCCH_3), 2.52-2.59 (m, 2H, Cy-C*H*), 2.98-3.05 (m, 4H, Cy-C*H*), 4.84 (s, 1H, NCC*H*), 7.18 (s, 4H, Ar*H*), 11.63 (br, 1H, N*H*); ^{13}C{^{1}H} (101 MHz, C_6D_6, 298 K): δ 21.4 (NC*C*H_3), 26.9, 27.5, 27.6, 27.7, 34.5, 35.3, 39.3, 45.2 (Cy-*C*), 94.5 (NC*C*H), 123.0, 139.5, 141.6, 144.6 (Ar*C*), 161.4 (N*C*CH_3); IR ν/cm^{-1} (ATR): 1654 (m), 1617 (m), 1545 (m), 1492 (m), 1117 (m), 1076 (w), 1029 (w), 949 (w), 920

(w), 861 (m), 797 (w), 777 (w), 744 (m), 699 (m); acc. mass/ESI *m/z*: calc. for $[M+H]^+$ 743.6238 found: 743.6231.

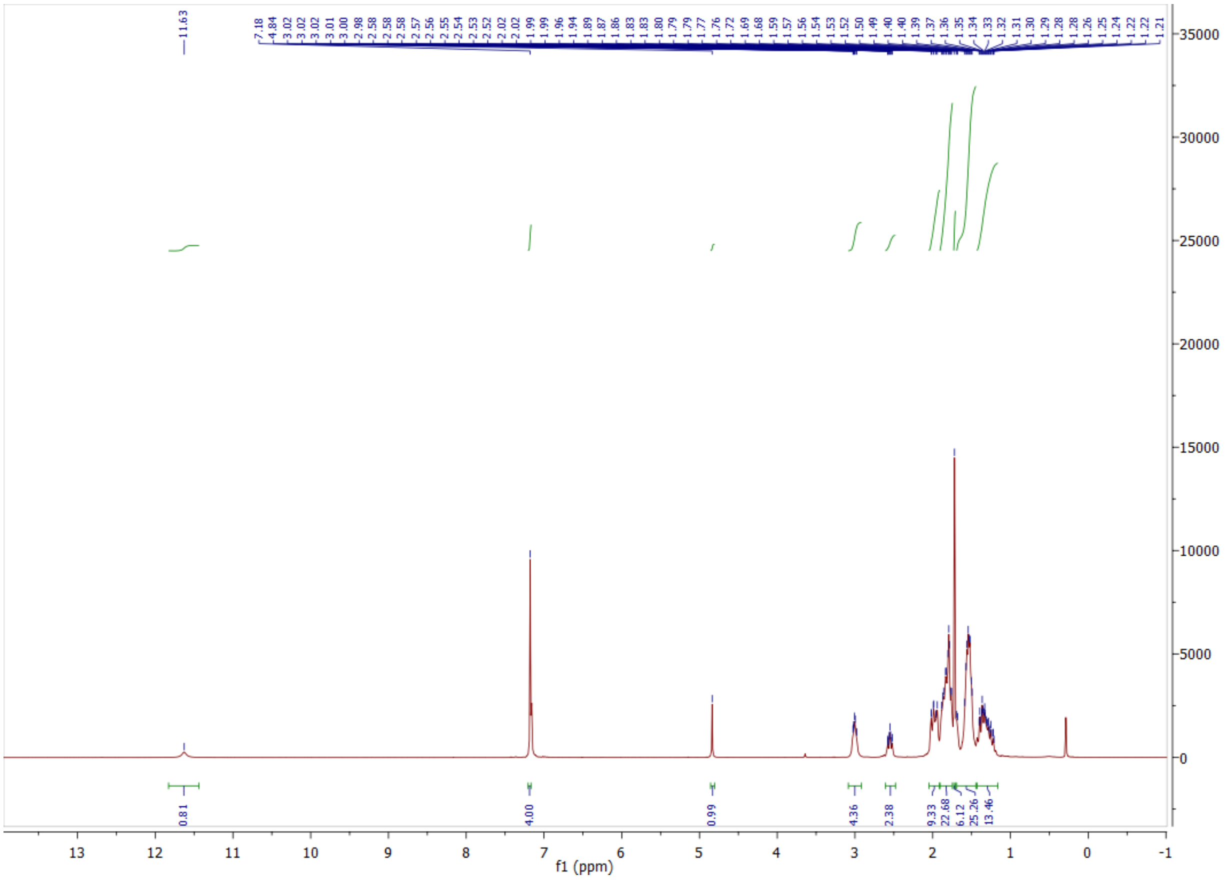

Figure S1. ^{1}H NMR spectrum (400 MHz, 298 K, C_6D_6) of ^{TCHP}NacnacH.

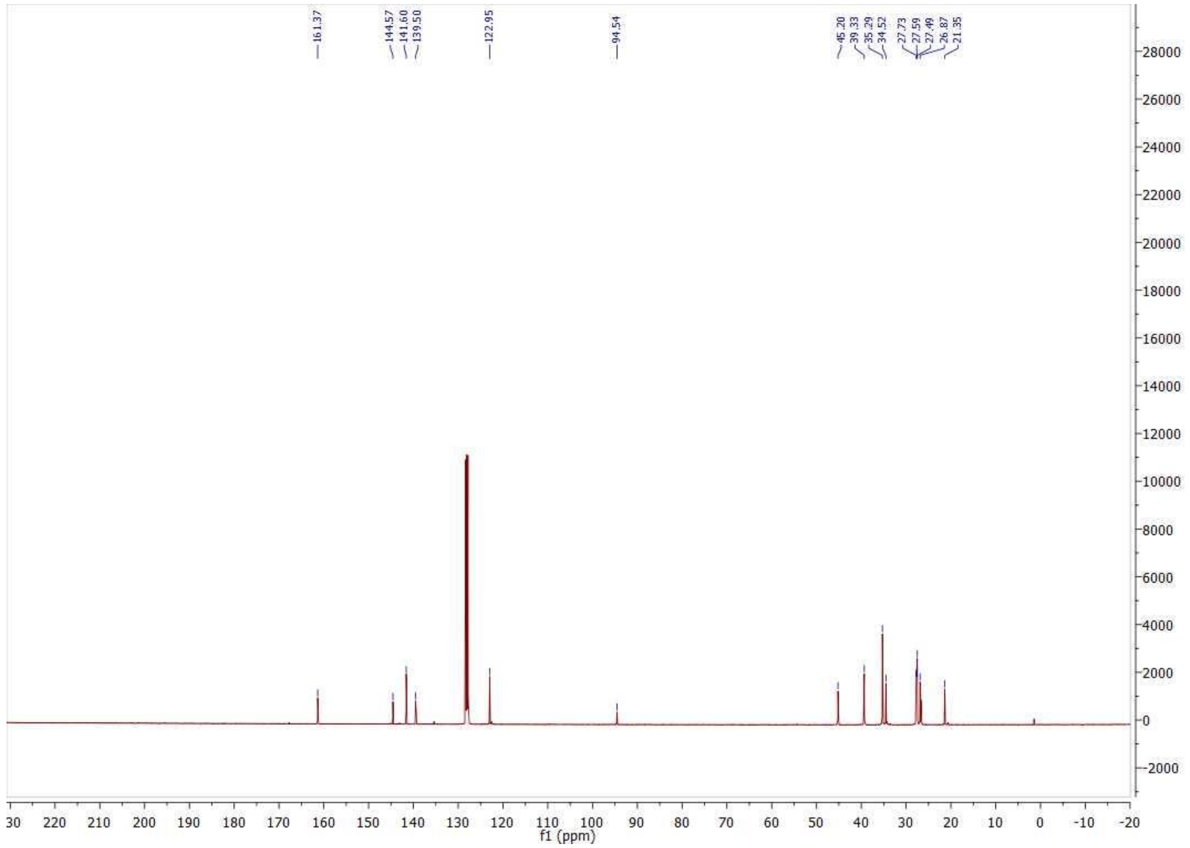

Figure S2. $^{13}C\{^1H\}$ NMR spectrum (101 MHz, 298 K, C_6D_6) of ^{TCHP}NacnacH.

Synthesis of [(^{TCHP}Nacnac)MgI(OEt_2)]. Mg turnings (78 mg, 3.33 mmol) were placed in a Schlenk flask and placed under vacuum. After 20 minutes, diethyl ether (5 mL) and a crystal of I_2 were added. After the iodine had been consumed, a reflux condenser was added and MeI (0.234 mL, 3.76 mmol) in diethyl ether (5 mL) was added. The resultant suspension was heated at reflux for 4h. The freshly prepared MeMgI solution was cooled and was subsequently added to a suspension of ^{TCHP}NacnacH (2.00 g, 2.69 mmol) in diethyl ether (20 mL), and the mixture stirred overnight. The resultant solution was filtered, concentrated *in vacuo* and stored at –30 °C to yield colourless crystals of the title compound. A second crop of crystals could be obtained from further concentration and storage of the supernatant solution at –30 °C (1.44 g, 55 %). M.p. 162-165 °C (decomp): ^{1}H NMR (400 MHz, C_6D_6, 298 K) N.B. integrations for cyclohexyl groups are estimated due to complex overlapping signals: δ 1.11 (t, $^3J_{HH}$ = 7.0 Hz, 6H, OCH_2CH_3), 1.32-1.45 (m, 11H, Cy-*H*), 1.48 (s, 6H, $NCCH_3$), 1.51-2.00 (m, 45H, cyclohexyl CH_2), 2.27-2.31 (m, 4H, Cy-CH_2), 2.69-2.74 (m, 2H, Cy-C*H*), 2.79-2.86 (m, 4H, Cy-C*H*), 3.26

(q, $^3J_{HH}$ = 7.0 Hz, 4H, $CH_3C\textit{H}_2O$), 4.64 (s, 1H, NCC*H*), 7.14 (s, 4H, Ar*H*); $^{13}C\{^1H\}$ (101 MHz, C_6D_6, 298 K): δ 15.5 (*C*H_3CH_2O), 25.3 (NC*C*H_3), 26.9, 27.0, 27.7, 27.9, 28.3, 34.7, 35.2, 35.9, 40.1, 44.8 (Cy-*C*), 66.0 (CH_3*C*H_2O), 95.2 (NC*C*H), 123.1, 141.4, 142.8, 144.0 (Ar*C*), 170.7 (N*C*CH_3); IR ν/cm^{-1} (Nujol): 1618 (m), 1546 (s), 1143 (w), 1115 (w), 1086 (w), 1017 (w), 997 (w), 949 (w), 925 (w), 890 (w), 862 (m), 842 (w); EI/MS (70eV) m/z (%): 83.1 (Cy^+, 23), 364.3 $Cy_3C_6H_2NCMe^+$, 100), 659.6 (^{TCHP}NacnacH–Cy^+, 13), 727.8 (^{TCHP}NacnacH-CH_3^+, 23), 742.8 (^{TCHP}NacnacH$^+$, 13); anal. calc. for $C_{57}H_{87}IMgN_2O$: C 70.76 %, H 9.06 %, N 2.90 %; found: C 70.66 %, H 8.86 %, N 2.83 %.

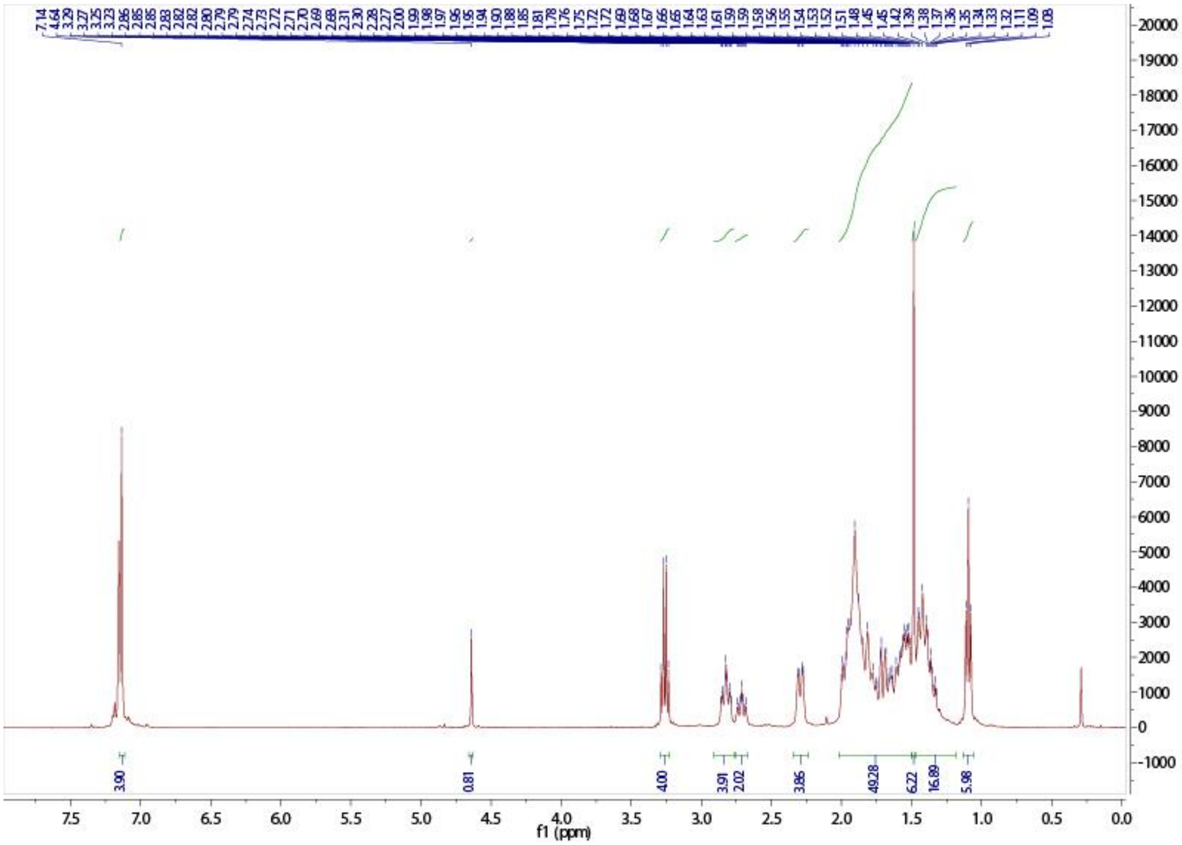

Figure S3. ^1H NMR spectrum (400 MHz, 298 K, C_6D_6) of [(^{TCHP}Nacnac)MgI(OEt_2)].

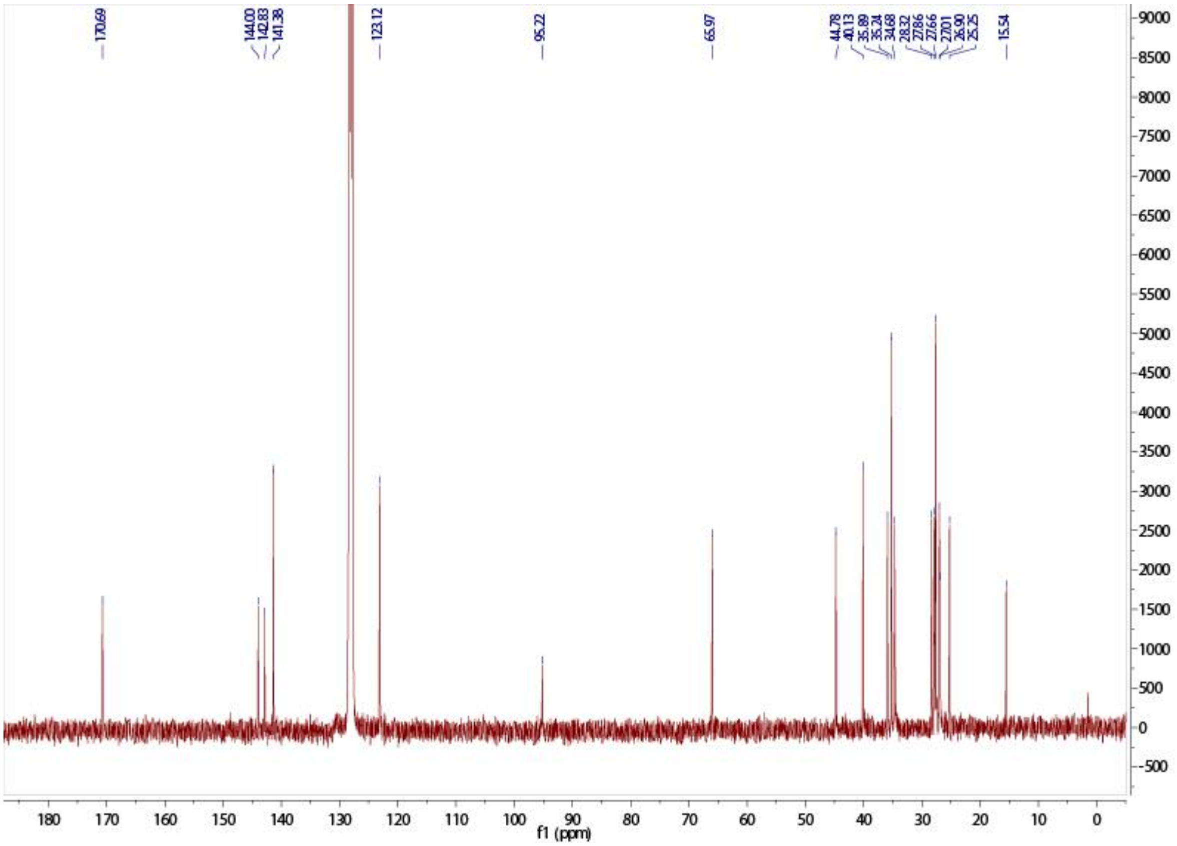

Figure S4. $^{13}C\{^1H\}$ NMR spectrum (101 MHz, 298 K, C_6D_6) of [(^{TCHP}Nacnac)MgI(OEt_2)].

Synthesis of [{(^{TCHP}Nacnac)Mg}$_2$], 5. [(^{TCHP}Nacnac)MgI(OEt_2)] (700 mg, 0.72 mmol) was dissolved in a 4:1 mixture of toluene/diethyl ether (20 mL/5 mL) and the solution stirred over a sodium mirror (120 mg, 7.2 mmol) at room temperature. The progress of the reaction was monitored by ^1H NMR spectroscopy until all [(^{TCHP}Nacnac)MgI(OEt_2)] was consumed (*ca.* 96h), after which the solution was filtered, the filtrate concentrated *in vacuo* and stored at –30 °C to yield yellow crystals of [{(^{TCHP}Nacnac)Mg}$_2$]. A second crop was obtained from further concentration of the supernatant solution and storage at –30 °C (405 mg, 73 %). M.p. > 260 °C. ^1H NMR (400 MHz, C_6D_6, 298 K) N.B. integration for cyclohexyl groups are estimated due to complex overlapping signals: δ 1.21-1.41 (m, 20H, Cy-CH_2), 1.44 (s, 12H, NCCH_3), 1.46-1.60 (m, 40H, Cy-CH_2), 1.67-2.01 (m, 52H, Cy-CH_2), 2.34-2.38 (br. m, 8H, Cy-CH_2), 2.69-2.77 (br. m, 12H, Cy-C*H*), 4.72 (s, 2H, NCC*H*), 7.12 (s, 8H, Ar*H*); $^{13}C\{^1H\}$ NMR (101 MHz, C_6D_6, 298 K) δ 25.2 (NC*C*H_3), 26.9, 27.0, 27.7, 28.1, 28.5, 33.9, 35.1, 35.2, 39.9, 44.6 (Cy-*C*), 95.7

(NC*C*H), 123.0, 141.1, 143.3, 145.2 (Ar*C*), 168.5 (N*C*CH$_3$); IR ν/cm^{-1} (ATR): 1528 (m), 1115 (m), 1069 (m), 1028 (w), 992 (w), 920 (w), 727 (m), 695 (s); EI/MS (70eV) m/z (%): 83.1 (Cy$^+$, 22), 364.3 Cy$_3$C$_6$H$_2$NCMe$^+$, 100), 659.6 (^{TCHP}NacnacH–Cy$^+$, 11), 727.8 (^{TCHP}NacnacH-CH$_3$$^+$, 21), 742.8 (^{TCHP}NacnacH$^+$, 13); anal. calc. for $C_{106}H_{154}Mg_2N_4$: C 83.05 %, H 10.13 %, N 3.65 %; found: C 82.88 %, H 9.92 %, N 3.55 %.

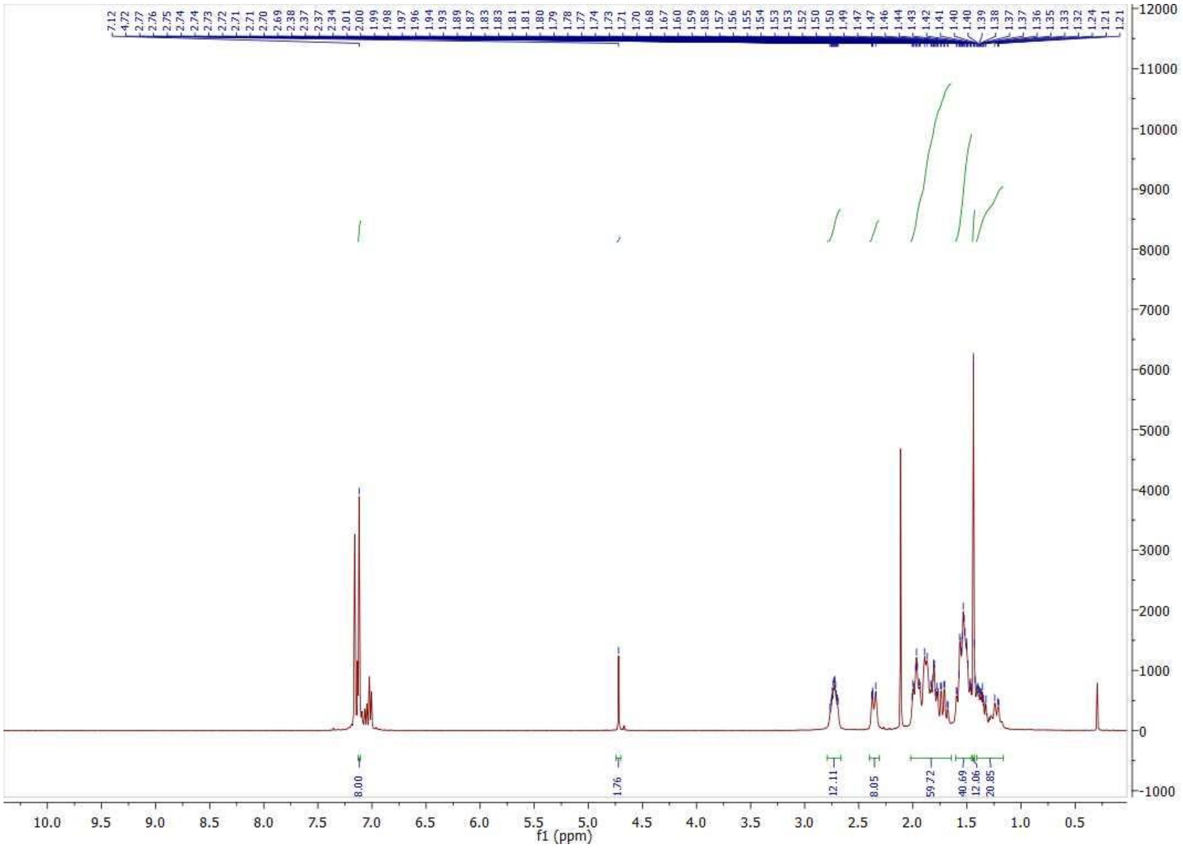

Figure S5. ^{1}H NMR spectrum (400 MHz, 298 K, C_6D_6) of [{(^{TCHP}Nacnac)Mg}$_2$].

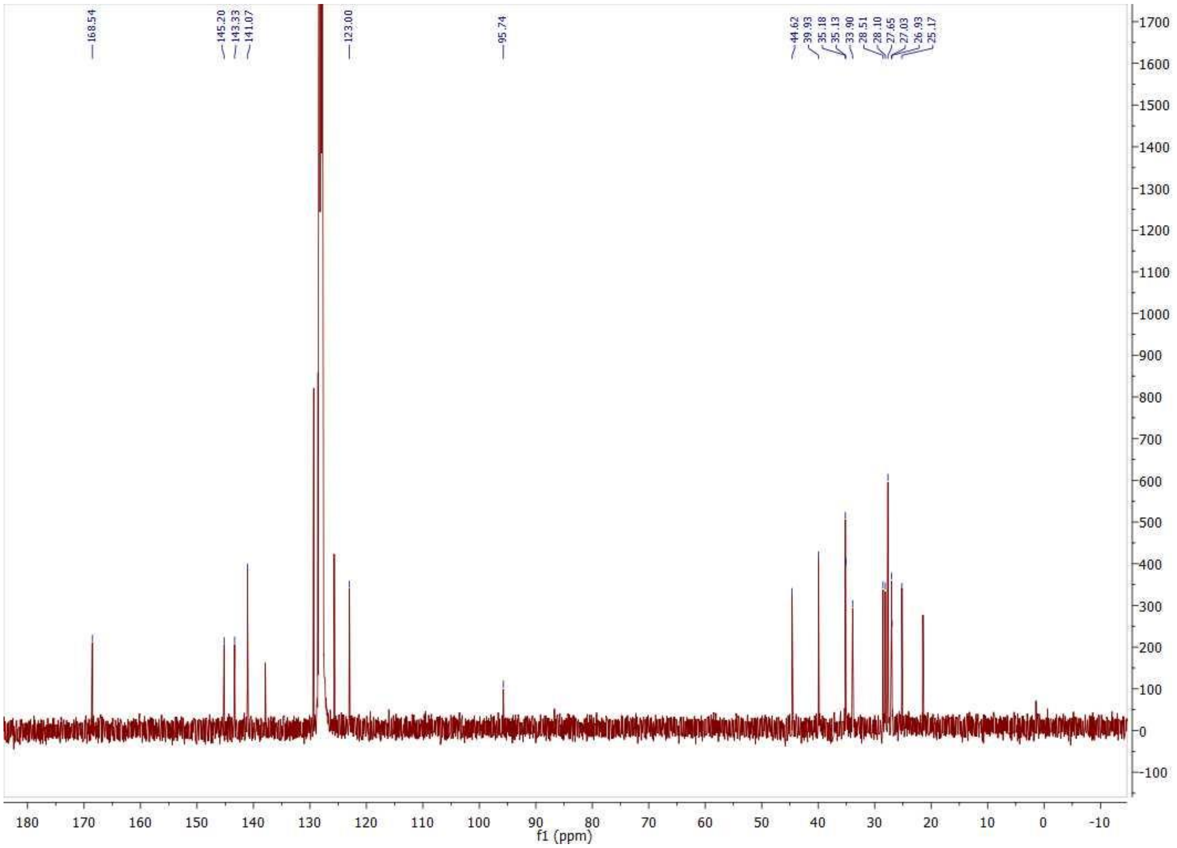

Figure S6. $^{13}C\{^1H\}$ NMR spectrum (101 MHz, 298 K, C_6D_6) of [{(^{TCHP}Nacnac)Mg}$_2$].

Synthesis of [(^{Xyl}Nacnac)(DMAP)Mg−Mg(^{Xyl}Nacnac)], 6. [{(^{Xyl}Nacnac)Mg}$_2$] (200 mg, 0.304 mmol) and DMAP (37 mg, 0.304 mmol) were dissolved in toluene (10 mL) at -78 °C. This resulted in an orange-red solution. The mixture was stirred for 1h, warmed to room temperature, filtered, and the filtrate concentrated to *ca.* 3 mL *in vacuo* and layered with hexane. The filtrate was then placed at -30 °C for 2 d, after which time red-orange crystals of **6** had deposited. These were isolated and a second crop obtained from the mother liquor (129 mg, 54 %). M.p: 157-160 °C; ^{1}H NMR (600 MHz, toluene-d_8, 298 K) δ 1.59 (s, 12H, NCCH_3), 1.94 (s, 24H, *ortho*-CH_3), 2.19 (s, 6H, N(CH_3)$_2$), 4.85 (s, 2H, C*H*), 5.98 (d, $^3J_{HH}$= 6.1 Hz, 2H, DMAP-Ar*H*), 6.96-7.10 (m, 12H, Ar*H*), 7.90 (d, $^3J_{HH}$ = 6.0 Hz, 2H, DMAP-Ar*H*); $^{13}C\{^1H\}$ NMR (151 MHz, toluene-d_8, 298 K) δ 20.1 (*ortho*-CH$_3$), 24.0 (NCCH_3), 38.4 (N(CH$_3$)$_2$), 94.9 (*C*H), 106.5 (DMAP-Ar*C*), 123.8, 128.7, 132.7, 149.8 (Ar*C*), 150.5, 154.9 (DMAP-Ar*C*), 165.4 (N*C*CH$_3$); IR v/cm^{-1} (Nujol): 1610 (m), 1517 (m), 1266 (m), 1225 (m), 1178 (s), 1005 (m), 833 (m), 755 (s); MS (EI, 70 eV): *m*/*z* (%) = 659.6 ({(^{Xyl}Nacnac)Mg}$_2^+$, 5), 329.2 ({(^{Xyl}Nacnac)Mg}$^+$, 68), 146.1 (MeCNXyl$^+$, 100);

anal. calc. for $C_{49}H_{60}Mg_2N_6$: C 75.29 %, H 7.74 %, N 10.75 %: found: C 75.19 %, H 7.91 %, N 10.56 %.

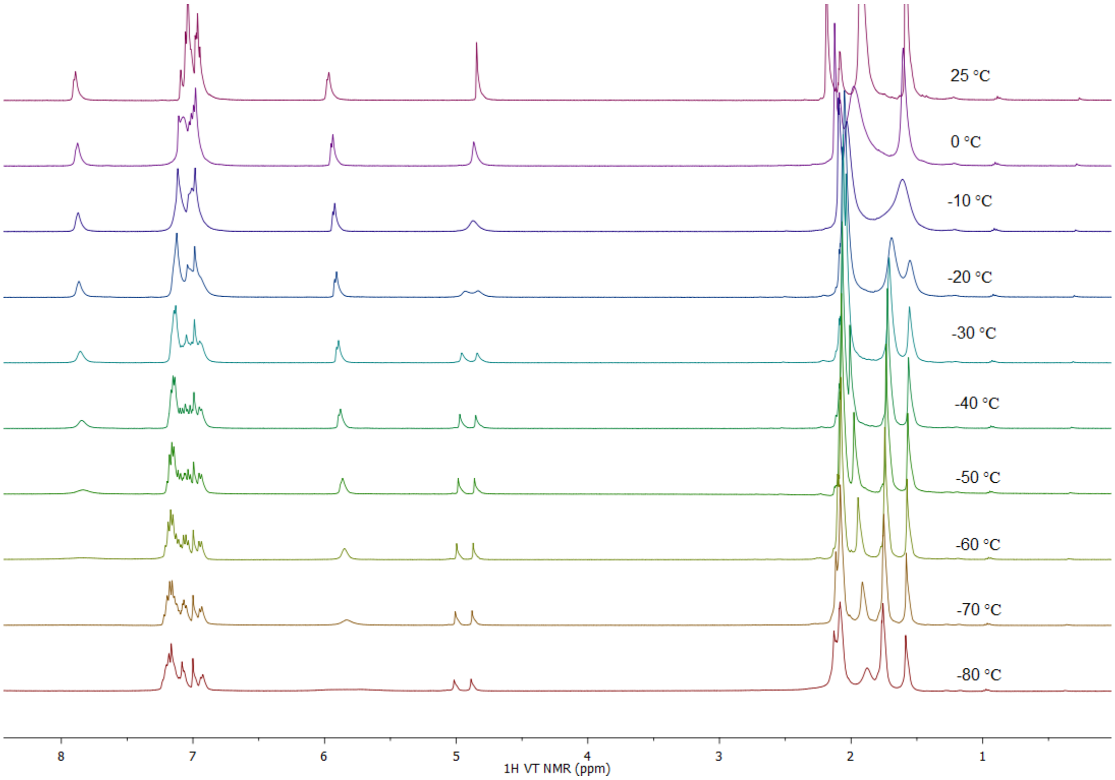

Figure S7. Varaible temperature ^{1}H NMR spectra (400 MHz, toluene-d_8) of **6**.

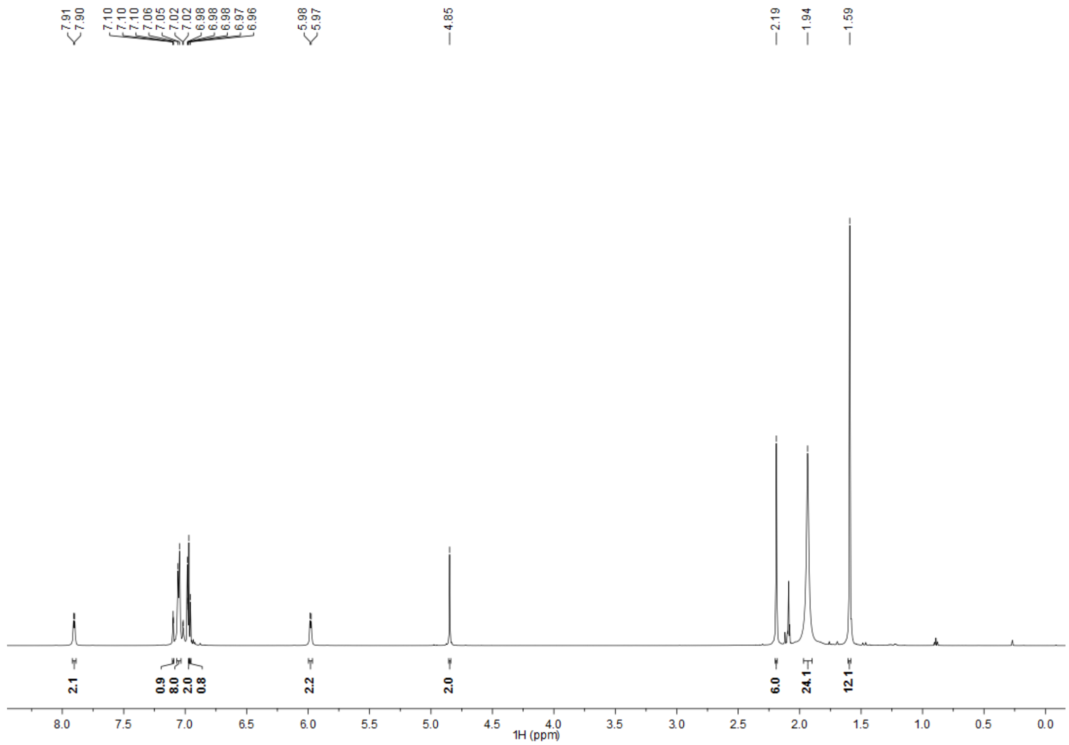

Figure S8. ^{1}H NMR spectrum (600 MHz, 298 K, toluene-d_8) of **6**.

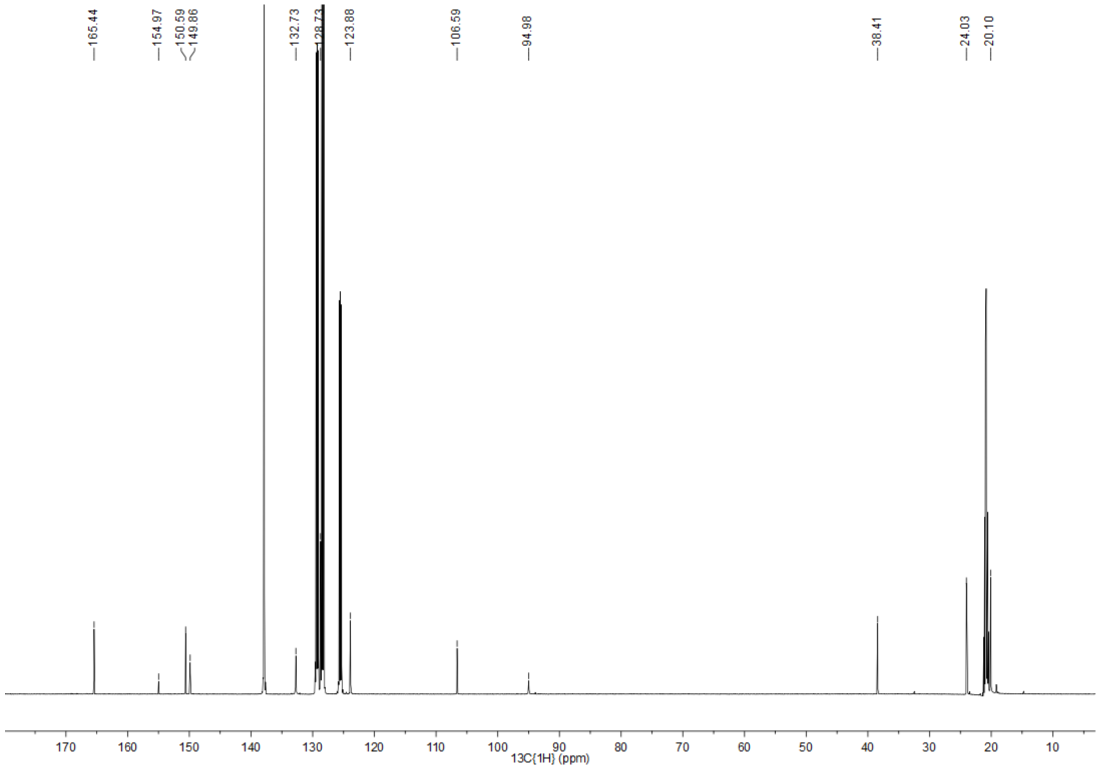

Figure S9. ^{13}C{^{1}H} NMR spectrum (151 MHz, 298 K, toluene-d_8) of **6**.

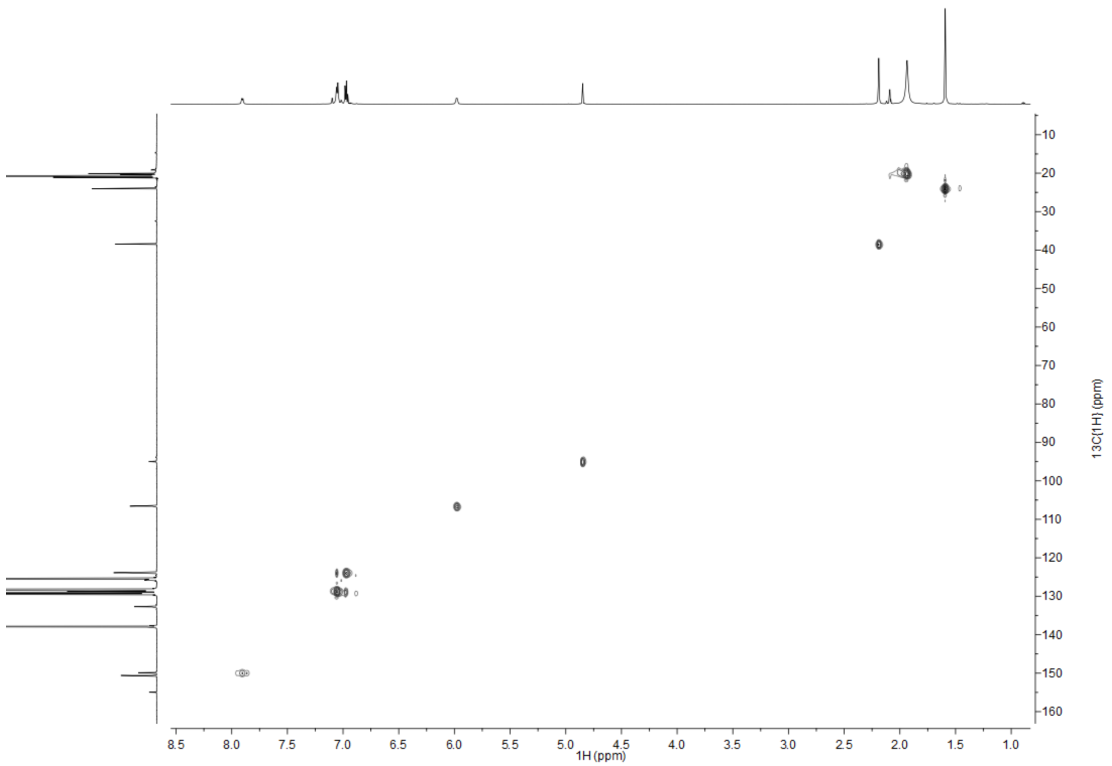

Figure S10. HMQC spectrum (^{1}H: 600 MHz; ^{13}C: 151 MHz, 298 K, toluene-d_8) of **6**.

Synthesis of [{(^{Xyl}Nacnac)Mg(DMAP)}$_2$]. [{(^{Xyl}Nacnac)Mg}$_2$] (150 mg, 0.228 mmol) and DMAP (56 mg, 0.456 mmol) were dissolved in toluene (8 mL) at room temperature. This resulted in an intense red solution. The mixture was stirred for 1h, filtered, and the filtrate concentrated to *ca.* 4 mL *in vacuo*. The filtrate was then placed at -30 °C for 1 d, after which time dark red crystals of the title compound had deposited. These were isolated and a second crop obtained from the mother liquor (111 mg, 54 %). M.p. 148-151 °C; ^{1}H NMR (400 MHz, C_6D_6, 298 K) δ 1.69 (s, 12H, NCCH_3), 2.02 (s, 24H, *ortho*-CH_3), 2.16 (s, 12H, N(CH_3)$_2$), 4.95 (s, 2H, C*H*), 6.04 (d, $^{3}J_{HH}$ = 6.0 Hz, 4H, DMAP-Ar*H*), 7.00 (d, $^{3}J_{HH}$ = 1.7 Hz, 2H, Ar*H*), 7.05 (d, $^{3}J_{HH}$ = 6.0 Hz, 4H, Ar*H*), 7.11 (s, 6H, Ar*H*), 8.23 (d, $^{3}J_{HH}$ = 5.9 Hz, 4H, DMAP-Ar*H*); $^{13}C\{^{1}H\}$ NMR (101 MHz, C_6D_6, 298 K) δ 19.9 (*ortho*-*C*H_3), 23.9 (NCCH_3), 38.1 (N(*C*H_3)$_2$), 94.2 (*C*H), 106.4 (DMAP-Ar*C*), 123.3, 128.1, 132.6 (Ar*C*), 150.0 (DMAP-Ar*C*), 150.9 (Ar*C*), 154.3 (DMAP-Ar*C*), 164.7 (N*C*CH_3); IR *v*/cm^{-1} (Nujol): 1610 (s), 1545, 1517 (w), 1268 (m), 1177 (m), 1092 (w), 1005 (m), 806 (s), 760 (m); MS (EI, 70 eV): *m*/*z* (%) = 329.2 ({(^{Xyl}Nacnac)Mg}$^{+}$, 28),

146.1 (MeCNXyl$^+$, 100); anal. calc. for $C_{56}H_{70}Mg_2N_8$: C 74.42 %, H 7.81 %, N 12.40 %: found: C 74.12 %, H 7.96 %, N 12.22 %.

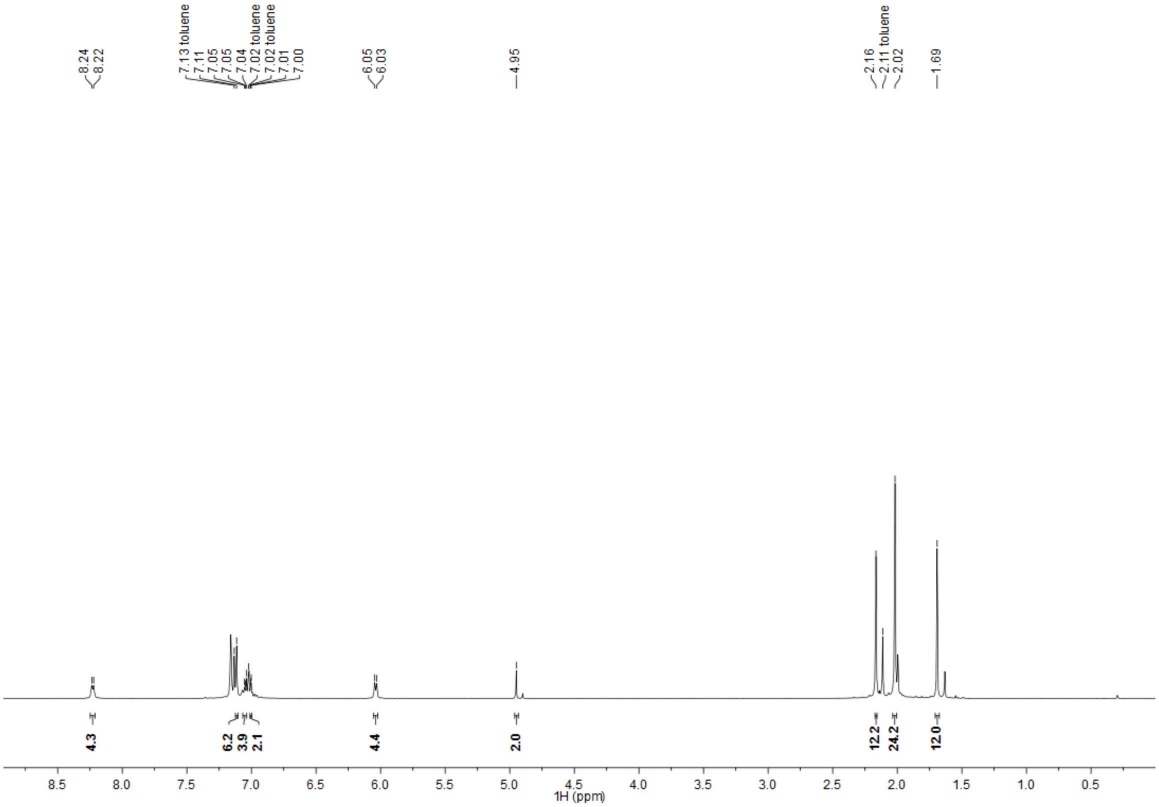

Figure S11. ^{1}H NMR spectrum (400 MHz, 298 K, C_6D_6) of [{(^{Xyl}Nacnac)Mg(DMAP)}$_2$].

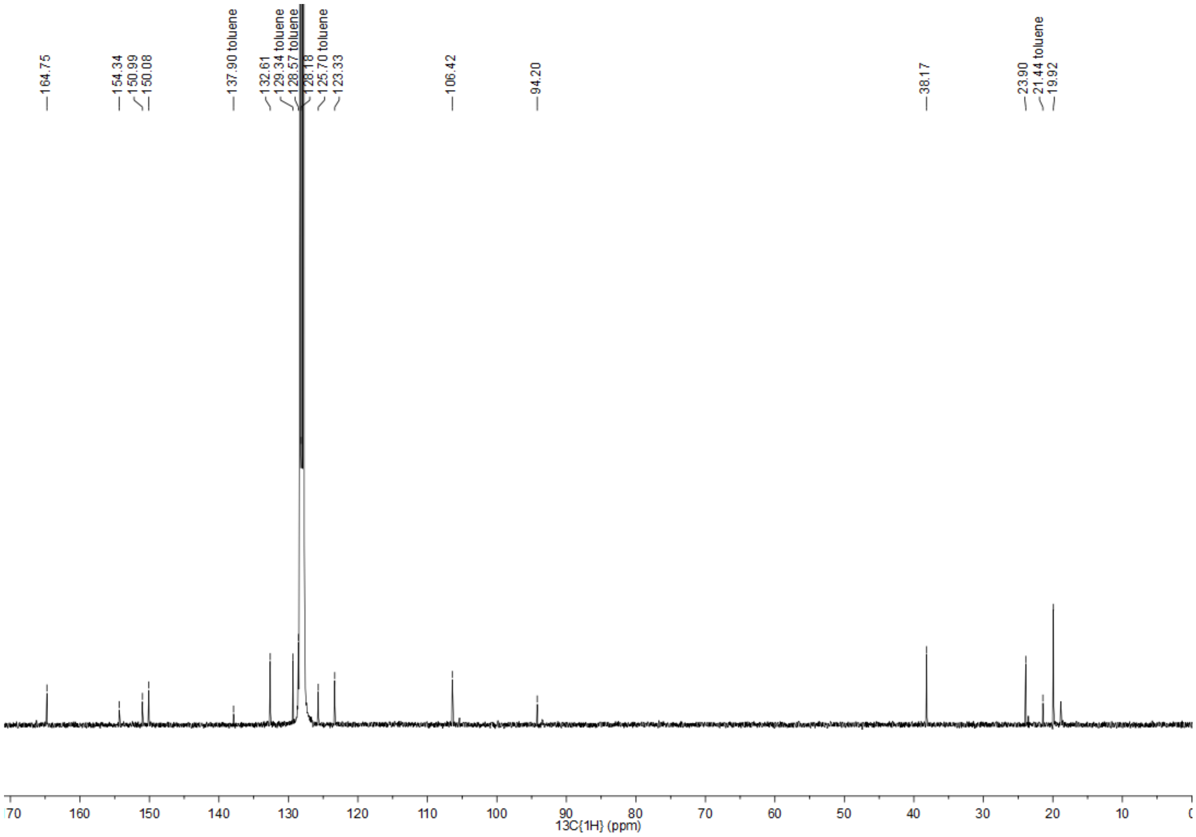

Figure S12. $^{13}C\{^{1}H\}$ NMR spectrum (101 MHz, , 298 K, C_6D_6) of [{(^{Xyl}Nacnac)Mg(DMAP)}$_2$].

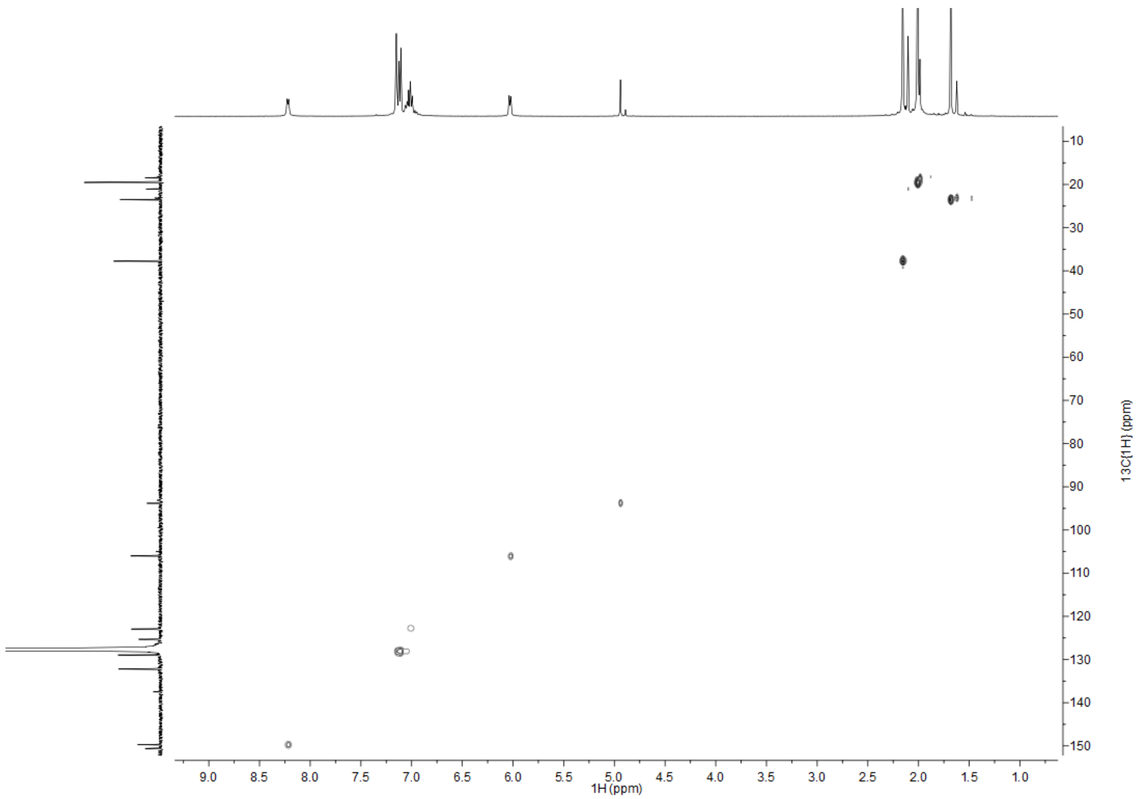

Figure S13. HMQC spectrum (^{1}H: 400 MHz; ^{13}C: 101 MHz, 298 K, C_6D_6) of [{(^{Xyl}Nacnac)Mg(DMAP)}$_2$].

Synthesis of [(^{Dep}Nacnac)(DMAP)Mg−Mg(^{Dep}Nacnac)], 8. [{(^{Dep}Nacnac)Mg}$_2$] (150 mg, 0.195 mmol) and DMAP (24 mg, 0.195 mmol) were dissolved in toluene (8 mL) at -78 °C. This resulted in an orange-red solution. The mixture was stirred for 1h, warmed to room temperature, filtered, and the filtrate concentrated to *ca.* 2 mL *in vacuo* and layered with hexane. The filtrate was then placed at -30 °C for 3 d, after which time red-orange crystals of **8** had deposited. These were isolated and a second crop obtained from the mother liquor (71 mg, 41 %). M.p. 124-127 °C; ^{1}H NMR (400 MHz, C_6D_6, 298 K) δ 1.11 (t, $^3J_{HH}$ = 7.5 Hz, 24H, CH_2CH_3), 1.64 (s, 12H, NCCH_3), 2.19 (s, 6H, N(CH_3)$_2$), 2.32 – 2.38 (m, 8H, CH_2CH_3), 2.43 – 2.50 (m, 8H, CH_2CH_3), 4.94 (s, 2H, C*H*), 6.02 (d, $^3J_{HH}$ = 6.0 Hz, 2H, DMAP-Ar*H*), 7.15 (s, 12H, Ar*H*), 7.99 (d, $^3J_{HH}$ = 6.1 Hz, 2H, DMAP-Ar*H*); ^{13}C{^{1}H} NMR (101 MHz, C_6D_6, 298 K) δ 14.5 (CH_2CH_3), 24.1 (NCCH_3), 25.2 (CH_2CH_3), 38.1 (N(CH_3)$_2$), 95.0 (*C*H), 106.1 (DMAP-Ar*C*), 123.9, 125.8, 137.6, 149.1 (Ar*C*), 149.7, 154.5 (DMAP-Ar*C*), 165.9 (N*C*CH_3); IR *v*/cm^{-1} (Nujol): 1510 (s), 1520 (s), 1265 (m), 1226 (s), 1174 (s), 1003 (m), 796 (m), 755 (s); MS (EI, 70 eV): *m*/*z* (%) = 557.5

({(^{Dep}Nacnac)Mg}$_2$-4$CH_2CH_3^+$, 49), 385.3 ((^{Dep}Nacnac)Mg^+, 82), 174.2 ($MeCNDep^+$, 100); anal. calc. for $C_{57}H_{76}Mg_2N_6$: C 76.59 %, H 8.57 %, N 9.40 %: found: C 76.42 %, H 8.63 %, N 9.48 %.

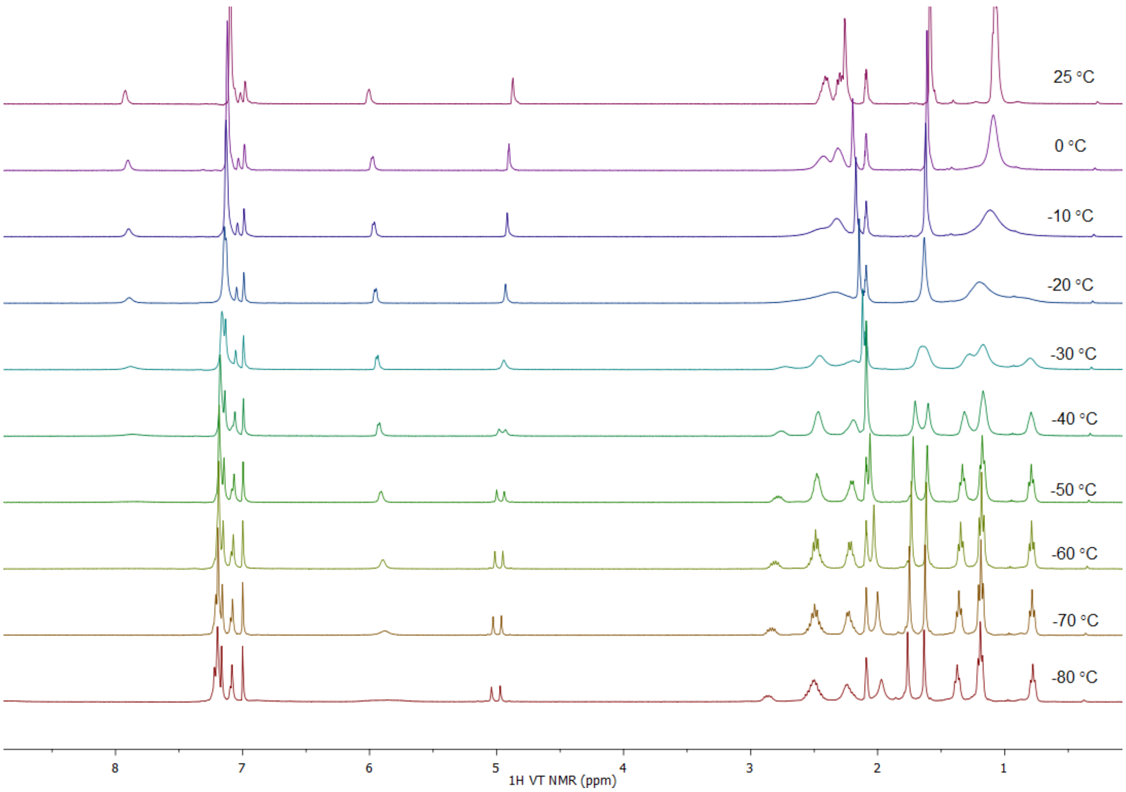

Figure S14. Varaible temperature ^{1}H NMR spectra (400 MHz, toluene-d_8) of **8**.

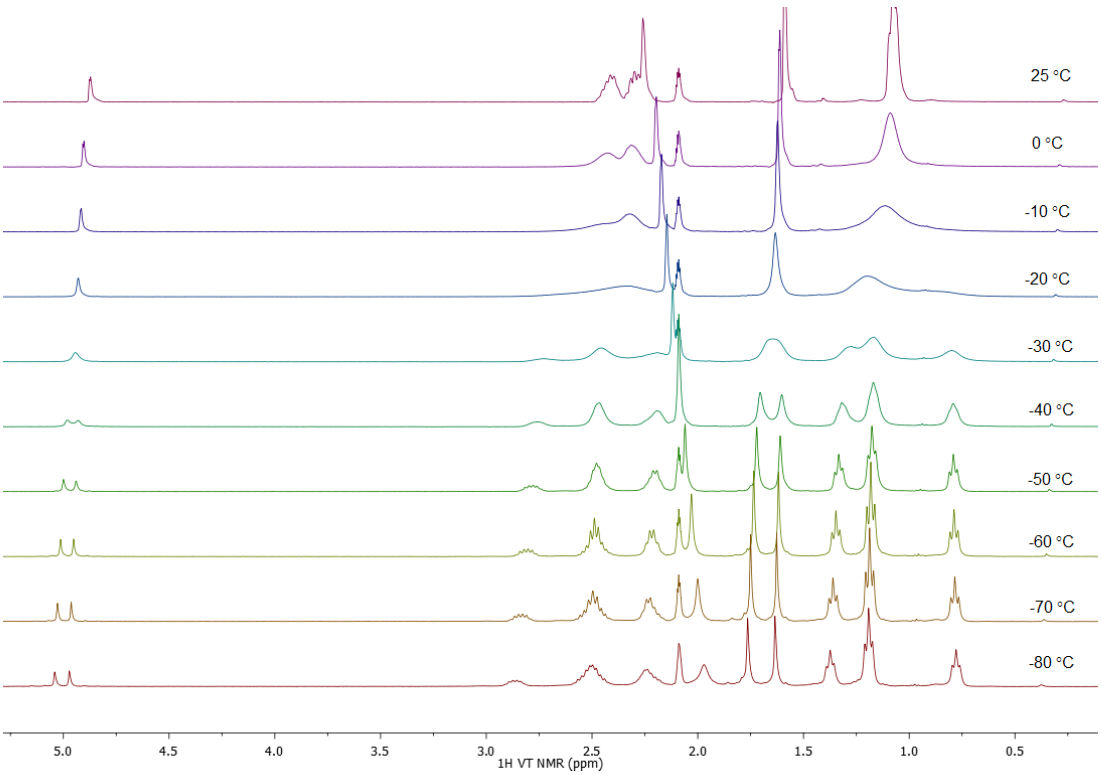

Figure S15. Excerpt of varaible temperature ^{1}H NMR spectra (400 MHz, toluene-d_8) of **8**.

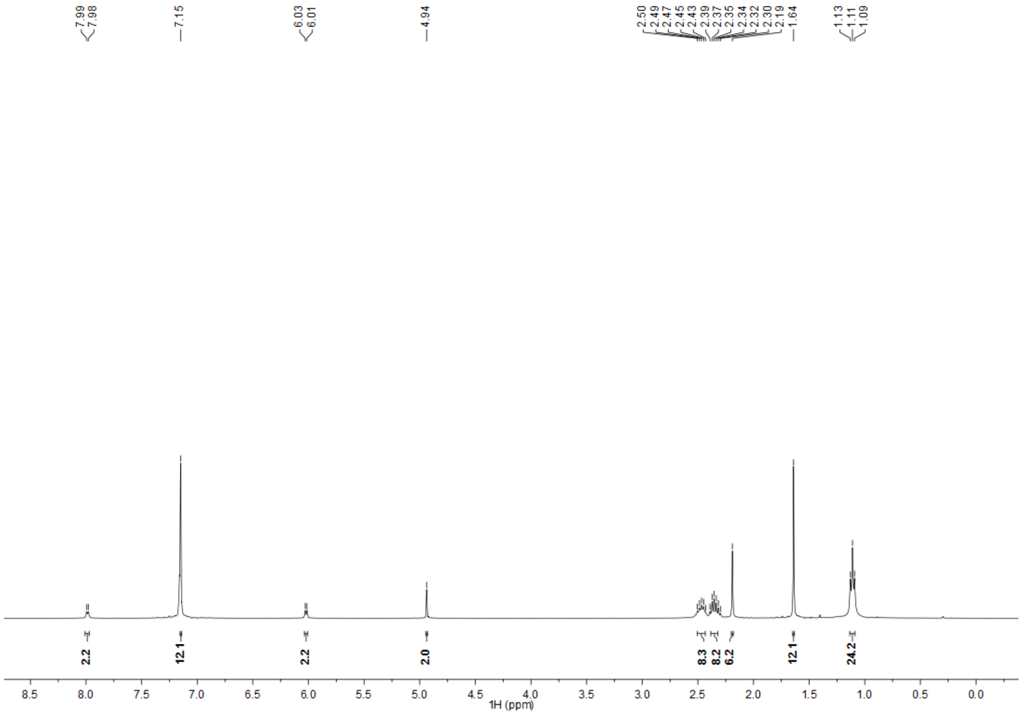

Figure S16. ^{1}H NMR spectrum (400 MHz, 298 K, C_6D_6) of **8**.

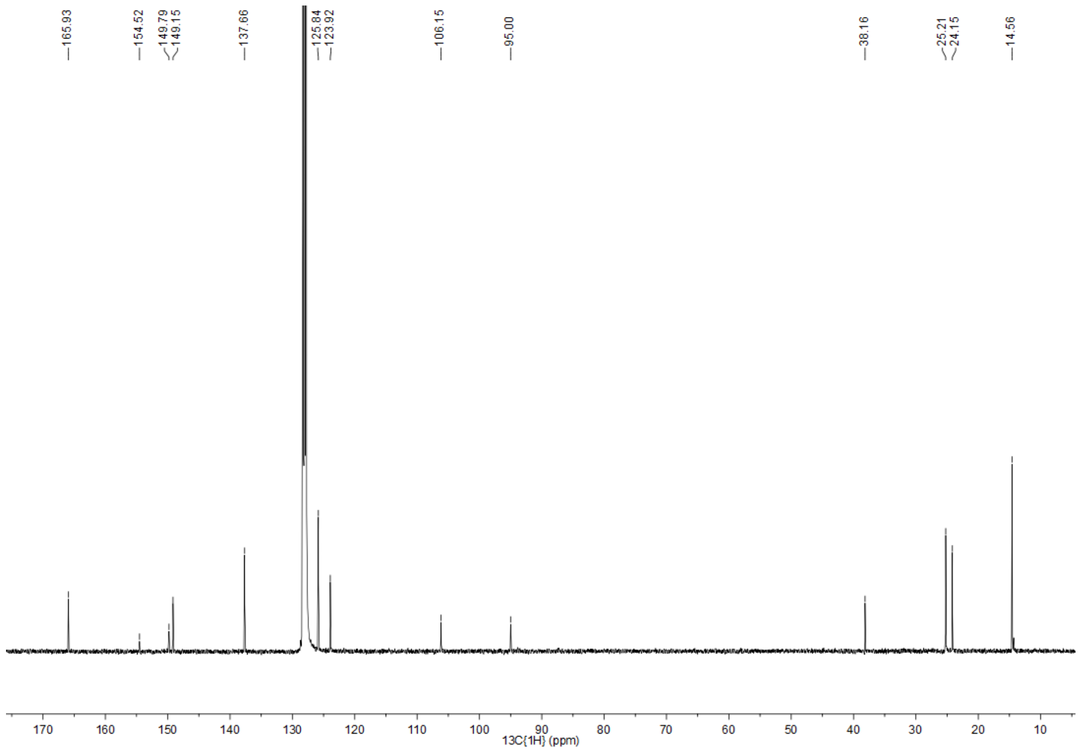

Figure S17. $^{13}C\{^1H\}$ NMR spectrum (101 MHz, 298 K, C_6D_6) of **8**.

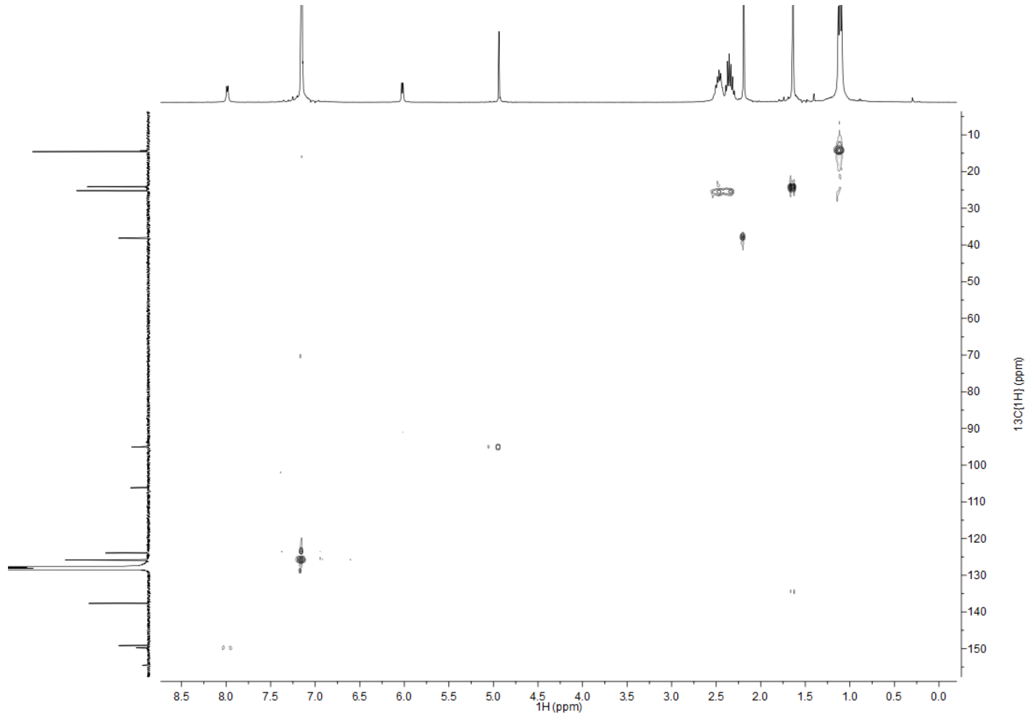

Figure S18. HMQC spectrum (^1H: 400 MHz; ^{13}C: 101 MHz, 298 K, C_6D_6) of **8**.

Synthesis of [{(^{Dep}Nacnac)Mg(DMAP)}$_2$]. [{(^{Dep}Nacnac)Mg}$_2$] (200 mg, 0.259 mmol) and DMAP (63 mg, 0.518 mmol) were dissolved in toluene (8 mL) at room temperature. This resulted in an intense red solution. The mixture was stirred for 1h, filtered, and the filtrate concentrated to *ca.* 4 mL *in vacuo*. The filtrate was then placed at -30 °C for 2 d, after which time dark red crystals of the title compound had deposited. These were isolated and a second crop obtained from the mother liquor (108 mg, 41 %). M.p.145-148 °C; ^{1}H NMR (400 MHz, C_6D_6, 298 K) δ 1.11 (t, $^3J_{HH}$ = 7.6 Hz, 24H, CH$_2$CH_3), 1.68 (s, 12H, NCCH_3), 2.20 (s, 12H, N(CH_3)$_2$), 2.20 – 2.38 (m, 8H, CH_2CH$_3$), 2.47 – 2.55 (m, 8H, CH_2CH$_3$), 4.98 (s, 2H, CH), 6.03 (br, 4H, DMAP-ArH), 7.14-7.18 (m, 12H, ArH), 8.19 (br, 4H, DMAP-ArH); ^{13}C{^{1}H} NMR (101 MHz, C_6D_6, 298 K) δ 14.6 (CH$_2$$CH_3$), 24.3 (NC$CH_3$), 25.2 ($CH_2CH_3$), 38.2 (N($CH_3$)$_2$), 94.8 ($C$H), 106.3 (DMAP-Ar$C$), 123.7, 125.7, 137.8, 149.9 (ArC), 150.1, 154.3 (DMAP-ArC), 165.5 (NCCH$_3$); IR v/cm^{-1} (Nujol): 1608 (s), 1514 (s), 1268 (m), 1225 (m), 1173 (s), 1103 (w), 1002 (s), 927 (w), 799 (m), 760 (m); MS (EI, 70 eV): *m*/*z* (%) = 557.5 ({(^{Dep}Nacnac)Mg}$_2$-4CH$_2$-CH$_3^+$, 43), 385.4 ((^{Dep}Nacnac)Mg$^+$, 86), 174.2 (MeCNDep$^+$, 100); anal. calc. for $C_{64}H_{86}Mg_2N_8$: C 75.66 %, H 8.53 %, N 11.03 %: found: C 75.49 %, H 8.71 %, N 10.90 %.

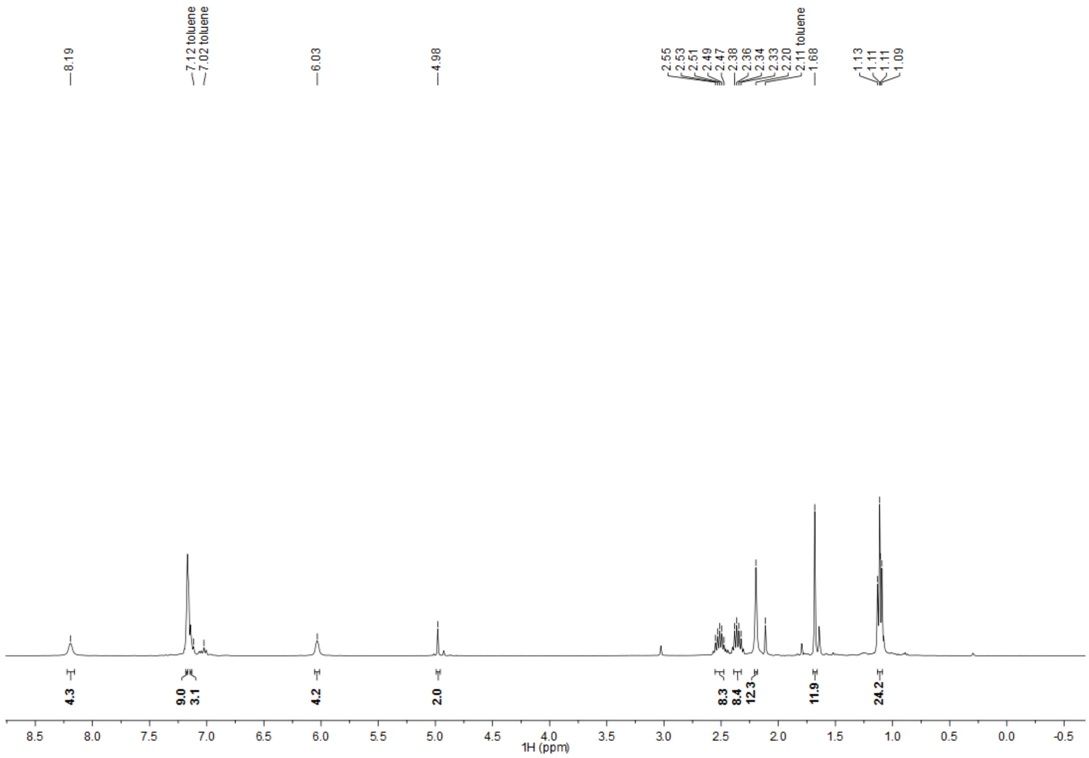

Figure S19. ^{1}H NMR spectrum (400 MHz, 298 K, C_6D_6) of [{(^{Dep}Nacnac)Mg(DMAP)}$_2$].

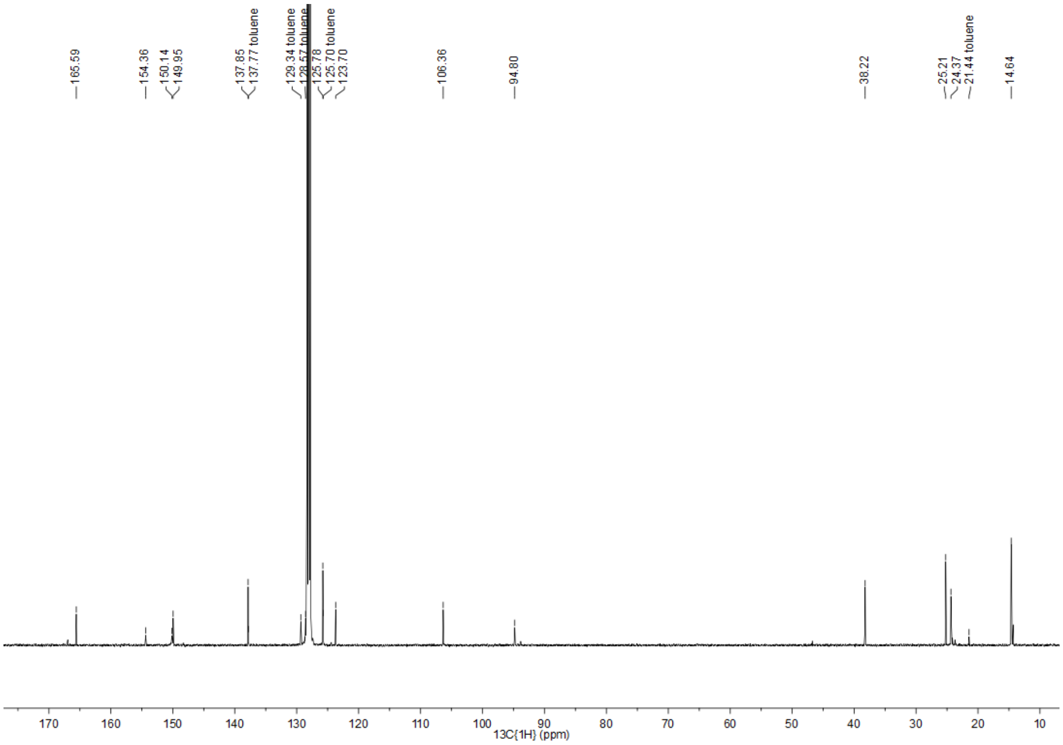

Figure S20. $^{13}C\{^{1}H\}$ NMR spectrum (101 MHz, , 298 K, C_6D_6) of [{(^{Dep}Nacnac)Mg(DMAP)}$_2$].

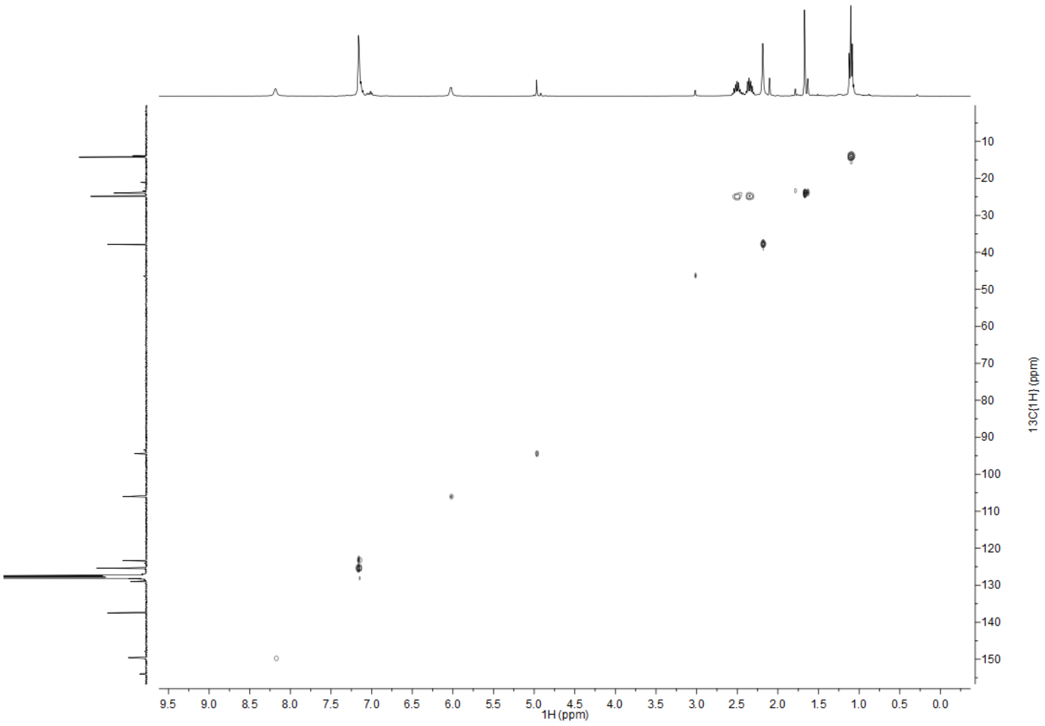

Figure S21. HMQC spectrum (^{1}H: 400 MHz; ^{13}C: 101 MHz, , 298 K, C_6D_6) of [{(^{Dep}Nacnac)Mg(DMAP)}$_2$].

Comments on variable temperature ^{1}H NMR spectroscopic studies of DMAP adduct complexes 6 and 8.

Similar to the previous report on **4**,[5] variable temperature NMR spectroscopic studies of the adducts **6** and **8** revealed fluxional behavior, which is believed to arise from rapid "hopping" of the DMAP ligand between the two Mg centers. This is rapid on the NMR timescale at room temperature, as evidenced by the presence of one set of β-diketiminate signals in their spectra. Cooling d_8-toluene solutions of **6** and **8** leads to their ^{1}H NMR spectra resolving to exhibit two sets of ligand β-diketiminate signals, typically at temperatures below -20 °C.

Synthesis of [{(^{Dep}Nacnac)Mg(μ-C_3O_3)Mg(DMAP)(^{Dep}Nacnac)}$_2$], 9. [{(^{Dep}Nacnac)Mg}$_2$] (150 mg, 0.195 mmol) and DMAP (24 mg, 0.195 mmol) were dissolved in toluene (7 mL) at -78 °C. This resulted in an orange-red solution. The mixture was stirred for 1h, then warmed to room temperature. The orange-red solution was cooled to -78 °C for 30 minutes, then the reaction vessel placed under vacuum, and backfilled with excess CO gas. The solution was stirred for 1h, warmed to room temperature, and left overnight to yield a dark red brown solution. The mixture was then filtered, and the filtrate concentrated to *ca.* 2 mL *in vacuo*, and layered with hexane in a long, thin Schlenk flask. This was then placed at -30 °C for 3 d, after which time colourless crystals of **9** had deposited. These were isolated and a second crop obtained from the mother liquor (36 mg, 19 %). M.p. 213-216 °C; ^{1}H NMR (400 MHz, THF-d_8, 298 K) δ 0.90 (t, $^{3}J_{HH}$ = 7.6 Hz, 24H, CH_2CH_3), 1.11 (t, $^{3}J_{HH}$ = 7.6 Hz, 24H, CH_2CH_3), 1.45 (s, 12H, NCCH_3), 1.67 (s, 12H, NCCH_3), 2.40 (q, $^{3}J_{HH}$ = 7.5 Hz, 8H, CH_2CH$_3$), 2.46 – 2.53 (m, 8H, CH_2CH$_3$), 2.56 (q, $^{3}J_{HH}$ = 7.5 Hz, 16H, CH_2CH$_3$), 2.94 (s, 12H, N(CH_3)$_2$), 4.56 (s, 2H, C*H*), 4.89 (s, 2H, C*H*), 6.48 (d, $^{3}J_{HH}$ = 6.8 Hz, 4H, DMAP-Ar*H*), 6.85 (s, 12H, Ar*H*), 7.03 – 7.09 (m, 12H, Ar*H*), 7.94 (d, $^{3}J_{HH}$ = 5.9 Hz, 4H, DMAP-Ar*H*); ^{13}C{^{1}H} NMR (101 MHz, THF-d_8, 298 K) δ 14.7, 14.8 (CH$_2$*C*H$_3$), 23.7 (NC*C*H$_3$) 24.6, 24.7 (*C*H$_2$CH$_3$), 39.0 (N(*C*H$_3$)$_2$), 93.5, 94.9 (*C*H), 107.3 (DMAP-Ar*C*), 124.1, 125.1, 125.9, 126.4, 137.5, 138.3, 147.5, 148.5 (Ar*C*), 150.0, 155.6 (DMAP-Ar*C*), 166.9, 169.0 (N*C*CH$_3$), C_3O_3 resonance not observed; IR *v*/cm^{-1} (Nujol): 1621 (s), 1526 (m), 1391 (m), 1267 (m), 1228 (w), 1176 (s), 1106 (w), 1011 (vs), 801 (m), 760 (m); MS (EI, 70 eV): *m*/*z* (%) = 362.3 (^{Dep}NacnacH^{+}, 31), 347.3 (^{Dep}NacnacH-CH$_3^{+}$, 36), 333.2 (^{Dep}NacnacH-CH$_2$CH$_3^{+}$, 25),

174.1 ($MeCNDep^+$, 100). Due to persistent contamination with trace amounts of an unknown impurity, a satisfactory reproducible microanalysis could not be obtained.

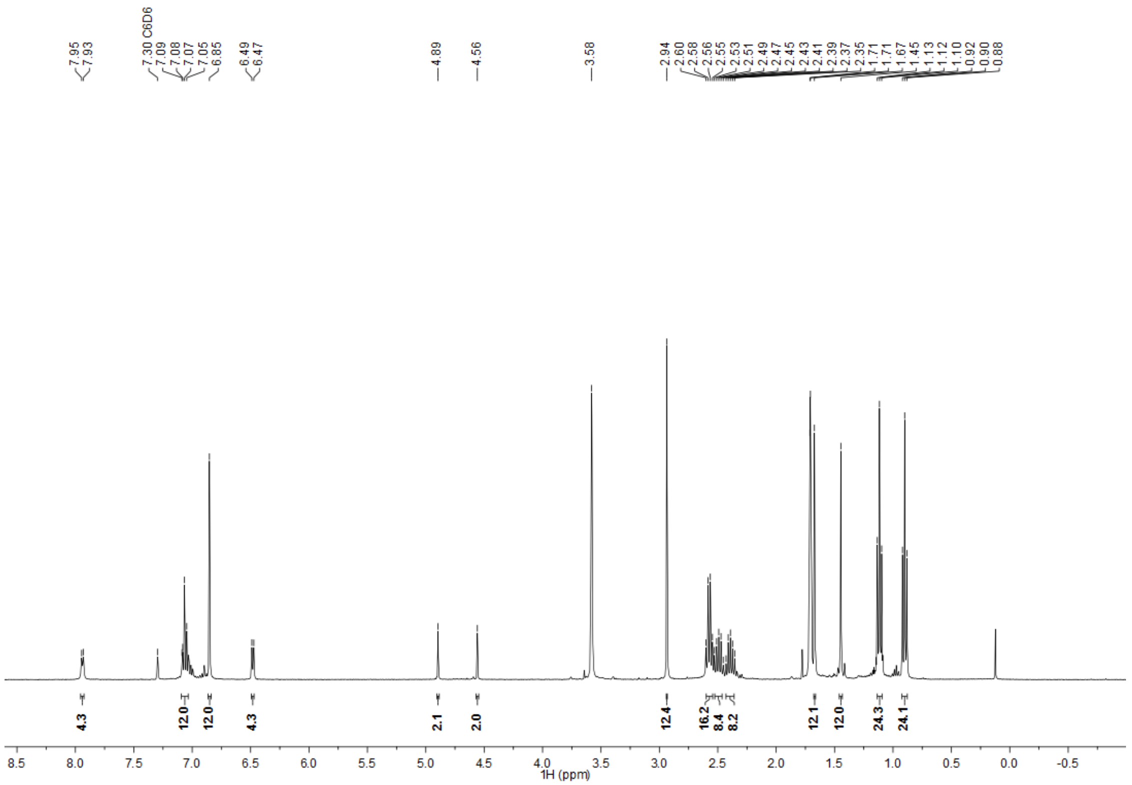

Figure S22. ^1H NMR spectrum (400 MHz, 298 K, THF-d_8) of **9**.

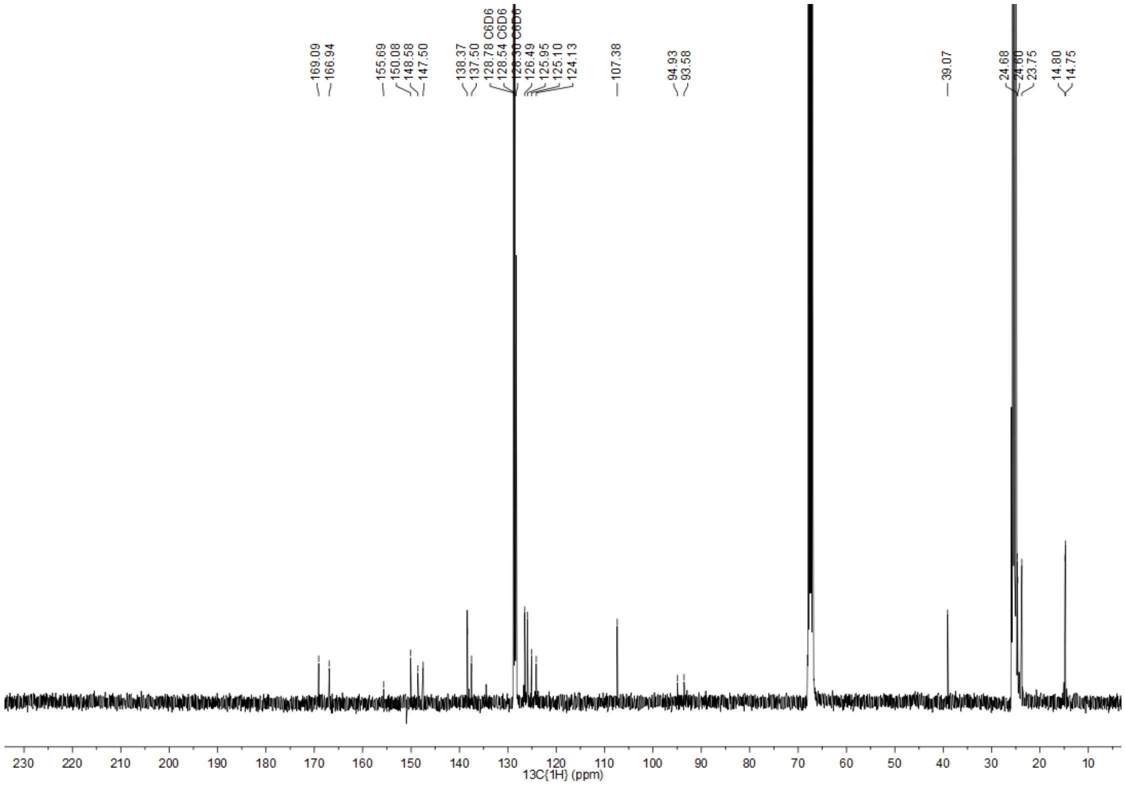

Figure S23. $^{13}C\{^{1}H\}$ NMR spectrum (101 MHz, 298 K, THF-d_8) of **9**.

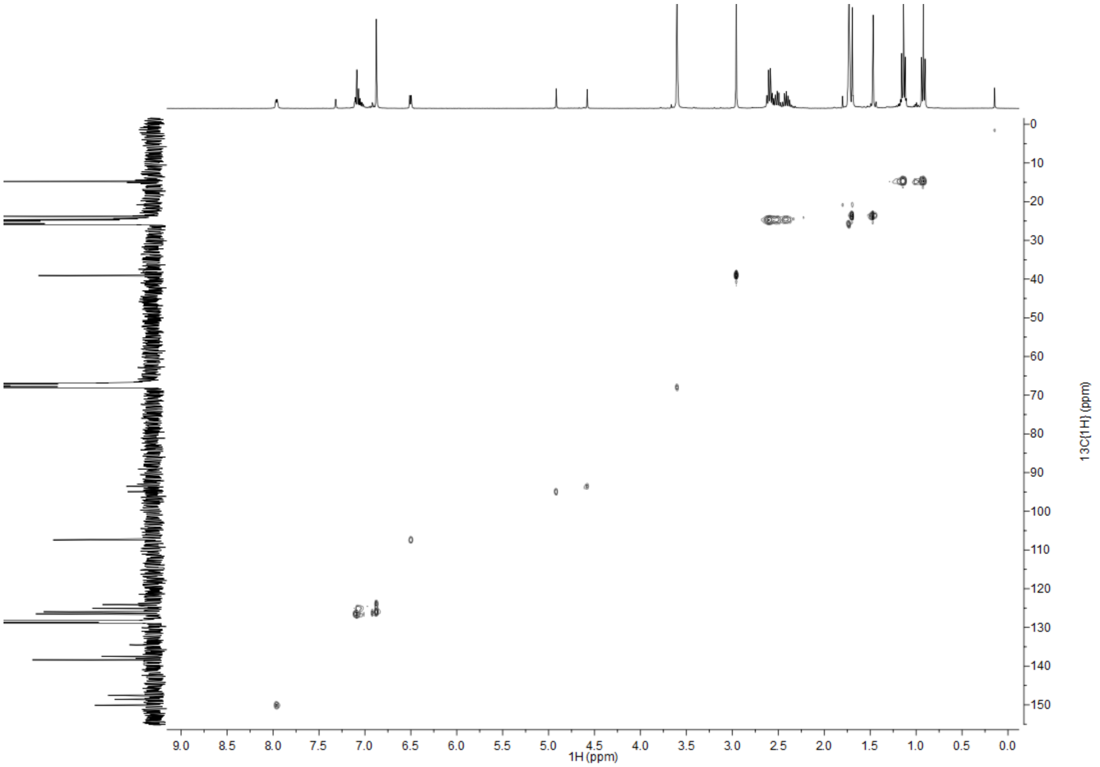

Figure S24. HMQC spectrum (^{1}H: 400 MHz; ^{13}C: 101 MHz, 298 K, THF-d_8) of **9**.

Synthesis of [{(^{Xyl}Nacnac)Mg{μ-OC(H)=C(DMAP^{-H})O}Mg(^{Xyl}Nacnac)}$_2$], 10. [{(^{Xyl}Nacnac)Mg}$_2$] (150 mg, 0.228 mmol) and DMAP (28 mg, 0.228 mmol) were dissolved in toluene (6 mL) at -78 °C. This resulted in an orange-red solution. The mixture was stirred for 1h, then warmed to room temperature. The orange-red solution was cooled down to -78 °C for 30 minutes, then the reaction vessel was placed under vacuum, before being backfilled with excess CO gas. The solution was then stirred for 1h, warmed to room temperature, and stirred overnight, yielding a dark red-brown solution with a colourless solid suspended. The colourless solid was isolated and extracted with hot THF (*ca.* 20 mL), then placed at -30 °C for 2 days, after which time a few colourless crystals of **10** had deposited. The dark red-brown filtrate was concentrated to *ca.* 3 mL *in vacuo,* placed at room temperature for 3 d, after which time colourless **10** deposited. The two crops of the title compound were then combined (71 mg, 37 %). N.B. Once crystallised, compound **10** has neglibible solubility in THF-d_8, so meaningful solution state spectroscopic data could not be obtained. M.p. > 260 °C; IR v/cm^{-1} (Nujol): 1582 (s), 1513 (m), 1279 (m), 1202 (m), 1180 (w), 1094 (w), 1056 (w), 1008 (s), 904 (m), 836 (w), 763 (s); MS (EI, 70 eV): *m*/*z* (%) = 837.6 (M/2+H$^+$, 19), 329.1 ({(^{Xyl}Nacnac)Mg}$^+$, 16), 146.1 (MeCNXyl$^+$, 100); anal. calc. for $C_{102}H_{120}Mg_4N_{12}O_4$: C 73.12 %, H 7.22 %, N 10.03 %: found: C 72.77 %, H 7.36 %, N 9.61 %.

Synthesis of [{(^{Mes}Nacnac)Mg{μ-OC(H)=C(DMAP^{-H})O}Mg(^{Mes}Nacnac)}$_2$], 11. [{(^{Mes}Nacnac)Mg}$_2$] (151 mg, 0.211 mmol) and DMAP (26 mg, 0.211 mmol) were dissolved in toluene (6 mL) at -78 °C. This resulted in an orange-red solution. The mixture was stirred for 1h, then warmed to room temperature. The orange-red solution was cooled down to -78 °C for 30 minutes, then the reaction vessel was placed under vacuum, and backfilled with excess CO gas. The solution was then stirred for 1h, warmed to room temperature, and stirred overnight, yielding a dark purple solution. This was filtered, and the filtrate concentrated to *ca.* 3 mL *in vacuo*, then layered with hexane. After 4 d at room temperature colourless crystals of **11** deposited. These were isolated and a second crop obtained from the mother liquor (78 mg, 41 %). N.B. Compound **11** is only partially soluble in THF-d_8, and when dissolved, spectra unavoidably contain signals resulting from the β-diketimine, ^{Mes}NacnacH. M.p. > 260 °C; ^{1}H NMR (600 MHz, THF-d_8, 298 K) δ 0.84 (s, 12H, NCCH_3), 0.93 (s, 12H, ArCH_3), 1.43 (s, 12H,

NCCH_3), 1.63 (s, 12H, ArCH_3), 1.94 (s, 12H, ArCH_3), 2.12 (s, 24H, ArCH_3), 2.31 (s, 12H, ArCH_3), 2.97 (s, 12H, N(CH_3)$_2$), 4.08 (s, 2H, C*H*), 4.91 (s, 2H, C*H*), 6.00 (d, *J* = 2.6 Hz, 2H, DMAP-Ar*H*), 6.13 (dd, *J* = 6.6, 2.6 Hz, 2H, DMAP-Ar*H*), 6.27 (s, 2H, O*H*C=COC), 6.61 (s, 4H, Ar*H*), 6.62 (s, 4H, Ar*H*), 6.66 (s, 4H, Ar*H*), 6.67 (s, 4H, Ar*H*), 8.26 (d, *J* = 6.5 Hz, 2H, DMAP-Ar*H*); ^{13}C{^{1}H} NMR (151 MHz, THF-d_8, 298 K) δ 18.9, 19.0, 19.2, 19.4, 20.9, 21.6 (Ar*C*H$_3$), 22.9, 24.2 (NC*C*H$_3$), 39.1 (N(*C*H$_3$)$_2$), 87.5 (*C*H), 94.5 (DMAP-Ar*C*), 96.3 (*C*H), 103.4 (DMAP-Ar*C*), 128.2, 129.4, 129.6, 130.1, 130.6, 131.1, 132.1, 133.1, 133.4, 134.0, 137.4 (Ar*C*), 138.4 (OH*C*=COC), 147.4 (DMAP-Ar*C*), 147.9 (Ar*C*), 148.5, 154.8 (DMAP-Ar*C*), 159.9 (OHC=*C*OC), 168.8, 169.3 (N*C*CH$_3$); IR *v*/cm^{-1} (Nujol): 1617 (w), 1576 (w), 1514 (w), 1278 (w), 1227 (m), 1193 (s), 1060 (m), 1005 (s), 904 (w), 852 (s), 798 (m), 728 (s); MS (EI, 70 eV): *m*/*z* (%) = 334.3 (^{Mes}NacnacH$^+$, 21), 160.2 (MeCNMes$^+$, 41); anal. calc. for $C_{110}H_{136}Mg_4N_{12}O_4$: C 73.91 %, H 7.67 %, N 9.40 %: found: C 73.46 %, H 7.98 %, N 9.61 %.

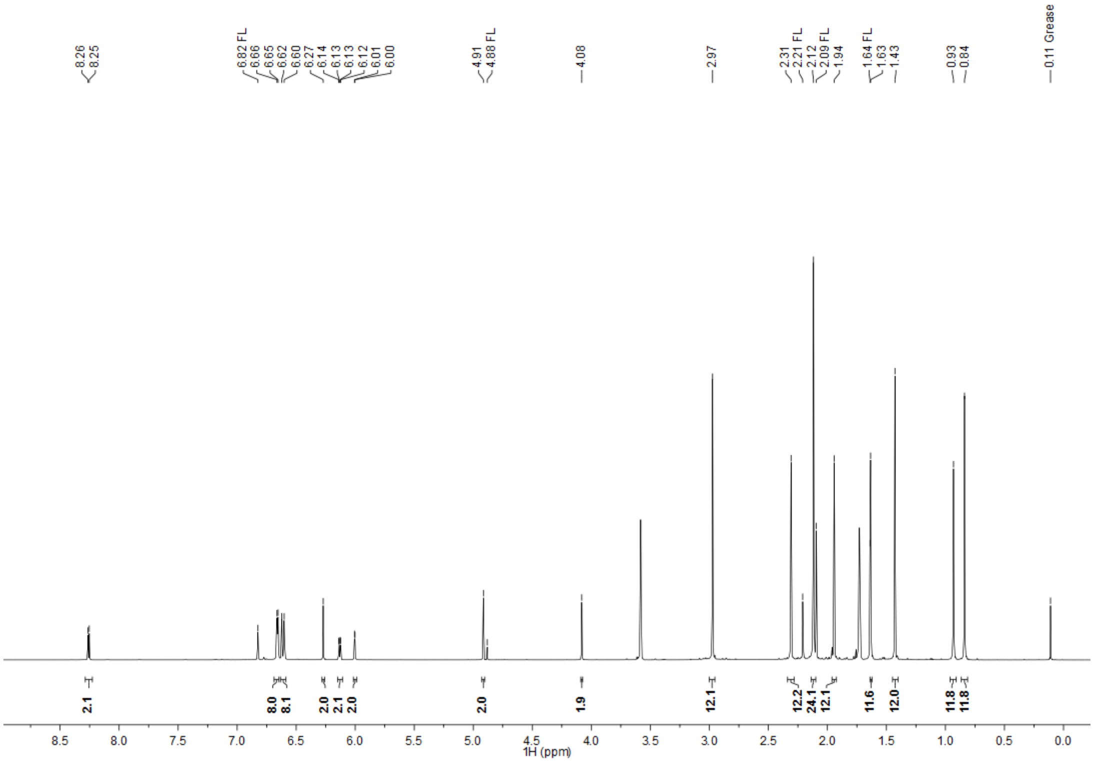

Figure S25. ^{1}H NMR spectrum (600 MHz, 298 K, THF-d_8) of **11** (FL denotes signal arising from co-crystallised ^{Mes}NacnacH).

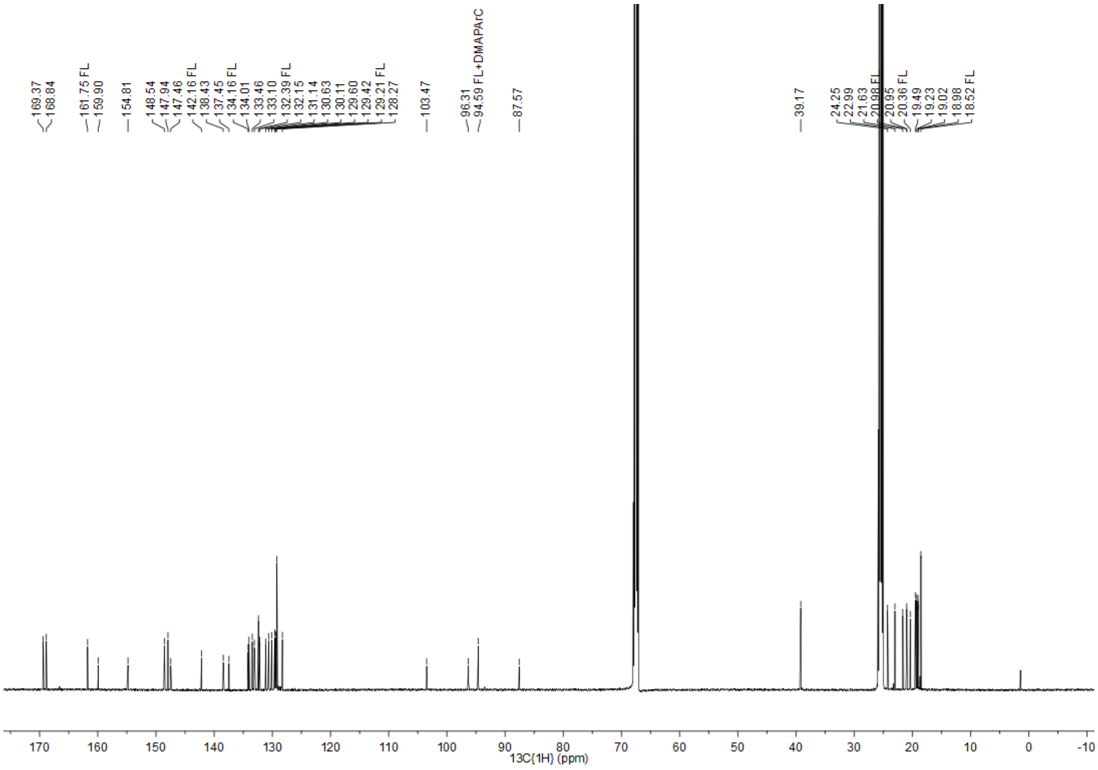

Figure S26. $^{13}C\{^1H\}$ NMR spectrum (151 MHz, 298 K, THF-d_8) of **11** (FL denotes signal arising from co-crystallised ^{Mes}NacnacH).

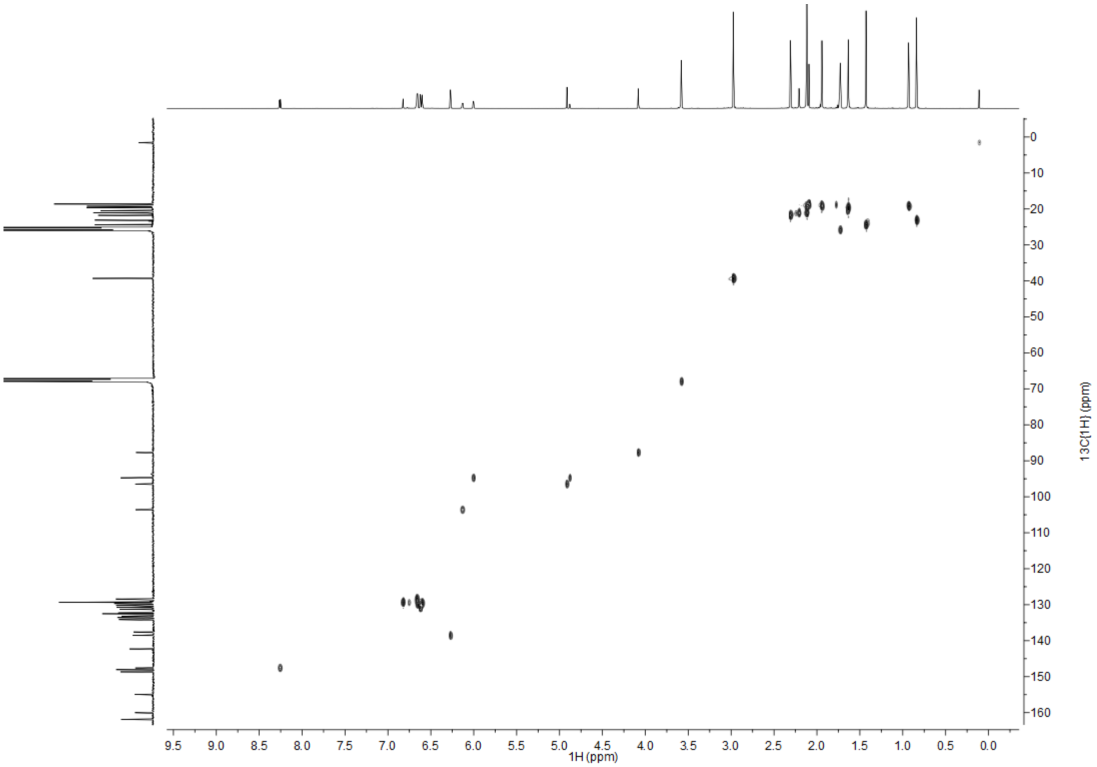

Figure S27. HMQC spectrum (^{1}H: 600 MHz; ^{13}C: 151 MHz, 298 K, THF-d_8) of **11**.

2. X-Ray Crystallographic Studies

Crystals suitable for X-ray structural determination were mounted in silicone oil. Crystallographic measurements were made using either an Rigaku Xtalab Synergy Dualflex diffractometer with a graphite monochromator with Mo Kα radiation (λ = 0.71073 Å) or Cu Kα radiation (1.54180 Å); or the MX2 beamline of the Australian Synchrotron (λ = 0.71090 Å). The software package Blu-Ice[6] was used for synchrotron data acquisition, while the program XDS[7] was employed for synchrotron data reduction. All structures were solved by direct methods and refined on F^2 by full matrix least squares (SHELX-16[8]) using all unique data. Hydrogen atoms are typically included in calculated positions (riding model). Compound **5** co-crystallised with 1.7 % of the bridging iodide compound, [{(^{TCHP}Nacnac)Mg(μ-I)}$_2$], and 6.5 % of the bridging hydroxide compound, [{(^{TCHP}Nacnac)Mg(μ-OH)}$_2$]. Repeated re-crystallisations could not remove these contaminants, as has been found previously in the synthesis of magnesium(I) compounds.[3] Compound **10** crystallised with 4 molecules of heavily disordered THF in the asymmetric unit (8 THFs/molecule of **10**). All attempts to model this disorder were

unsatisfactory. As a result, the SQUEEZE program[9] was used to remove their contribution to the structure factors. The final refinement of the structure included the contribution of the THF molecules to the empirical formula and F(000). The relatively high R1 and wR2values for the crystal structures of **9** and **10** are due to weak diffraction data above θ angles of 23°. Despite this, the molecular connectivities of the compounds are unambiguous, and their presented metrical parameters are reliable within the calculated esd values. Crystal data, details of data collections and refinements for all structures can be found in their CIF files and are summarized in Table S1.

Table S1. Crystal data for **5**, **6**, **8-11**, ^{TCHP}NacnacH **1S**, [(^{TCHP}Nacnac)MgI(OEt$_2$)] **2S**, [{(^{Xyl}Nacnac)Mg(DMAP)}$_2$] **3S** and [{(^{Dep}Nacnac)Mg(DMAP)}$_2$] **4S**.

	**5**·(toluene)$_{4.5}$	**6**	**8**	**9**·(cyclohexyl)$_4$(toluene)	**10**·(THF)$_4$	**11**·(toluene)
empirical formula	$C_{137.50}H_{189.63}I_{0.04}Mg_2N_4O_{0.13}$	$C_{49}H_{60}Mg_2N_6$	$C_{57}H_{76}Mg_2N_6$	$C_{151}H_{208}Mg_4N_{12}O_6$	$C_{134}H_{184}Mg_4N_{12}O_{12}$	$C_{117}H_{144}Mg_4N_{12}O_4$
formula weight	1953.69	781.65	893.85	2384.52	2252.16	1879.67
crystal system	monoclinic	monoclinic	monoclinic	triclinic	monoclinic	triclinic
space group	*P*2$_1$/*n*	*P*2$_1$/*c*	*P*2$_1$/*c*	*P*-1	*P*2$_1$/*c*	*P*-1
a (Å)	18.20670(10)	12.4858(2)	18.7194(2)	17.9190(6)	15.480(3)	15.1058(2)
b (Å)	26.32970(10)	12.0704(2)	11.31290(10)	19.6004(9)	15.114(3)	19.0783(2)
c (Å)	25.8355(2)	31.1338(2)	26.0645(3)	20.9467(7)	27.505(6)	20.0582(2)
α (º)	90	90	90	79.292(3)	90	85.7620(10)
β (º)	105.8060(10)	92.7620(10)	103.7910(10)	75.685(3)	103.08(3)	88.9090(10)
γ (º)	90	90	90	86.860(3)	90	66.9980(10)
V (Å^3)	11916.66(13)	4686.68(13)	5360.57(10)	7004.0(5)	6268(2)	5306.17(11)
Z	4	4	4	2	2	2
T (K)	123(2)	123(2)	123(2)	123(2)	100(2)	123(2)
ρ_{calcd} (g·cm^3)	1.089	1.108	1.108	1.131	1.193	1.176
μ (mm^{-1})	0.623	0.743	0.704	0.687	0.094	0.767
F(000)	4278	1680	1936	2588	2432	2020
reflns collected	121487	45801	51963	137379	71723	100505

unique reflns	22115	8693	10129	26535	11188	19711
R_{int}	0.0580	0.0602	0.0521	0.1650	0.1941	0.0670
R1 [I > 2σ(I)]	0.0679	0.0634	0.0505	0.0900	0.1035	0.0593
wR2 (all data)	0.1816	0.1522	0.1395	0.2550	0.2942	0.1696
largest peak and hole (e·Å^{-3})	1.047, -0.652	0.462, -0.397	0.345, -0.401	1.006, -0.436	0.472, -0.318	0.540, -0.416
CCDC no.	1983489	1983486	1983488	1983492	1983490	1983491

	**1S**·(Et_2O)	**2S**	**3S**·(toluene)$_2$	**4S**·(toluene)$_2$
empirical formula	$C_{57}H_{88}N_2O$	$C_{57}H_{87}IMgN_2O$	$C_{70}H_{86}Mg_2N_8$	$C_{71}H_{94}Mg_2N_8$
formula weight	817.29	967.49	1154.54	1108.16
crystal system	monoclinic	triclinic	monoclinic	monoclinic
space group	*P*2$_1$/*n*	*P*-1	*C*2/*c*	*P*2$_1$/*c*
a (Å)	16.2198(7)	10.4370(2)	18.7699(3)	12.8246(2)
b (Å)	10.7700(6)	14.8235(4)	15.7829(2)	21.5786(3)
c (Å)	28.9971(15)	18.7188(5)	22.8274(4)	24.2161(3)
α (º)	90	84.547(2)	90	90
β (º)	96.100(4)	75.886(2)	109.172(2)	103.4930(10)
γ (º)	90	71.426(2)	90	90
V (Å^3)	5036.7(4)	2661.85(12)	6387.40(19)	6516.52(16)
Z	4	2	4	4

T (K)	123(2)	123(2)	123(2)	123(2)
ρ_{calcd} (g·cm^3)	1.078	1.207	1.131	1.130
μ (mm^{-1})	0.463	0.652	0.084	0.084
F(000)	1808	1032	2344	2400
reflns collected	41363	35036	33068	64489
unique reflns	9509	9893	6275	11777
R_{int}	0.1874	0.0522	0.0154	0.0216
R1 [I > 2σ(I)]	0.0785	0.0375	0.0400	0.0518
wR2 (all data)	0.1626	0.0951	0.1086	0.1362
largest peak and hole (e·Å^{-3})	0.331, -0.257	0.525, -0.755	0.351, -0.243	0.994, -0.409
CCDC no.	1983483	1983484	1983485	1983487

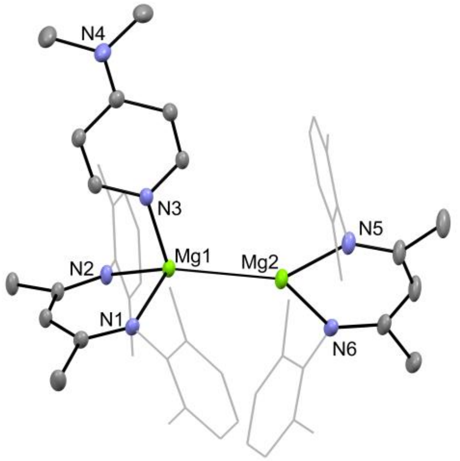

Figure S28. Molecular structure of **6** (25% thermal ellipsoids are shown; hydrogen atoms omitted; aryl substituents shown as wireframe for clarity). Selected bond lengths (Å) and angles (°): Mg(1)-N(3) 2.167(2), Mg(1)-Mg(2) 2.8925(9), N(2)-Mg(1)-N(1) 89.12(7), N(3)-Mg(1)-Mg(2) 115.31(5), N(6)-Mg(2)-N(5) 89.17(8).

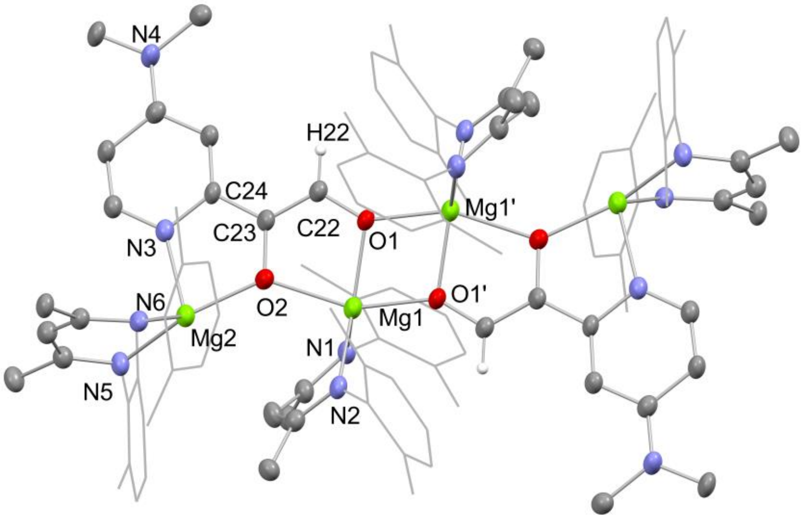

Figure S29. Molecular structure of **10** (25% thermal ellipsoids are shown; hydrogen atoms, except alkenic protons, omitted; aryl substituents shown as wireframe for clarity). Selected bond lengths (Å) and angles (°): Mg(1)-O(1)' 2.016(5), Mg(1)-O(1) 2.020(4), Mg(1)-O(2) 2.110(4), O(1)-C(22) 1.318(7), Mg(2)-O(2) 1.976(4), Mg(2)-N(3) 2.118(5), O(2)-C(23) 1.375(7), C(22)-C(23) 1.364(8), O(1)'-Mg(1)-O(1) 75.24(18), O(1)'-Mg(1)-O(2) 152.0(2), N(5)-Mg(2)-N(6) 91.3(2), O(2)-Mg(2)-N(3) 80.61(19).

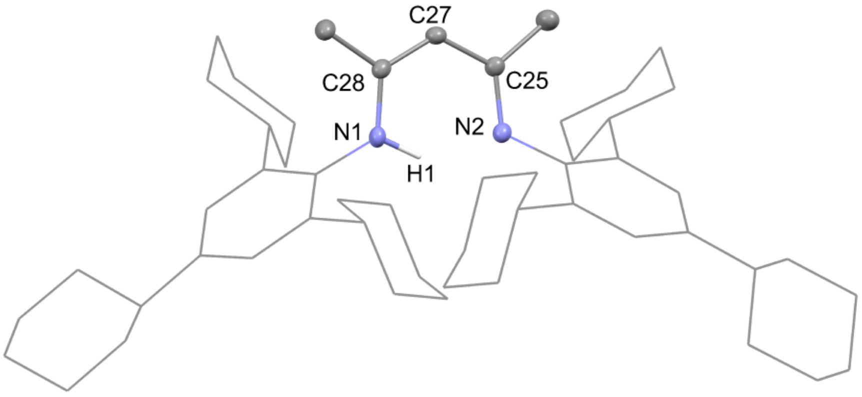

Figure S30. Molecular structure of ^{TCHP}NacnacH **1S** (25% thermal ellipsoids are shown; hydrogen atoms, except amine proton, omitted; aryl substituents shown as wireframe for clarity). Selected bond lengths (Å): N(1)-C(28) 1.346(4), N(2)-C(25) 1.314(4), C(25)-C(27) 1.425(4), C(27)-C(28) 1.375(4).

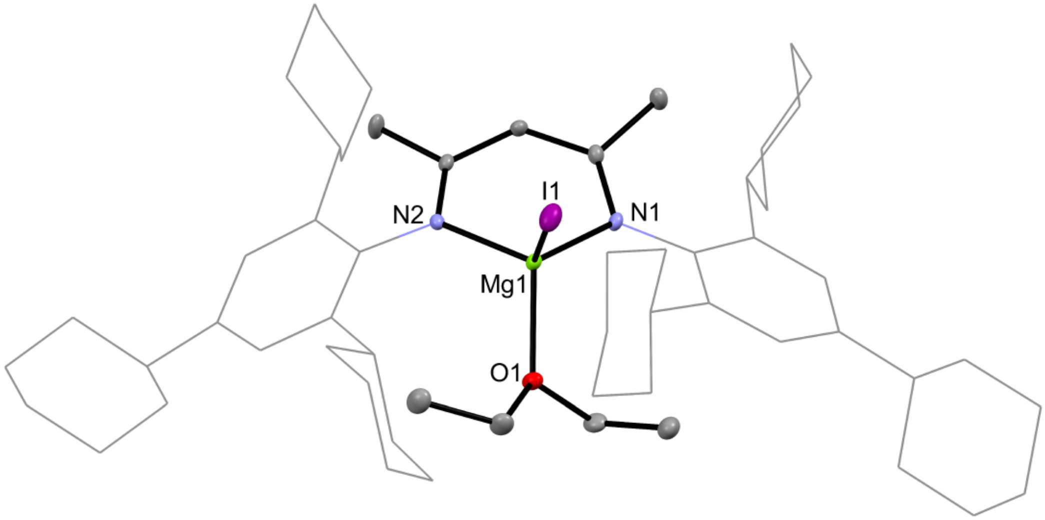

Figure S31. Molecular structure of [(^{TCHP}Nacnac)MgI(OEt$_2$)] **2S** (25% thermal ellipsoids are shown; hydrogen atoms omitted; aryl substituents shown as wireframe for clarity). Selected bond lengths (Å) and angles (°): I(1)-Mg(1) 2.6700(8), Mg(1)-O(1) 2.0431(19), Mg(1)-N(2) 2.048(2), Mg(1)-N(1) 2.054(2), N(2)-Mg(1)-N(1) 96.98(8), O(1)-Mg(1)-I(1) 101.90(6).

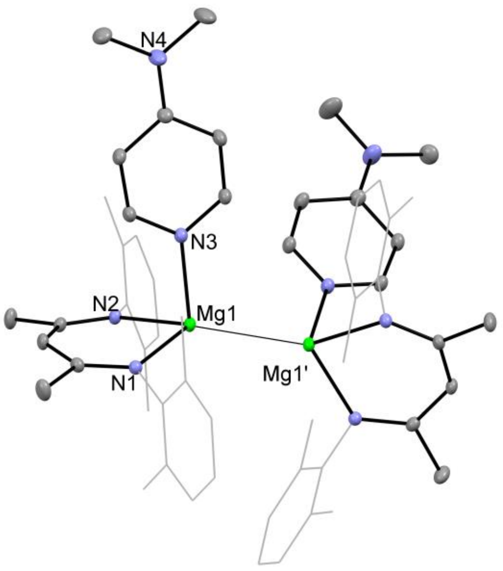

Figure S32. Molecular structure of [{(^{Xyl}Nacnac)Mg(DMAP)}$_2$] **3S** (25% thermal ellipsoids are shown; hydrogen atoms omitted; aryl substituents shown as wireframe for clarity). Selected bond lengths (Å) and angles (°): Mg(1)-N(3) 2.2071(11), Mg(1)-Mg(1)’ 2.9464(7), N(1)-Mg(1)-N(2) 88.31(4), N(3)-Mg(1)-Mg(1)’ 111.28(3).

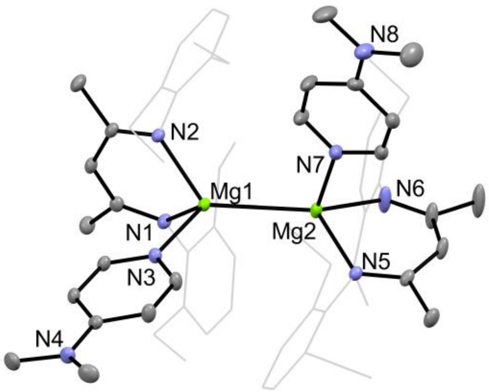

Figure S33. Molecular structure of [{(^{Dep}Nacnac)Mg(DMAP)}$_2$] **4S** (25% thermal ellipsoids are shown; hydrogen atoms omitted; aryl substituents shown as wireframe for clarity). Selected bond lengths (Å) and angles (°): Mg(1)-N(3) 2.2229(14), Mg(1)-Mg(2) 3.0368(7), Mg(2)-N(7) 2.2226(18), N(1)-Mg(1)-N(2) 87.04(5), N(3)-Mg(1)-Mg(2) 109.69(4), N(6)-Mg(2)-N(5) 87.01(6), N(7)-Mg(2)-Mg(1) 113.76(4).

3. Computational Studies

Geometry optimizations were performed using Gaussian09 suite of programs[10] using the Becke's 3-parameter hybrid functional,[11] combined with the non-local correlation functional provided by Perdew/Wang.[12] The 6-311+G(d) all-electron basis set was used for the magnesium atoms and the 6-31G(d,p) for the remaining atoms.[13] All stationary points have been identified for minimum (Nimag=0) or transition states (Nimag=1). Intrinsic Reaction Paths (IRPs)[14] were traced from the various transition structures to obtain the connected intermediates.

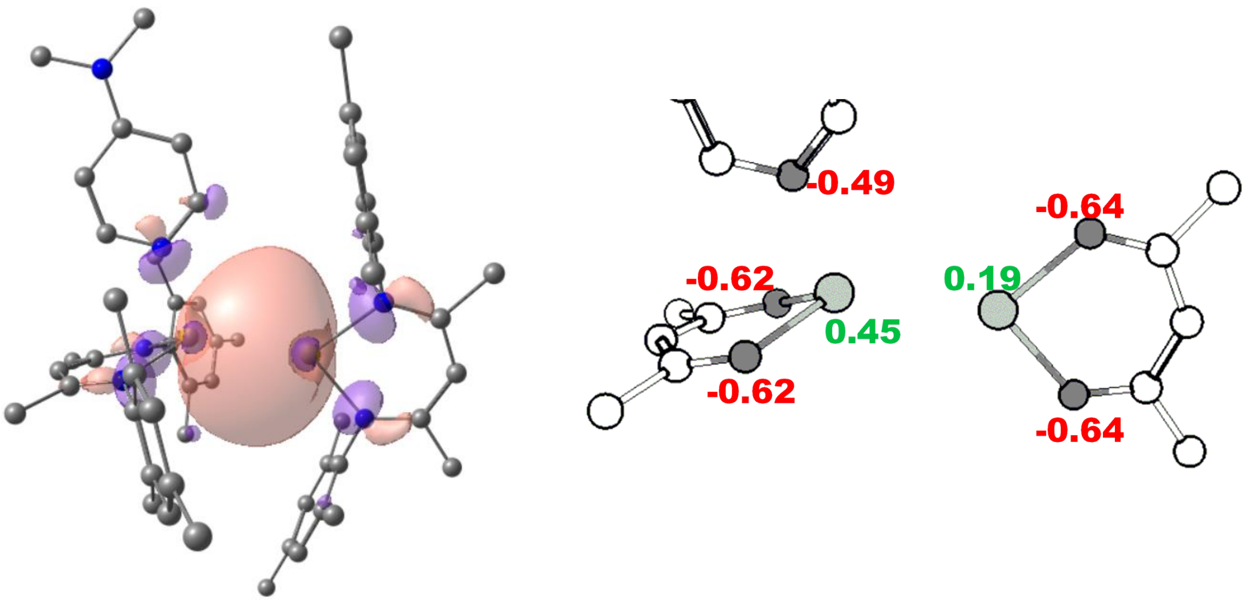

Figure S34. HOMO (left) and NBO charges (right) of **7**.

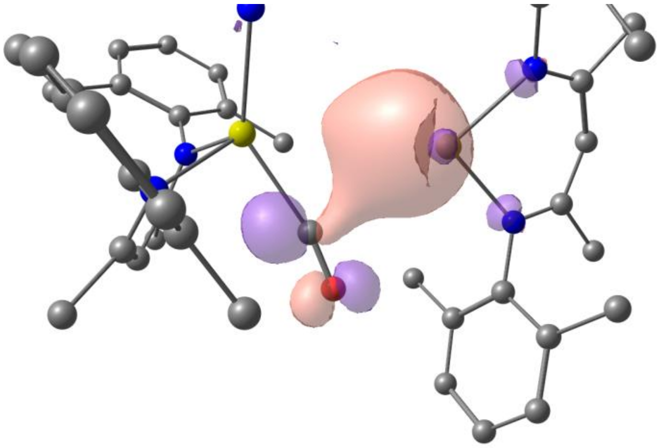

Figure S35. HOMO of TS1.

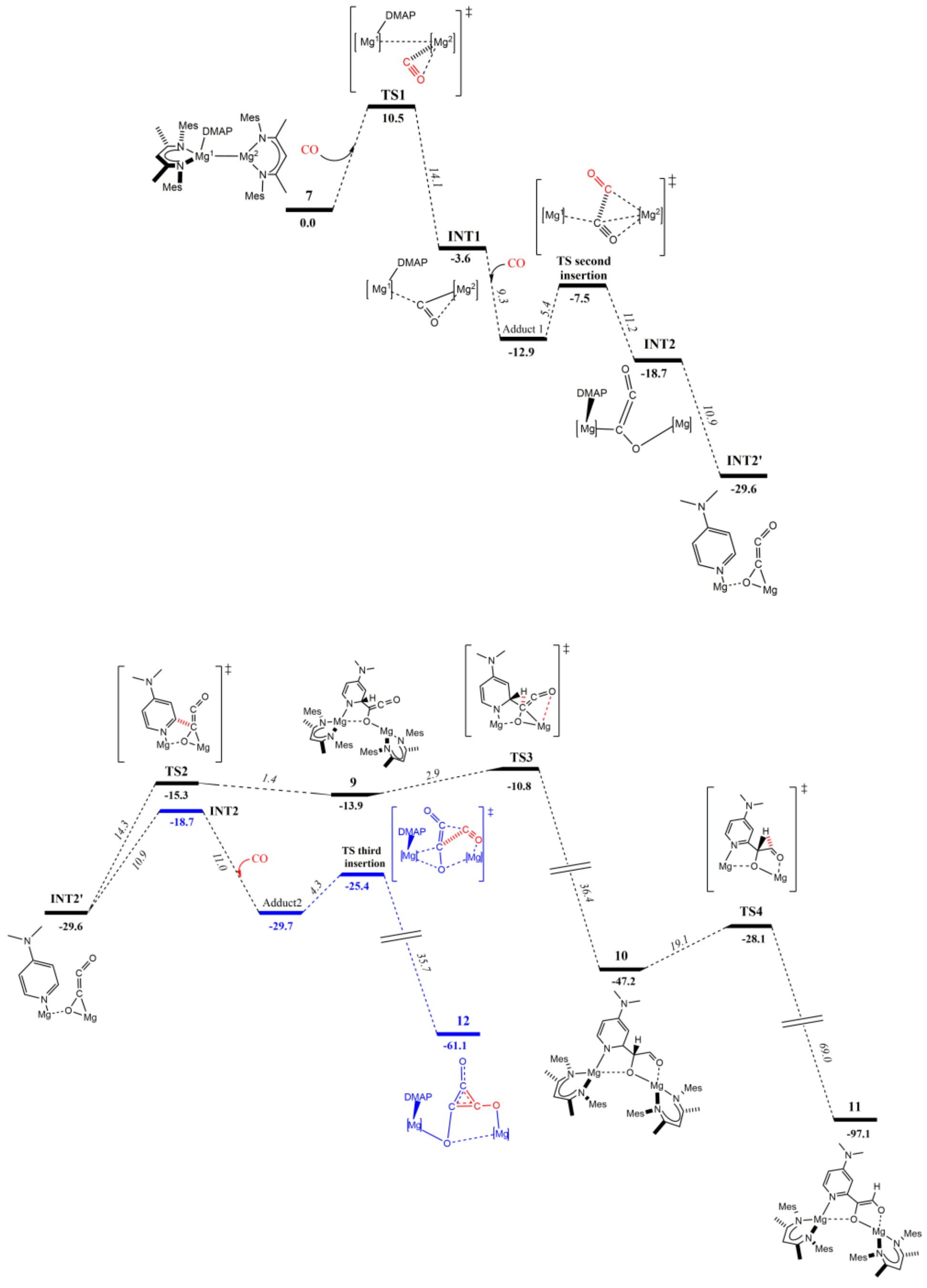

Figure S36. Fully labelled computed (B3PW91) enthalpy profile at 298 K for the formation of ethenediolate complex **11**, or deltate complex **12**, from magnesium(I)-adduct complex **7**, and two or three molecules of CO, respectively.

Table S2. *Cartesian coordinates* of the optimized structures.

129
Complex 7

C	-0.050083	0.740164	-3.274967
C	1.090230	1.067130	-2.511932
C	2.242975	0.262290	-2.599533
C	2.234747	-0.845757	-3.451907
C	1.119043	-1.188863	-4.215889
C	-0.011686	-0.376364	-4.112603
N	1.035925	2.184443	-1.626603
C	1.359530	3.383383	-2.099716
C	1.919077	3.509728	-3.504413
C	3.478907	0.575186	-1.798247
C	1.125002	-2.406344	-5.104350
C	-1.300114	1.571661	-3.177963
Mg	0.366457	1.853085	0.375688
N	2.399918	1.880401	1.322421
C	2.842970	0.939620	2.173360
C	4.122950	0.910211	2.696522
C	5.047990	1.919598	2.344258
C	4.573573	2.910001	1.452295
C	3.275225	2.844943	0.983525
N	6.321945	1.938586	2.835257
C	6.781367	0.865912	3.695069
N	-0.086691	3.932670	0.561136
C	0.437790	4.863652	-0.231422
C	0.210108	6.330768	0.083893
C	-1.017119	4.341951	1.561256
C	-2.373904	4.511464	1.213268
C	-3.289243	4.880056	2.201508
C	-2.909358	5.075747	3.530841
C	-1.563588	4.895968	3.852369
C	-0.611350	4.529195	2.896972
C	-2.836422	4.288280	-0.201587
C	-3.922043	5.442628	4.585009
C	0.827396	4.353455	3.302949
C	7.251747	2.961291	2.398814
C	1.191443	4.597749	-1.395520
Mg	-1.352620	-0.329890	1.379975
N	-3.266835	-1.122348	0.950389
C	-3.961498	-0.526405	-0.143141
C	-3.825177	-1.057108	-1.440332
C	-4.516780	-0.452147	-2.493069
C	-5.322149	0.672309	-2.303597
C	-5.423269	1.191466	-1.010914
C	-4.754798	0.617563	0.072942
C	-2.948050	-2.254785	-1.693690
C	-6.035134	1.324610	-3.460292
C	-4.884326	1.206800	1.452219
C	-3.880384	-2.096590	1.620275
C	-5.275034	-2.526811	1.208679
C	-3.325337	-2.781506	2.718114
C	-2.078539	-2.613207	3.343956

C	-1.796296	-3.534890	4.515122
N	-1.162415	-1.714842	2.976577
C	0.050810	-1.662202	3.717766
C	0.161463	-0.799001	4.828618
C	1.357519	-0.770779	5.547837
C	2.452907	-1.565930	5.198510
C	2.335649	-2.377096	4.068321
C	1.157703	-2.434985	3.315516
C	-0.994168	0.076791	5.232646
C	1.075087	-3.308334	2.092009
C	3.707512	-1.563411	6.034788
H	-3.956255	-3.551868	3.147235
H	-0.902383	-4.142012	4.331802
H	-2.637757	-4.206708	4.700734
H	-1.600813	-2.963352	5.429537
H	-4.420977	-0.875515	-3.492279
H	-5.977071	-1.687308	1.267573
H	-5.644778	-3.332900	1.846981
H	-5.291870	-2.871011	0.168677
H	1.431590	-0.112695	6.412936
H	-1.252136	0.785115	4.434296
H	-0.751116	0.655643	6.129688
H	-1.900565	-0.504977	5.437577
H	-6.045477	2.068603	-0.836572
H	-3.914129	1.557411	1.828757
H	-5.257696	0.476318	2.180081
H	-5.567368	2.062161	1.448609
H	3.180333	-2.994838	3.764686
H	-1.892887	-2.019802	-1.502223
H	-3.030213	-2.583984	-2.734647
H	-3.203935	-3.102435	-1.047435
H	2.014447	-3.847586	1.931249
H	0.870336	-2.709848	1.193974
H	0.267453	-4.046459	2.163649
H	4.000315	-0.546364	6.322757
H	4.547516	-2.021765	5.501005
H	3.568041	-2.130104	6.965045
H	-6.999502	1.744548	-3.152698
H	-6.221920	0.611107	-4.270336
H	-5.443297	2.148435	-3.881625
H	2.907899	3.598742	0.290583
H	5.207508	3.722802	1.117981
H	1.563159	5.477952	-1.909327
H	4.384352	0.104955	3.371283
H	0.979593	4.671861	4.339869
H	1.140896	3.306466	3.222518
H	1.508170	4.930343	2.666256
H	3.134724	-1.456185	-3.521335
H	-4.332910	5.017114	1.920717
H	-1.236865	5.050638	4.880305
H	-0.895480	-0.615958	-4.702713
H	-2.044307	1.243922	-3.910182
H	-1.753410	1.479123	-2.182510
H	-1.104573	2.637955	-3.338778
H	3.330792	0.358635	-0.734166

H	4.327441	-0.023598	-2.146665
H	3.758082	1.632517	-1.863457
H	-2.658222	3.252996	-0.516257
H	-3.908419	4.488953	-0.296662
H	-2.308312	4.926887	-0.919544
H	1.150960	3.257496	-4.245320
H	2.262887	4.527723	-3.704274
H	2.749557	2.815838	-3.671467
H	6.883124	3.966776	2.639763
H	8.204008	2.823528	2.913494
H	7.439111	2.912682	1.316670
H	0.441260	6.551795	1.131540
H	0.821788	6.972443	-0.555488
H	-0.840959	6.603847	-0.066228
H	-4.733815	6.049337	4.168160
H	-4.380984	4.548430	5.028304
H	-3.463503	6.010298	5.402328
H	6.762121	-0.107411	3.184461
H	7.808888	1.067509	4.002056
H	6.168284	0.789669	4.602087
H	2.137488	-2.650428	-5.444616
H	0.737995	-3.289088	-4.577270
H	0.498083	-2.259284	-5.991052
H	2.125510	0.167984	2.443845

131
TS1

C	1.157703	-2.434985	3.315516
C	0.050810	-1.662202	3.717766
C	0.161463	-0.799001	4.828618
C	1.357519	-0.770779	5.547837
C	2.452907	-1.565930	5.198510
C	2.335649	-2.377096	4.068321
N	-1.162415	-1.714842	2.976577
C	-2.078539	-2.613207	3.343956
C	-1.796296	-3.534890	4.515122
C	-0.994168	0.076791	5.232646
C	3.707512	-1.563411	6.034788
C	1.075087	-3.308334	2.092009
C	-3.325337	-2.781506	2.718114
C	-3.880384	-2.096590	1.620275
C	-5.275034	-2.526811	1.208678
N	-3.266835	-1.122348	0.950389
C	-3.961497	-0.526405	-0.143141
C	-3.825176	-1.057108	-1.440332
C	-4.516779	-0.452147	-2.493070
C	-5.322148	0.672309	-2.303598
C	-5.423268	1.191466	-1.010915
C	-4.754797	0.617563	0.072941
C	-2.948049	-2.254785	-1.693690
C	-6.035133	1.324610	-3.460293
C	-4.884326	1.206800	1.452218
Mg	-1.352620	-0.329890	1.379975
Mg	0.366458	1.853085	0.375688

N	-0.339357	3.533596	-0.738813
C	-1.720953	3.876573	-0.655099
C	-2.613087	3.389977	-1.633882
C	-3.971013	3.699597	-1.531420
C	-4.481321	4.467256	-0.482602
C	-3.581058	4.931787	0.476843
C	-2.213501	4.648944	0.414801
C	-2.114586	2.538109	-2.770265
C	-5.955745	4.761195	-0.377363
C	-1.289913	5.181059	1.477595
N	2.052716	1.704756	-0.928799
C	2.456273	2.695934	-1.716656
C	1.766550	3.918054	-1.888512
C	0.444330	4.260798	-1.530626
C	-0.090095	5.544412	-2.139247
C	2.748637	0.460971	-0.990753
C	2.331709	-0.512990	-1.921888
C	2.986515	-1.745765	-1.957072
C	4.039709	-2.052281	-1.093242
C	4.434664	-1.073422	-0.181085
C	3.809162	0.174744	-0.108923
C	1.185145	-0.237405	-2.856137
C	4.281040	1.185877	0.902231
C	4.710323	-3.401558	-1.130467
C	3.722787	2.534611	-2.536502
N	1.418886	2.970087	2.011755
C	1.371704	2.620344	3.308256
C	2.082703	3.258429	4.308376
C	2.914513	4.355468	3.987051
C	2.955525	4.721957	2.621192
C	2.208225	4.014338	1.698964
N	3.634380	5.021866	4.936844
C	4.518539	6.103299	4.549928
C	3.609844	4.567937	6.313187
H	-3.956255	-3.551868	3.147235
H	-0.902383	-4.142012	4.331802
H	-2.637757	-4.206708	4.700734
H	-1.600813	-2.963352	5.429537
H	-4.420975	-0.875515	-3.492279
H	-5.977071	-1.687308	1.267572
H	-5.644778	-3.332900	1.846980
H	-5.291870	-2.871011	0.168676
H	1.431590	-0.112695	6.412936
H	-1.252136	0.785115	4.434296
H	-0.751116	0.655643	6.129688
H	-1.900565	-0.504977	5.437577
H	-6.045476	2.068603	-0.836573
H	-3.914129	1.557411	1.828756
H	-5.257696	0.476318	2.180080
H	-5.567368	2.062161	1.448608
H	3.180333	-2.994838	3.764686
H	-1.892886	-2.019802	-1.502223
H	-3.030212	-2.583984	-2.734647
H	-3.203934	-3.102435	-1.047435
H	2.014447	-3.847586	1.931249

H	0.870336	-2.709848	1.193974
H	0.267453	-4.046459	2.163649
H	4.000315	-0.546364	6.322757
H	4.547516	-2.021765	5.501005
H	3.568041	-2.130104	6.965045
H	-6.999501	1.744548	-3.152699
H	-6.221918	0.611107	-4.270337
H	-5.443296	2.148435	-3.881626
H	2.242833	4.286091	0.646223
H	3.567151	5.546018	2.273167
H	2.268597	4.639384	-2.524562
H	1.980407	2.896140	5.323439
H	-1.821405	5.867898	2.145066
H	-0.874314	4.371700	2.088457
H	-0.434533	5.716302	1.049259
H	5.261234	-1.281822	0.497703
H	-4.650216	3.326534	-2.297172
H	-3.949802	5.540742	1.301783
H	2.661057	-2.488307	-2.684694
H	1.065273	-1.051674	-3.577231
H	0.242772	-0.147130	-2.300485
H	1.316017	0.698343	-3.411410
H	3.554768	1.308441	1.713777
H	5.231269	0.872392	1.348032
H	4.422635	2.178320	0.460475
H	-1.668695	1.607349	-2.399518
H	-2.933971	2.272705	-3.446027
H	-1.339361	3.040837	-3.360580
H	3.580513	1.773247	-3.312808
H	4.000351	3.471507	-3.026039
H	4.561525	2.194691	-1.920012
H	3.968288	6.911152	4.050392
H	4.985968	6.521373	5.442901
H	5.316738	5.761366	3.875788
H	-0.554125	6.181046	-1.378181
H	0.704571	6.108317	-2.634447
H	-0.868261	5.330315	-2.881185
H	-6.422487	4.833044	-1.366212
H	-6.483704	3.970673	0.173147
H	-6.140947	5.702942	0.151154
H	4.008107	3.548623	6.417278
H	4.222374	5.235094	6.921785
H	2.591040	4.581484	6.720889
H	5.746149	-3.345233	-0.777795
H	4.188498	-4.126305	-0.490729
H	4.722054	-3.816147	-2.144801
H	0.726907	1.778215	3.549894
C	0.172941	-0.851944	-0.335933
O	0.089624	-2.212055	-0.634259

131
INT1

C	-0.561176	-1.424080	4.049770
C	-0.463993	-2.520993	3.168996
C	0.720873	-3.280048	3.114699

C	1.776195	-2.950630	3.971143
C	1.698734	-1.880595	4.864193
C	0.521265	-1.128542	4.880653
N	-1.535459	-2.820949	2.281213
C	-2.444491	-3.727590	2.638187
C	-3.501910	-4.161643	1.816674
C	-3.817958	-3.820928	0.485628
C	-4.995970	-4.554508	-0.123916
C	0.853559	-4.420304	2.140259
C	2.851212	-1.530039	5.770423
C	-1.809757	-0.583395	4.093270
N	-3.159527	-2.923860	-0.247960
Mg	-1.619155	-1.763288	0.505101
C	-0.729313	0.109515	0.010507
C	-3.576689	-2.704771	-1.594567
C	-2.997819	-3.455373	-2.636047
C	-3.404540	-3.214491	-3.950740
C	-4.361266	-2.246413	-4.264336
C	-4.896073	-1.494437	-3.215619
C	-4.519357	-1.699094	-1.885673
C	-1.952161	-4.497656	-2.339276
C	-5.105285	-0.851892	-0.787001
C	-4.822160	-2.038366	-5.684446
O	-1.911495	0.260076	0.485335
Mg	0.594469	1.770374	-0.661234
N	0.391890	3.797256	-0.154356
C	0.033253	4.106081	1.194093
C	-1.312870	3.976344	1.601186
C	-1.643697	4.233343	2.933016
C	-0.688194	4.609810	3.879854
C	0.633591	4.731072	3.453904
C	1.014172	4.483798	2.130906
C	-2.382158	3.549541	0.630969
C	-1.078943	4.874236	5.311423
C	2.461851	4.619141	1.737177
C	-2.360177	-4.361219	4.012206
N	2.510216	1.035004	0.074860
C	2.541203	-0.141336	0.731251
C	3.691946	-0.696483	1.258035
C	4.927069	-0.021707	1.120061
C	4.886597	1.211720	0.426038
C	3.682109	1.680009	-0.062161
N	6.088690	-0.527915	1.623222
C	7.335257	0.187231	1.432492
N	0.881660	2.211998	-2.690038
C	0.986020	1.098877	-3.577042
C	2.239175	0.647555	-4.034534
C	2.294459	-0.486614	-4.851037
C	1.151542	-1.196479	-5.216632
C	-0.080581	-0.728859	-4.753123
C	-0.186211	0.403979	-3.944956
C	3.518992	1.348001	-3.657367
C	1.240092	-2.436112	-6.069222
C	-1.537238	0.882427	-3.484434
C	6.089213	-1.819175	2.283696

C	0.797240	3.439962	-3.197840
C	0.618588	4.613028	-2.436201
C	0.386736	4.779913	-1.054978
C	0.072093	6.194205	-0.607200
C	0.860609	3.627816	-4.700961
H	-4.158824	-4.897094	2.267366
H	-1.430548	-4.930462	4.127108
H	-3.200502	-5.036233	4.189776
H	-2.354337	-3.595995	4.796450
H	-2.961477	-3.804868	-4.751946
H	-5.764812	-3.851376	-0.463181
H	-5.447214	-5.244285	0.592962
H	-4.686417	-5.125128	-1.006879
H	0.438323	-0.283400	5.562825
H	-1.971838	-0.058086	3.144140
H	-1.742171	0.170393	4.884136
H	-2.705543	-1.188989	4.275203
H	-5.631956	-0.722122	-3.436139
H	-4.331323	-0.250061	-0.295418
H	-5.576210	-1.459521	-0.004936
H	-5.862320	-0.168950	-1.186108
H	2.681260	-3.557517	3.942543
H	-1.068582	-4.050795	-1.864656
H	-1.624305	-4.994165	-3.258356
H	-2.319057	-5.267884	-1.650220
H	1.837197	-4.894742	2.223380
H	0.729385	-4.072243	1.105961
H	0.092723	-5.193983	2.297369
H	3.603828	-2.326067	5.786849
H	2.516964	-1.367697	6.802034
H	3.349505	-0.606413	5.446903
H	-5.691805	-2.667729	-5.917657
H	-4.036475	-2.293144	-6.404180
H	-5.118282	-0.998851	-5.863156
H	3.644037	2.624907	-0.597647
H	5.780365	1.802151	0.263486
H	0.581284	5.528832	-3.015628
H	3.612600	-1.642325	1.779336
H	3.028598	5.138417	2.517529
H	2.925494	3.636321	1.591009
H	2.589176	5.171550	0.799811
H	3.266743	-0.822977	-5.210371
H	-2.685578	4.137058	3.236909
H	1.398119	5.032133	4.169395
H	-0.991652	-1.260667	-5.022482
H	-2.332347	0.243584	-3.879348
H	-1.618700	0.870597	-2.390297
H	-1.734451	1.914141	-3.801593
H	3.863794	1.033755	-2.664589
H	4.315209	1.108440	-4.370767
H	3.406747	2.436079	-3.623983
H	-2.345916	2.463170	0.463036
H	-3.375704	3.785454	1.028336
H	-2.279228	4.041971	-0.341875
H	0.041783	3.086002	-5.188829

H	0.787538	4.683276	-4.973390
H	1.789398	3.223507	-5.117735
H	7.295481	1.189661	1.878558
H	8.142411	-0.362734	1.918604
H	7.588643	0.292046	0.368278
H	0.732264	6.514762	0.205646
H	0.165216	6.902497	-1.433766
H	-0.950565	6.252242	-0.215874
H	-0.230969	5.248196	5.894920
H	-1.883758	5.616826	5.378656
H	-1.442825	3.962809	5.803318
H	5.763445	-2.621677	1.607753
H	7.101455	-2.049386	2.619519
H	5.432413	-1.821005	3.162895
H	1.147741	-3.346441	-5.461686
H	0.440464	-2.467090	-6.818527
H	2.197916	-2.491857	-6.597777
H	1.587414	-0.659925	0.818451

133
Adduct 1

C	0.704807	-3.112908	3.315895
C	-0.458327	-2.318810	3.290636
C	-0.535479	-1.152275	4.077483
C	0.544894	-0.818729	4.897048
C	1.701498	-1.599840	4.956744
C	1.758913	-2.742482	4.156483
N	-1.526366	-2.659697	2.412629
C	-2.466313	-3.510114	2.827367
C	-2.425893	-4.021262	4.252924
C	-1.761152	-0.278467	4.036801
C	2.857742	-1.202597	5.838567
C	0.816172	-4.336768	2.445296
C	-3.518125	-3.985890	2.023420
C	-3.786428	-3.769612	0.655679
C	-4.965027	-4.532240	0.084702
N	-3.083835	-2.964753	-0.141590
C	-3.444274	-2.889792	-1.521779
C	-2.853968	-3.774375	-2.445142
C	-3.197094	-3.670799	-3.794414
C	-4.102306	-2.713290	-4.256890
C	-4.653117	-1.833074	-3.324120
C	-4.339203	-1.897417	-1.963790
C	-1.857552	-4.808119	-1.990772
C	-4.490015	-2.649847	-5.712089
C	-4.943780	-0.914178	-0.996008
Mg	-1.578430	-1.743910	0.567592
O	-1.844411	0.268199	0.273926
C	-0.598685	0.112887	0.095373
C	-0.015190	-1.452704	-1.419943
O	1.097255	-1.759642	-1.575365
Mg	0.747684	1.695106	-0.768086
N	2.643255	0.978358	0.013279
C	2.655062	-0.113603	0.802766

C	3.803385	-0.645738	1.356588
C	5.055661	-0.042012	1.097560
C	5.033173	1.108616	0.272174
C	3.829883	1.564279	-0.228576
N	6.217337	-0.536142	1.612080
C	7.480675	0.102142	1.298329
N	1.016685	2.298901	-2.751996
C	1.137971	3.576957	-3.111353
C	1.091603	4.673207	-2.225861
C	0.726728	4.728263	-0.864350
C	0.509312	6.118691	-0.299318
C	0.907294	1.312602	-3.780725
C	-0.373727	0.944148	-4.240841
C	-0.484304	-0.044961	-5.219277
C	0.634418	-0.686990	-5.754057
C	1.889840	-0.309094	-5.278871
C	2.049046	0.671355	-4.295674
C	-1.608288	1.600408	-3.682465
C	3.428945	1.011532	-3.797964
C	0.485467	-1.773088	-6.788030
C	1.292427	3.923408	-4.579492
N	0.520686	3.665843	-0.087739
C	0.019716	3.877792	1.233010
C	-1.372721	3.854628	1.458375
C	-1.850292	4.029229	2.759300
C	-0.995915	4.222087	3.846635
C	0.377233	4.228481	3.599812
C	0.902810	4.058034	2.315850
C	-2.337649	3.625388	0.325273
C	-1.541712	4.447141	5.233522
C	2.394974	4.070276	2.111151
C	6.195410	-1.737637	2.424337
H	-4.208556	-4.655932	2.523123
H	-1.506206	-4.586088	4.443039
H	-3.278603	-4.669945	4.465916
H	-2.432536	-3.189628	4.966588
H	-2.739863	-4.359086	-4.504253
H	-5.690170	-3.850295	-0.372929
H	-5.471948	-5.113777	0.858064
H	-4.641908	-5.217129	-0.707582
H	0.477226	0.080984	5.507101
H	-1.909881	0.158994	3.042151
H	-1.672295	0.546662	4.749875
H	-2.672947	-0.839464	4.274866
H	-5.351651	-1.068346	-3.661675
H	-4.176391	-0.261647	-0.562457
H	-5.445476	-1.414102	-0.158761
H	-5.680503	-0.279499	-1.499430
H	2.647076	-3.373487	4.190059
H	-0.963864	-4.337558	-1.561938
H	-1.534302	-5.432162	-2.830242
H	-2.265988	-5.467921	-1.216008
H	1.783358	-4.830765	2.586194
H	0.719750	-4.079370	1.382682
H	0.030178	-5.071437	2.656831

H	3.527938	-2.048607	6.026948
H	2.513065	-0.827729	6.809140
H	3.454462	-0.402235	5.380098
H	-3.649082	-2.910951	-6.364288
H	-4.835693	-1.648782	-5.992074
H	-5.305058	-3.350367	-5.939804
H	3.803194	2.448218	-0.860774
H	5.940202	1.644487	0.019712
H	1.237642	5.640013	-2.694734
H	3.710267	-1.521799	1.986434
H	2.910910	4.318593	3.044734
H	2.759514	3.093620	1.775172
H	2.704165	4.798182	1.351625
H	2.778831	-0.794465	-5.680169
H	-2.927029	4.013654	2.924208
H	1.066899	4.374738	4.430588
H	-1.477514	-0.326292	-5.565482
H	-2.506063	1.225915	-4.184028
H	-1.717538	1.390542	-2.610948
H	-1.584118	2.691403	-3.791959
H	3.587749	0.620349	-2.786048
H	4.194179	0.571267	-4.446133
H	3.603196	2.092516	-3.753142
H	-2.332848	2.570291	0.019954
H	-3.359117	3.873339	0.633696
H	-2.092129	4.227186	-0.556307
H	0.389255	3.643606	-5.134644
H	1.462823	4.993614	-4.718555
H	2.119606	3.372160	-5.038563
H	7.503302	1.144355	1.643723
H	8.287569	-0.433906	1.800135
H	7.685876	0.091150	0.218985
H	1.056511	6.266403	0.637224
H	0.817011	6.887874	-1.011600
H	-0.551247	6.272117	-0.065748
H	-1.835499	5.495051	5.383182
H	-2.431035	3.834417	5.420216
H	-0.798740	4.205553	6.001467
H	5.802439	-2.597844	1.865572
H	7.212277	-1.975162	2.740459
H	5.583269	-1.602990	3.325374
H	1.407768	-1.909089	-7.363323
H	0.246633	-2.737310	-6.319690
H	-0.321953	-1.547951	-7.494300
H	1.683889	-0.569105	0.990842

133
TS second inserion

C	0.584416	-2.725533	3.078801
C	-0.624519	-2.008782	3.192555
C	-0.706899	-0.884308	4.032949
C	0.418305	-0.509152	4.772924
C	1.620210	-1.213174	4.698744
C	1.678258	-2.318880	3.845926

N	-1.752039	-2.389030	2.403899
C	-2.582121	-3.325573	2.875020
C	-2.353523	-3.876430	4.267080
C	-1.981162	-0.087568	4.128718
C	2.828158	-0.782949	5.491025
C	0.714414	-3.887792	2.128712
C	-3.670264	-3.864771	2.164895
C	-4.080051	-3.654079	0.831918
C	-5.241886	-4.493938	0.345457
N	-3.509031	-2.786409	-0.001814
C	-3.969611	-2.709633	-1.351903
C	-3.381570	-3.525536	-2.336911
C	-3.820507	-3.407083	-3.657525
C	-4.813031	-2.499052	-4.031053
C	-5.356495	-1.681369	-3.038478
C	-4.948908	-1.763273	-1.705319
C	-2.275468	-4.487107	-1.987502
C	-5.294352	-2.416335	-5.456775
C	-5.526441	-0.829872	-0.674124
Mg	-2.029510	-1.508423	0.584987
O	-1.699324	0.058103	-0.450884
C	-0.358845	-0.304848	-0.433805
C	0.339805	-1.328232	-0.852270
O	1.191774	-2.161088	-1.061714
Mg	0.985508	1.398913	-0.591554
N	2.946346	0.895543	0.184419
C	3.227019	-0.250488	0.831482
C	4.488019	-0.583609	1.288029
C	5.570019	0.304035	1.081928
C	5.264215	1.509261	0.404350
C	3.970233	1.747410	-0.012848
N	6.831810	0.017026	1.509434
C	7.915575	0.944902	1.249232
N	1.314560	2.060519	-2.540695
C	1.487943	3.344906	-2.832772
C	1.419280	4.397554	-1.891182
C	0.917278	4.404390	-0.573497
C	0.668157	5.777730	0.020561
C	1.229580	1.114850	-3.610644
C	-0.034160	0.793395	-4.145877
C	-0.110822	-0.159461	-5.164204
C	1.019121	-0.810950	-5.661222
C	2.256679	-0.477931	-5.110645
C	2.383741	0.466315	-4.088829
C	-1.288097	1.442664	-3.622951
C	3.742317	0.762643	-3.510182
C	0.901344	-1.862680	-6.734047
C	1.719828	3.762108	-4.271492
N	0.612143	3.318263	0.141040
C	-0.052968	3.508025	1.392118
C	-1.463713	3.525348	1.427403
C	-2.106274	3.712482	2.653411
C	-1.399898	3.876287	3.847577
C	-0.007249	3.827996	3.789489
C	0.680589	3.643171	2.586086

C	-2.265893	3.321063	0.170824
C	-2.120609	4.126111	5.147741
C	2.185861	3.593666	2.586176
C	7.102848	-1.244650	2.171788
H	-4.254323	-4.596358	2.711549
H	-1.389966	-4.395374	4.327794
H	-3.141473	-4.578562	4.548808
H	-2.318726	-3.070400	5.008450
H	-3.365108	-4.040695	-4.417438
H	-6.043436	-3.861099	-0.051142
H	-5.647898	-5.113015	1.148660
H	-4.930856	-5.150072	-0.475633
H	0.348120	0.360856	5.423586
H	-2.173497	0.469935	3.202444
H	-1.919676	0.648141	4.936022
H	-2.854782	-0.724304	4.309089
H	-6.117265	-0.949808	-3.307943
H	-4.764593	-0.125083	-0.315082
H	-5.911142	-1.362387	0.203607
H	-6.345777	-0.240833	-1.098728
H	2.604844	-2.888136	3.773962
H	-1.357730	-3.955692	-1.702885
H	-2.030162	-5.123163	-2.844073
H	-2.537439	-5.138360	-1.145782
H	1.625701	-4.458490	2.337900
H	0.776912	-3.541413	1.088042
H	-0.136167	-4.574911	2.188047
H	3.354947	-1.643251	5.920937
H	2.550764	-0.114628	6.312896
H	3.546799	-0.243625	4.859252
H	-4.498630	-2.674215	-6.164236
H	-5.650281	-1.409767	-5.702206
H	-6.127469	-3.108965	-5.638677
H	3.728141	2.668560	-0.537583
H	6.024095	2.253199	0.197692
H	1.631950	5.380288	-2.297547
H	4.620276	-1.531975	1.793561
H	2.582843	3.865993	3.569977
H	2.550945	2.589126	2.344226
H	2.622299	4.273001	1.845051
H	3.154963	-0.971098	-5.480487
H	-3.195465	3.736141	2.671425
H	0.568643	3.945546	4.706858
H	-1.089851	-0.402242	-5.575111
H	-2.137712	1.217609	-4.276408
H	-1.538023	1.071837	-2.619225
H	-1.192202	2.532381	-3.553192
H	3.826772	0.379447	-2.486651
H	4.528811	0.289439	-4.107669
H	3.952382	1.837637	-3.464847
H	-2.164848	2.285133	-0.183727
H	-3.328149	3.516652	0.354363
H	-1.934820	3.977705	-0.641794
H	0.805366	3.611341	-4.858210
H	1.997427	4.816869	-4.338396

H	2.497413	3.157925	-4.748848
H	7.728960	1.922597	1.712759
H	8.838781	0.545230	1.671234
H	8.072280	1.096554	0.172456
H	1.106918	5.875739	1.018735
H	1.072066	6.564463	-0.620905
H	-0.408527	5.950853	0.136976
H	-2.430944	5.175989	5.237539
H	-3.027395	3.515772	5.229932
H	-1.483471	3.901085	6.010002
H	6.877229	-2.101328	1.522407
H	8.160185	-1.290539	2.436498
H	6.518643	-1.347548	3.095682
H	1.813809	-1.926465	-7.337463
H	0.729171	-2.856332	-6.298693
H	0.063931	-1.655183	-7.409665
H	2.394928	-0.931146	0.979853

133
INT2

C	0.084094	-1.023204	4.790467
C	-0.439459	-1.992658	3.914182
C	0.282164	-3.173034	3.647627
C	1.501010	-3.379353	4.297580
C	2.029377	-2.448111	5.194640
C	1.307855	-1.274067	5.416874
N	-1.670973	-1.747026	3.235723
C	-2.795738	-2.264167	3.733228
C	-2.746329	-3.011779	5.049955
C	-0.237488	-4.189995	2.665138
C	3.325784	-2.714032	5.916030
C	-0.649101	0.268741	5.036588
Mg	-1.689251	-0.463187	1.636370
N	-3.615010	-0.924461	1.084259
C	-4.443263	-1.575072	1.902447
C	-5.904439	-1.705024	1.524239
O	-0.081149	0.149012	0.607541
C	-0.698686	1.306142	1.191234
C	-0.340904	2.539075	1.356687
O	-0.232347	3.718901	1.554000
Mg	1.293165	0.498194	-0.713605
N	0.435622	1.897853	-2.111784
C	-0.802849	2.417831	-1.999970
C	-1.339090	3.313721	-2.904423
C	-0.577477	3.734560	-4.019172
C	0.719481	3.177396	-4.131299
C	1.164587	2.287091	-3.174850
N	-1.060828	4.625231	-4.931075
C	-2.375473	5.209596	-4.745520
N	3.185653	1.260022	-0.298377
C	4.204032	1.003279	-1.113783
C	5.535900	1.689823	-0.885555
C	3.429593	2.065542	0.860326
C	3.236188	3.459297	0.827353
C	3.463041	4.198436	1.990597

C	3.861046	3.601194	3.186346
C	4.041824	2.217382	3.193570
C	3.829752	1.436607	2.055864
C	2.769543	4.162388	-0.419665
C	4.015670	-0.056296	2.119378
C	4.055336	4.420151	4.435979
C	4.163564	0.082709	-2.187929
C	3.229460	-0.934823	-2.469710
N	2.034107	-1.055913	-1.887834
C	1.296174	-2.257219	-2.110573
C	1.523390	-3.368825	-1.270552
C	0.789969	-4.538231	-1.476699
C	-0.166936	-4.645610	-2.489727
C	-0.383541	-3.529189	-3.296432
C	0.323379	-2.334099	-3.124333
C	2.538801	-3.297733	-0.160808
C	-0.920892	-5.931787	-2.710096
C	0.034436	-1.159754	-4.022010
C	3.672006	-1.971980	-3.483715
C	-4.118363	-0.404413	-0.144738
C	-4.565483	0.928876	-0.215083
C	-5.020457	1.421103	-1.442379
C	-5.033061	0.639686	-2.598571
C	-4.570445	-0.675110	-2.503373
C	-4.107714	-1.211526	-1.300188
C	-4.543324	1.814348	1.002038
C	-5.508027	1.199800	-3.914999
C	-3.601029	-2.628039	-1.241302
C	-0.220058	5.072248	-6.024442
C	-4.063182	-2.161171	3.125932
H	-4.871408	-2.628385	3.677041
H	-6.357637	-0.717732	1.379549
H	-6.467669	-2.237819	2.293706
H	-6.019807	-2.241255	0.575696
H	1.707077	-0.523739	6.097830
H	-2.095331	-3.890197	4.984908
H	-3.741942	-3.340837	5.356026
H	-2.329706	-2.375091	5.838876
H	-4.570213	-1.306015	-3.391531
H	-2.534342	-2.663532	-0.989079
H	-3.726987	-3.126750	-2.207319
H	-4.121471	-3.223488	-0.482185
H	2.054010	-4.295687	4.094071
H	-0.340953	-3.762088	1.660356
H	-1.227438	-4.572061	2.941926
H	0.443417	-5.044685	2.596743
H	-5.381519	2.448126	-1.489007
H	-0.675445	0.888473	4.128847
H	-0.155507	0.852980	5.819850
H	-1.688929	0.100702	5.341571
H	-5.001721	2.785204	0.786645
H	-3.510030	1.991905	1.329377
H	-5.077622	1.368122	1.849233
H	-4.663327	1.473059	-4.562227
H	-6.116788	2.099114	-3.770909

H	-6.112595	0.471730	-4.468240
H	3.157760	-3.274141	6.846033
H	3.832890	-1.781663	6.186986
H	4.013716	-3.306195	5.302247
H	2.161294	1.857366	-3.253176
H	1.380624	3.436964	-4.949390
H	5.069785	0.041977	-2.782252
H	-2.342098	3.682121	-2.728510
H	-0.598804	-1.462402	-4.862542
H	-0.489455	-0.361803	-3.482718
H	0.949656	-0.715190	-4.428162
H	3.310102	5.276075	1.959177
H	0.976870	-5.392981	-0.827750
H	-1.124834	-3.584764	-4.092602
H	4.353767	1.725090	4.113751
H	4.439296	-0.353050	3.083925
H	3.057903	-0.579009	2.007648
H	4.675614	-0.429482	1.327574
H	1.684245	4.060012	-0.536033
H	2.990324	5.233646	-0.363924
H	3.236675	3.762598	-1.325549
H	2.250702	-2.561030	0.598603
H	2.638989	-4.267731	0.336459
H	3.528812	-2.998629	-0.523506
H	5.984477	1.345299	0.053769
H	6.235748	1.476590	-1.697182
H	5.417194	2.774141	-0.792225
H	0.087810	4.234204	-6.663426
H	-0.779666	5.775522	-6.642878
H	0.684040	5.581693	-5.662949
H	2.906861	-2.136907	-4.249740
H	4.602612	-1.673938	-3.972536
H	3.838037	-2.940547	-2.997179
H	-0.271791	-6.708444	-3.135770
H	-1.323151	-6.328766	-1.770508
H	-1.759636	-5.790807	-3.399813
H	-2.439259	5.782904	-3.810557
H	-2.586385	5.887281	-5.574091
H	-3.157422	4.439624	-4.732712
H	4.465965	5.410463	4.208596
H	3.101485	4.577240	4.956964
H	4.734867	3.926082	5.139151
H	-1.365577	2.112219	-1.120023

133

INT2'

C	-0.320077	0.208434	4.954836
C	-0.357406	-1.004896	4.242002
C	0.673537	-1.951529	4.397245
C	1.700453	-1.686982	5.306290
C	1.745155	-0.506227	6.051597
C	0.731247	0.431639	5.848144
N	-1.432731	-1.262902	3.334280
C	-2.481929	-1.954399	3.796528

C	-2.478164	-2.429102	5.235496
C	0.678618	-3.225517	3.594011
C	2.838002	-0.265005	7.061047
C	-1.388448	1.253933	4.765442
Mg	-1.387263	-0.529399	1.422828
N	-3.284587	-1.122550	0.944426
C	-4.028721	-1.866815	1.763165
C	-5.405939	-2.309375	1.316144
O	0.188317	-0.189869	0.257653
C	-0.447822	1.060562	0.243430
C	-0.328199	2.140849	0.986343
O	-0.527587	3.248183	1.379900
Mg	1.571809	-0.132300	-1.206967
N	0.392437	1.287585	-2.196249
C	-0.860404	1.447311	-1.581746
C	-1.594208	2.664304	-1.796262
C	-0.999019	3.767889	-2.386881
C	0.335624	3.608854	-2.886324
C	0.936550	2.379400	-2.768388
N	-1.668887	4.963629	-2.544584
C	-2.903111	5.161918	-1.814526
N	3.491641	0.415361	-0.611247
C	4.585218	-0.157539	-1.109718
C	5.948725	0.293988	-0.626750
C	3.643899	1.360584	0.452519
C	3.842203	2.730795	0.205003
C	3.932119	3.606321	1.292824
C	3.821325	3.171895	2.611668
C	3.637215	1.804380	2.832513
C	3.549376	0.893281	1.780466
C	3.955238	3.290429	-1.189324
C	3.365888	-0.573844	2.060944
C	3.874002	4.144100	3.761367
C	4.582912	-1.196538	-2.064510
C	3.520418	-1.965659	-2.572951
N	2.227683	-1.776926	-2.287580
C	1.289142	-2.714661	-2.813221
C	0.977656	-3.875389	-2.076644
C	0.067018	-4.792677	-2.606431
C	-0.552684	-4.590563	-3.841842
C	-0.255235	-3.415123	-4.534473
C	0.650730	-2.469793	-4.045051
C	1.623967	-4.129568	-0.740221
C	-1.490742	-5.619745	-4.419071
C	0.945239	-1.219298	-4.830551
C	3.915946	-3.102616	-3.495222
C	-3.855840	-0.682773	-0.288896
C	-4.582787	0.522858	-0.328342
C	-5.111322	0.948170	-1.549453
C	-4.924846	0.226892	-2.730920
C	-4.188516	-0.957399	-2.663694
C	-3.644419	-1.425390	-1.464607
C	-4.775681	1.347240	0.917261
C	-5.467370	0.730930	-4.042809
C	-2.840334	-2.696722	-1.434997

C	-0.961023	6.130536	-3.026609
C	-3.635653	-2.268073	3.054628
H	-4.366558	-2.867404	3.584960
H	-6.059408	-1.445681	1.149383
H	-5.873644	-2.957928	2.060126
H	-5.354779	-2.847706	0.363440
H	0.751859	1.367342	6.404828
H	-1.611104	-3.062078	5.450706
H	-3.385542	-2.991212	5.466196
H	-2.415578	-1.575733	5.920697
H	-4.025907	-1.537266	-3.570964
H	-1.789087	-2.502844	-1.186182
H	-2.848690	-3.188130	-2.411095
H	-3.215120	-3.407215	-0.688720
H	2.489300	-2.427311	5.433410
H	0.708610	-3.017637	2.517170
H	-0.216905	-3.833929	3.767643
H	1.552980	-3.836585	3.839126
H	-5.683530	1.874640	-1.576888
H	-1.301814	1.747967	3.788332
H	-1.304810	2.035670	5.527125
H	-2.396913	0.829251	4.822234
H	-5.406133	2.218890	0.714302
H	-3.814789	1.713103	1.301843
H	-5.241755	0.775992	1.728811
H	-4.729092	1.359518	-4.558137
H	-6.368525	1.337077	-3.899164
H	-5.719554	-0.094339	-4.717613
H	2.573966	-0.685526	8.040784
H	3.021355	0.804925	7.206475
H	3.780764	-0.730422	6.753231
H	1.933810	2.235656	-3.182239
H	0.865617	4.411183	-3.382925
H	5.566087	-1.501816	-2.404200
H	-2.617125	2.690469	-1.442656
H	0.390864	-1.214377	-5.774782
H	0.666273	-0.320793	-4.266619
H	2.012648	-1.126409	-5.066137
H	4.090505	4.665757	1.093926
H	-0.159778	-5.693086	-2.036662
H	-0.736198	-3.226132	-5.493426
H	3.554570	1.432726	3.852459
H	3.350903	-0.763650	3.137567
H	2.416949	-0.936501	1.648139
H	4.161662	-1.182217	1.613555
H	2.992172	3.696961	-1.521771
H	4.684451	4.108245	-1.220710
H	4.258216	2.533173	-1.917602
H	1.437832	-3.304884	-0.041280
H	1.239815	-5.050657	-0.289893
H	2.714098	-4.222318	-0.818441
H	6.048116	0.130172	0.452570
H	6.748931	-0.249590	-1.134122
H	6.094465	1.366920	-0.791159
H	-0.512845	5.939466	-4.008238

H	-1.671226	6.951586	-3.150032
H	-0.164043	6.465272	-2.343243
H	3.389417	-3.035889	-4.453515
H	4.991144	-3.098323	-3.687423
H	3.647300	-4.072844	-3.061793
H	-0.944584	-6.373419	-5.002351
H	-2.036684	-6.153483	-3.633195
H	-2.226259	-5.162935	-5.090207
H	-2.769485	5.007969	-0.732639
H	-3.263408	6.179465	-1.981734
H	-3.682087	4.471433	-2.162653
H	4.393328	5.067496	3.482890
H	2.863643	4.423670	4.088361
H	4.389301	3.715431	4.628643
H	-1.460124	0.534060	-1.614065

133

TS2

C	-0.341807	0.116641	4.959951
C	-0.358548	-1.071085	4.204248
C	0.683432	-2.009149	4.332029
C	1.706921	-1.758955	5.249047
C	1.736087	-0.601421	6.030188
C	0.707146	0.326622	5.858951
N	-1.427800	-1.312692	3.283218
C	-2.487492	-2.001749	3.727631
C	-2.489882	-2.511257	5.153942
C	0.700316	-3.262604	3.497462
C	2.827819	-0.374355	7.043910
C	-1.429859	1.148763	4.812117
Mg	-1.360623	-0.602979	1.371908
N	-3.277049	-1.094334	0.890463
C	-4.031253	-1.846013	1.694848
C	-5.413519	-2.260275	1.238059
O	0.195561	-0.152623	0.276263
C	-0.386934	1.125851	0.159081
C	-0.248979	2.112946	1.037808
O	-0.325014	3.095105	1.686745
Mg	1.603601	-0.133649	-1.215192
N	0.397333	1.268238	-2.125629
C	-0.807542	1.468525	-1.342737
C	-1.519250	2.764002	-1.541815
C	-1.026547	3.743654	-2.351012
C	0.217809	3.507257	-3.054318
C	0.824532	2.292807	-2.898200
N	-1.715489	4.940574	-2.608172
C	-3.024599	5.091082	-2.016851
N	3.516392	0.387562	-0.591707
C	4.611008	-0.196457	-1.071799
C	5.973215	0.281893	-0.612329
C	3.659617	1.390545	0.416908
C	3.805001	2.750225	0.083973
C	3.888280	3.689523	1.117306
C	3.819865	3.328995	2.461972
C	3.681677	1.972344	2.766032

C	3.600675	0.998542	1.770108
C	3.862997	3.221790	-1.345350
C	3.458504	-0.454217	2.136076
C	3.866915	4.368737	3.551415
C	4.608482	-1.265923	-1.992934
C	3.542861	-2.035170	-2.493502
N	2.248930	-1.826947	-2.223978
C	1.304354	-2.764388	-2.738413
C	0.989899	-3.918761	-1.992932
C	0.069740	-4.833650	-2.510837
C	-0.554444	-4.636565	-3.744732
C	-0.253056	-3.467733	-4.446931
C	0.659951	-2.523878	-3.968199
C	1.644249	-4.170139	-0.660041
C	-1.501669	-5.663777	-4.310359
C	0.952945	-1.276956	-4.759263
C	3.932796	-3.188766	-3.397245
C	-3.844809	-0.619193	-0.333228
C	-4.561261	0.592743	-0.343069
C	-5.077347	1.056128	-1.555368
C	-4.889764	0.365585	-2.754579
C	-4.169958	-0.830035	-2.714238
C	-3.637897	-1.335826	-1.525576
C	-4.761277	1.383743	0.923420
C	-5.413102	0.913607	-4.056240
C	-2.849458	-2.617205	-1.526087
C	-0.947746	6.173398	-2.569181
C	-3.642460	-2.284139	2.975757
H	-4.380433	-2.888416	3.490255
H	-6.056728	-1.385528	1.091322
H	-5.888805	-2.920395	1.966783
H	-5.368179	-2.776422	0.272874
H	0.713809	1.243284	6.446734
H	-1.617392	-3.140357	5.358354
H	-3.393274	-3.087591	5.364015
H	-2.440851	-1.675160	5.861158
H	-4.009279	-1.387316	-3.635724
H	-1.786922	-2.438699	-1.314299
H	-2.895118	-3.104757	-2.503070
H	-3.208661	-3.326505	-0.771324
H	2.505361	-2.492325	5.353316
H	0.739386	-3.030760	2.425731
H	-0.194434	-3.877907	3.650108
H	1.574964	-3.876271	3.734402
H	-5.638684	1.989337	-1.561371
H	-1.355020	1.683387	3.856014
H	-1.359228	1.901387	5.603759
H	-2.430561	0.704559	4.853382
H	-5.370214	2.273343	0.734279
H	-3.803232	1.719762	1.341048
H	-5.255146	0.799118	1.708774
H	-4.661793	1.549176	-4.543039
H	-6.309206	1.525069	-3.904336
H	-5.665903	0.111545	-4.758187
H	2.561298	-0.806123	8.018004

H	3.012669	0.693415	7.202613
H	3.770003	-0.837534	6.731520
H	1.737066	2.084542	-3.459471
H	0.593293	4.205891	-3.791916
H	5.591422	-1.584258	-2.321159
H	-2.458791	2.878843	-1.013163
H	0.413684	-1.285021	-5.712148
H	0.651523	-0.378605	-4.205936
H	2.022877	-1.172949	-4.977274
H	4.007037	4.740055	0.854453
H	-0.159466	-5.728758	-1.933598
H	-0.737479	-3.282381	-5.404834
H	3.630754	1.659229	3.807757
H	3.438630	-0.581868	3.221805
H	2.526893	-0.871716	1.735871
H	4.276294	-1.063938	1.732261
H	2.861099	3.473328	-1.716745
H	4.479494	4.123883	-1.429765
H	4.273257	2.462020	-2.016937
H	1.505092	-3.322433	0.021683
H	1.229910	-5.064915	-0.184071
H	2.728564	-4.310163	-0.750725
H	6.081549	0.157141	0.471276
H	6.775407	-0.271398	-1.105929
H	6.106801	1.349904	-0.815528
H	0.019562	6.043779	-3.055527
H	-1.494483	6.961689	-3.100166
H	-0.761590	6.519662	-1.536315
H	3.415835	-3.124674	-4.361142
H	5.009648	-3.199931	-3.579657
H	3.648125	-4.151193	-2.957041
H	-0.964041	-6.419678	-4.898643
H	-2.040608	-6.194946	-3.517947
H	-2.243131	-5.205959	-4.974281
H	-2.998301	5.190561	-0.915554
H	-3.498253	5.989581	-2.427462
H	-3.647354	4.227385	-2.269119
H	4.384639	5.275067	3.219012
H	2.855188	4.665451	3.858713
H	4.381235	3.994403	4.443908
H	-1.496067	0.619797	-1.529177

133
Product TS2

C	16.680621	37.327027	25.239969
C	15.797790	36.232132	25.388961
C	15.024040	36.126461	26.564144
C	15.183509	37.084786	27.572540
C	16.069982	38.150804	27.458147
C	16.806490	38.251594	26.273987
N	15.636825	35.317750	24.311302
C	15.925899	34.025607	24.474351
C	15.465395	33.000882	23.613260
C	14.371430	33.031410	22.713616
C	13.788984	31.693485	22.308542

C	13.998399	35.041295	26.773331
C	16.212774	39.178780	28.550321
C	17.476711	37.507261	23.978680
N	13.836468	34.154399	22.238651
Mg	15.081782	35.794841	22.376084
O	16.496122	36.001852	20.899616
Mg	18.184856	35.210867	20.149465
N	20.028085	34.913345	21.066474
C	20.382195	35.459332	22.339227
C	20.287928	34.642747	23.484985
C	20.624999	35.179549	24.729166
C	21.051754	36.501584	24.875539
C	21.141035	37.286173	23.725432
C	20.808175	36.795463	22.459242
C	19.838291	33.209653	23.372474
C	21.373615	37.066487	26.234527
C	20.919355	37.689075	21.252604
C	12.560729	34.117209	21.602990
C	11.392717	34.047191	22.391330
C	10.147219	34.046160	21.760851
C	10.016989	34.133640	20.373667
C	11.185689	34.231329	19.618719
C	12.454407	34.235054	20.204747
C	11.473404	34.021043	23.894222
C	13.678434	34.373899	19.343377
C	8.659740	34.164586	19.719818
O	14.475433	37.590650	21.841517
C	15.038180	38.118614	20.822582
C	16.144989	37.198132	20.297231
C	17.099480	37.780244	19.355756
N	18.140921	36.972099	18.995518
C	18.975143	37.416013	18.029219
C	18.865747	38.632077	17.406361
C	17.818547	39.516878	17.809443
C	16.953685	39.076326	18.813936
N	17.684993	40.755221	17.243336
C	18.700804	41.260907	16.342378
N	18.332848	33.496245	18.973889
C	17.208803	32.824179	18.399443
C	16.645431	33.278017	17.191284
C	15.554951	32.589412	16.652368
C	14.999764	31.471044	17.275137
C	15.571451	31.045899	18.476460
C	16.661187	31.702516	19.054121
C	17.216768	34.467715	16.466898
C	13.806007	30.762820	16.689213
C	17.239342	31.207402	20.354022
C	16.653704	41.652098	17.728547
C	19.547414	33.046043	18.650079
C	20.753618	33.484888	19.234233
C	20.974440	34.250685	20.396723
C	22.400162	34.261848	20.912295
C	19.686323	31.949898	17.611891
C	16.775330	33.575494	25.645120
H	15.855437	32.011595	23.829846

H	12.802593	31.544197	22.762550
H	14.434337	30.870403	22.625100
H	13.648953	31.642637	21.223475
H	14.577841	36.991820	28.473687
H	17.519970	34.332952	25.906597
H	17.285409	32.638798	25.403620
H	16.161422	33.391974	26.534041
H	11.114226	34.312100	18.534725
H	14.175905	35.337240	19.512978
H	13.414612	34.325942	18.282727
H	14.419872	33.592672	19.538808
H	17.498299	39.083234	26.145481
H	16.832977	37.813198	23.144039
H	17.994739	36.589381	23.684426
H	18.231418	38.289189	24.102842
H	9.250892	33.987210	22.377221
H	13.768490	34.507014	25.849530
H	13.065222	35.468496	27.158891
H	14.334431	34.301235	27.512725
H	10.471882	34.011172	24.336316
H	12.002108	34.905086	24.269133
H	12.014984	33.147048	24.276263
H	8.697986	33.775424	18.696402
H	8.267706	35.189106	19.663608
H	7.929995	33.569159	20.279880
H	15.682442	38.873979	29.458573
H	15.806069	40.150440	28.240724
H	17.264750	39.341322	28.815466
H	19.781277	36.735766	17.763607
H	19.581724	38.901086	16.640110
H	21.648127	33.048726	18.804299
H	16.137266	39.668979	19.206298
H	16.655167	34.671342	15.549513
H	17.190296	35.370642	17.086217
H	18.267243	34.314548	16.189319
H	21.479730	38.317859	23.810962
H	15.162098	30.171524	18.981023
H	15.130972	32.939027	15.711979
H	20.555863	34.542359	25.609805
H	19.890684	32.710413	24.345126
H	18.804000	33.138923	23.013720
H	20.450699	32.637079	22.666273
H	19.938112	37.892899	20.808979
H	21.369735	38.649798	21.522279
H	21.531027	37.239645	20.461465
H	17.068103	31.924119	21.167568
H	16.775317	30.259776	20.645629
H	18.322329	31.050862	20.294820
H	22.476365	33.708727	21.855687
H	23.081587	33.801989	20.192951
H	22.742244	35.280543	21.121673
H	18.800911	40.626115	15.452782
H	18.411877	42.257558	16.004119
H	19.686754	41.337784	16.823899
H	19.136333	32.188279	16.696028

H	20.735108	31.778482	17.359192
H	19.268094	31.009028	17.988839
H	13.850629	29.682986	16.869717
H	12.870511	31.127072	17.134132
H	13.736631	30.921492	15.607740
H	16.820559	41.952081	18.773399
H	16.638381	42.550227	17.108199
H	15.666426	41.180385	17.665338
H	21.865187	36.323946	26.873217
H	22.032924	37.937919	26.161102
H	20.460712	37.387067	26.753457
H	15.948612	37.140820	19.166730

133
TS3

C	4.216534	0.998071	1.348752
C	3.683752	-0.268740	1.035599
C	3.470994	-1.214896	2.056547
C	3.813800	-0.881125	3.370279
C	4.353491	0.361486	3.704946
C	4.549245	1.283256	2.674436
N	3.357071	-0.604741	-0.315972
C	4.329770	-1.139328	-1.059000
C	4.163839	-1.651355	-2.361745
C	2.990287	-1.992089	-3.063767
C	3.202108	-2.815313	-4.320019
C	2.887053	-2.569524	1.750474
C	4.682772	0.711254	5.132845
C	4.423339	2.039104	0.280900
N	1.749335	-1.678054	-2.682353
Mg	1.519137	-0.197111	-1.221130
O	-0.222706	0.413931	-0.478038
Mg	-1.402389	0.302164	1.164223
N	-2.650147	-1.351506	1.268864
C	-3.885670	-1.434976	0.560683
C	-5.088343	-1.101855	1.215768
C	-6.280825	-1.121633	0.490722
C	-6.320297	-1.450225	-0.866212
C	-5.113779	-1.756699	-1.495383
C	-3.896187	-1.753652	-0.809112
C	-5.090603	-0.700706	2.666052
C	-7.625334	-1.485979	-1.618823
C	-2.619117	-2.096401	-1.522170
C	0.682014	-2.316108	-3.388408
C	0.272767	-3.605577	-2.987476
C	-0.740334	-4.249164	-3.698616
C	-1.375584	-3.649745	-4.789622
C	-0.967654	-2.366527	-5.152672
C	0.047736	-1.684116	-4.473342
C	0.922801	-4.284447	-1.810686
C	0.457025	-0.308040	-4.928254
C	-2.477667	-4.361478	-5.531085
N	1.494706	1.711800	-2.117403
C	0.255236	2.249281	-2.026907
C	-0.063324	3.473971	-2.603669

C	0.916003	4.203593	-3.306900
C	2.199460	3.607859	-3.417901
C	2.424997	2.390804	-2.811011
C	-0.786493	1.454770	-1.281104
C	-1.811505	2.463814	-0.565395
O	-2.146140	2.033850	0.573097
N	0.640646	5.422484	-3.852756
C	1.662266	6.140099	-4.586889
N	-0.845167	0.072566	3.145622
C	-0.691655	1.137840	4.073698
C	0.213171	2.185258	3.786790
C	0.329252	3.254491	4.672034
C	-0.439294	3.343754	5.836411
C	-1.352447	2.322581	6.081951
C	-1.502805	1.225368	5.225860
C	1.043021	2.156278	2.534899
C	-0.286710	4.508534	6.779854
C	-2.558442	0.201313	5.555548
C	-0.695016	5.979455	-3.734512
C	-0.605053	-1.186214	3.526872
C	-1.114533	-2.323191	2.863918
C	-2.203952	-2.393376	1.960488
C	-2.903000	-3.731891	1.848301
C	0.235583	-1.462977	4.757019
C	5.725452	-1.277901	-0.482336
H	-0.781898	-3.275917	3.263825
H	-3.862409	-3.702088	2.379566
H	-2.299917	-4.529942	2.288884
H	-3.127387	-3.984347	0.807344
H	-1.987586	2.380024	6.965593
H	0.972033	-0.672016	4.921117
H	0.756824	-2.418605	4.645481
H	-0.385197	-1.536427	5.657128
H	-5.112488	-2.003671	-2.556348
H	-1.838679	-1.348878	-1.341289
H	-2.773987	-2.172704	-2.601750
H	-2.208724	-3.056039	-1.183541
H	1.039523	4.047111	4.439075
H	0.428391	2.335610	1.642727
H	1.549506	1.195252	2.403646
H	1.808363	2.938109	2.557583
H	-7.206305	-0.859558	1.002136
H	-2.853046	-0.377503	4.677714
H	-3.452810	0.691714	5.956445
H	-2.218136	-0.509360	6.321489
H	-6.105585	-0.467115	3.003538
H	-4.465550	0.184454	2.832457
H	-4.695257	-1.491973	3.314769
H	-8.306948	-0.698310	-1.278366
H	-8.143215	-2.444725	-1.478654
H	-7.470527	-1.353961	-2.695115
H	0.693987	4.500545	7.273873
H	-1.050804	4.489385	7.563912
H	-0.372561	5.466687	6.252769
H	3.402803	1.919743	-2.879252

H	3.006107	4.080866	-3.964944
H	5.082255	-1.974995	-2.837978
H	-1.069110	3.840040	-2.438770
H	0.028171	-0.082072	-5.910053
H	0.113281	0.467357	-4.233484
H	1.545121	-0.203083	-4.997650
H	4.969585	2.260627	2.907935
H	-1.042589	-5.248857	-3.388758
H	-1.444470	-1.877957	-6.001404
H	3.659346	-1.621517	4.153691
H	3.018784	-3.248469	2.599317
H	1.808561	-2.504232	1.552500
H	3.346182	-3.030260	0.869739
H	3.470107	2.343945	-0.166120
H	4.899091	2.932702	0.697613
H	5.052984	1.674408	-0.539433
H	0.823108	-3.682950	-0.899235
H	0.465725	-5.261479	-1.623500
H	1.998134	-4.439741	-1.961589
H	5.725464	-2.006540	0.337307
H	6.431232	-1.621755	-1.241785
H	6.091398	-0.337324	-0.059590
H	1.990967	5.589269	-5.479719
H	1.260734	7.100359	-4.914262
H	2.543327	6.339852	-3.962259
H	2.656026	-2.393077	-5.169908
H	4.262455	-2.873476	-4.576213
H	2.826585	-3.836375	-4.186476
H	-2.217066	-5.407443	-5.730984
H	-3.410459	-4.367648	-4.951865
H	-2.690978	-3.879066	-6.490670
H	-0.987859	6.097068	-2.683755
H	-0.714914	6.963043	-4.206735
H	-1.443883	5.345715	-4.228040
H	5.573535	1.346267	5.196552
H	3.857234	1.260582	5.604418
H	4.864293	-0.185866	5.734373
H	-1.460513	1.003722	-2.035514

133
Product TS3

C	16.639209	37.501777	25.160072
C	15.785605	36.391962	25.356502
C	15.029571	36.306684	26.545059
C	15.181192	37.298405	27.521302
C	16.046757	38.376722	27.363600
C	16.760723	38.459299	26.164735
N	15.630573	35.439891	24.310880
C	15.976763	34.167161	24.505819
C	15.554494	33.098791	23.678224
C	14.451537	33.056070	22.791126
C	13.935308	31.681839	22.417206
C	14.028905	35.207443	26.796177
C	16.212759	39.417501	28.440225
C	17.399614	37.668441	23.874916

N	13.854584	34.140623	22.302039
Mg	15.004164	35.862301	22.377290
O	16.479829	36.038217	20.899410
Mg	18.119213	35.180979	20.140788
N	19.963138	34.865422	21.049596
C	20.318916	35.364631	22.341656
C	20.219994	34.508756	23.457391
C	20.558191	34.999503	24.720449
C	20.988207	36.313822	24.915471
C	21.084335	37.137789	23.793817
C	20.752563	36.693373	22.510814
C	19.760833	33.083256	23.296089
C	21.305856	36.831421	26.294002
C	20.865560	37.633816	21.340492
C	12.575714	34.023182	21.684002
C	11.422083	33.891913	22.485564
C	10.171641	33.815734	21.869070
C	10.021139	33.883277	20.483009
C	11.173205	34.053355	19.714992
C	12.445525	34.131952	20.286969
C	11.518081	33.892444	23.987902
C	13.649777	34.346933	19.414305
C	8.663541	33.766611	19.839702
O	14.335339	37.555227	21.708552
C	15.061843	38.068279	20.744343
C	16.150137	37.227443	20.299003
C	17.156507	37.793233	19.349505
N	18.173387	36.967663	19.011425
C	19.085681	37.423472	18.131963
C	19.034780	38.668596	17.547826
C	17.972902	39.549709	17.890782
C	17.043974	39.082095	18.838034
N	17.867392	40.793574	17.341421
C	18.885221	41.278569	16.431620
N	18.262436	33.505390	18.919356
C	17.137880	32.845538	18.332079
C	16.552778	33.344058	17.152459
C	15.460321	32.667942	16.601762
C	14.922259	31.521727	17.187657
C	15.514209	31.053762	18.363071
C	16.607987	31.694837	18.949943
C	17.095067	34.574661	16.475212
C	13.723998	30.828671	16.593006
C	17.205698	31.156871	20.223746
C	16.795363	41.676964	17.764038
C	19.478450	33.078982	18.570622
C	20.685143	33.506817	19.162993
C	20.908143	34.228689	20.353674
C	22.334753	34.221143	20.866407
C	19.618709	32.025963	17.489116
C	16.854016	33.787575	25.680966
H	15.989876	32.134119	23.918385
H	12.961351	31.490302	22.881713
H	14.624988	30.899275	22.743330
H	13.789564	31.603439	21.334391

H	14.586815	37.222740	28.431572
H	17.588528	34.569696	25.893697
H	17.377919	32.849908	25.476383
H	16.257640	33.636293	26.587752
H	11.084804	34.135090	18.632300
H	14.103140	35.328970	19.600327
H	13.376041	34.309905	18.355794
H	14.427816	33.595439	19.583750
H	17.429139	39.303432	25.999146
H	16.726603	37.951696	23.054842
H	17.918450	36.750499	23.582813
H	18.147730	38.461915	23.965636
H	9.286593	33.715474	22.496316
H	13.789942	34.653784	25.886050
H	13.096632	35.624375	27.194841
H	14.393998	34.485548	27.539847
H	10.523351	33.822707	24.440001
H	11.989658	34.815448	24.344703
H	12.119985	33.062517	24.377688
H	8.615974	34.326197	18.899025
H	7.874218	34.147086	20.497391
H	8.417358	32.720943	19.608838
H	17.151321	39.277073	28.993569
H	15.395198	39.374753	29.167694
H	16.235557	40.429862	28.020072
H	19.896983	36.739248	17.895432
H	19.812820	38.955253	16.850821
H	21.579879	33.091497	18.713255
H	16.212076	39.672338	19.202006
H	16.566083	34.765984	15.535971
H	16.986801	35.463457	17.106786
H	18.163884	34.483337	16.247531
H	21.427612	38.164169	23.916548
H	15.116733	30.158564	18.839805
H	15.019183	33.052083	15.683024
H	20.486342	34.330542	25.576882
H	19.846702	32.540334	24.242720
H	18.712475	33.034336	22.975859
H	20.342916	32.540994	22.542270
H	19.878226	37.925077	20.963648
H	21.392810	38.549047	21.628886
H	21.402636	37.183793	20.498061
H	17.054739	31.850818	21.060614
H	16.740432	30.204012	20.495382
H	18.286470	30.995215	20.140993
H	22.411669	33.633708	21.788818
H	23.015047	33.787511	20.129892
H	22.677245	35.231343	21.113118
H	18.977552	40.631132	15.549693
H	18.608691	42.275240	16.084245
H	19.871339	41.348513	16.912982
H	19.062027	32.298096	16.586633
H	20.667083	31.871281	17.224194
H	19.207752	31.068242	17.829631
H	13.787916	29.741305	16.712555

H	12.795740	31.154027	17.081006
H	13.624750	31.045344	15.524061
H	16.875262	41.939071	18.827844
H	16.838753	42.596993	17.178806
H	15.814411	41.215296	17.599587
H	20.401524	37.208303	26.789677
H	21.723151	36.045300	26.932993
H	22.026608	37.655465	26.257827
H	15.057350	37.415016	19.548396

133
TS4

C	0.069990	1.981665	3.914637
C	-0.823440	0.895684	4.062254
C	-1.624228	0.814214	5.221220
C	-1.484752	1.789965	6.215339
C	-0.587347	2.847716	6.103827
C	0.176242	2.923999	4.935422
N	-0.970589	-0.033001	2.995207
C	-0.662608	-1.317758	3.171171
C	-1.088604	-2.354841	2.305894
C	-2.163867	-2.347395	1.384985
C	-2.691066	-3.697986	0.946173
C	-2.656335	-0.265904	5.422915
C	-0.437190	3.872405	7.198100
C	0.900388	2.131926	2.671613
N	-2.723824	-1.234956	0.911992
Mg	-1.518540	0.437170	1.051934
O	-0.062252	0.709629	-0.377355
Mg	1.546562	-0.139200	-1.184319
N	3.392843	-0.490262	-0.279071
C	3.760522	0.022750	1.002965
C	3.660907	-0.814804	2.132643
C	4.016137	-0.308781	3.385009
C	4.465026	1.002841	3.555255
C	4.557394	1.809583	2.420685
C	4.207556	1.349826	1.147792
C	3.185019	-2.237371	1.996227
C	4.806166	1.535152	4.922736
C	4.321851	2.265363	-0.041792
C	-3.987767	-1.301541	0.258515
C	-5.163242	-1.467274	1.022740
C	-6.398258	-1.493755	0.372749
C	-6.513507	-1.342703	-1.010519
C	-5.341457	-1.137946	-1.738037
C	-4.082200	-1.106192	-1.132191
C	-5.108575	-1.552068	2.525100
C	-2.855643	-0.854107	-1.963474
C	-7.856185	-1.410500	-1.691049
O	-2.186148	2.192300	0.509262
C	-1.431438	2.639431	-0.446994
C	-0.345629	1.946572	-0.940476
C	0.511359	2.437315	-1.991449
N	1.555078	1.619919	-2.325925
C	2.403293	2.019444	-3.291693

C	2.295337	3.201367	-3.982109
C	1.211851	4.073814	-3.668835
C	0.333761	3.666330	-2.660239
N	1.045749	5.259224	-4.337209
C	2.023437	5.688435	-5.315759
N	1.672971	-1.836283	-2.394584
C	0.540761	-2.485342	-2.978457
C	-0.038448	-1.990278	-4.162648
C	-1.138475	-2.658565	-4.707981
C	-1.691067	-3.792802	-4.112667
C	-1.103484	-4.259288	-2.934509
C	-0.001397	-3.626897	-2.353637
C	0.513460	-0.768927	-4.848220
C	-2.898213	-4.475593	-4.701328
C	0.595281	-4.166374	-1.080062
C	-0.032558	6.149530	-3.962790
C	2.881565	-2.283433	-2.743052
C	4.095347	-1.873282	-2.153822
C	4.328969	-1.144407	-0.969939
C	5.756439	-1.166434	-0.459089
C	3.004715	-3.341234	-3.822532
C	0.172192	-1.745396	4.360590
H	-0.679479	-3.334938	2.528791
H	-3.684115	-3.888830	1.368070
H	-2.026697	-4.503649	1.268322
H	-2.797132	-3.734982	-0.143434
H	-2.113867	1.717670	7.102260
H	0.917993	-0.985651	4.612603
H	0.680052	-2.690244	4.148495
H	-0.452874	-1.902762	5.246933
H	-5.401881	-0.994062	-2.816192
H	-2.410179	0.123683	-1.738999
H	-3.100992	-0.863150	-3.029659
H	-2.079519	-1.607609	-1.795566
H	0.875519	3.749745	4.808656
H	0.287517	2.419318	1.807320
H	1.421197	1.205632	2.410914
H	1.655986	2.912335	2.802085
H	-7.299516	-1.622357	0.971177
H	-2.876829	-0.801173	4.497316
H	-3.592019	0.164803	5.798328
H	-2.330107	-1.006304	6.166444
H	-6.115910	-1.644038	2.944186
H	-4.646498	-0.651773	2.946486
H	-4.519317	-2.403699	2.885715
H	-7.851831	-0.867184	-2.642265
H	-8.646605	-0.983443	-1.063475
H	-8.142899	-2.448324	-1.909985
H	0.488802	3.718177	7.768513
H	-1.270042	3.825340	7.907734
H	-0.398946	4.890518	6.792880
H	3.217203	1.331913	-3.512351
H	3.026361	3.443117	-4.743009
H	4.983426	-2.304539	-2.601786
H	-0.499043	4.296083	-2.379755

H	0.012977	-0.603169	-5.807856
H	0.374900	0.130688	-4.238311
H	1.589827	-0.852870	-5.038596
H	4.912468	2.834113	2.524568
H	-1.509650	-5.147480	-2.451890
H	-1.574294	-2.277038	-5.630451
H	3.943709	-0.963238	4.252688
H	3.254668	-2.761630	2.954656
H	2.140051	-2.281295	1.664946
H	3.768332	-2.801767	1.259620
H	3.341716	2.472736	-0.485752
H	4.769022	3.222170	0.246804
H	4.938037	1.831591	-0.838369
H	0.456528	-3.467343	-0.245406
H	0.120151	-5.112777	-0.802737
H	1.673706	-4.340875	-1.167248
H	5.826873	-1.742961	0.470678
H	6.428910	-1.619191	-1.191301
H	6.114599	-0.158351	-0.226420
H	2.112224	4.969431	-6.140869
H	1.707186	6.642312	-5.741744
H	3.020686	5.829257	-4.874029
H	2.465038	-3.054305	-4.730893
H	4.051268	-3.523363	-4.077092
H	2.564700	-4.287668	-3.487102
H	-2.848291	-5.562691	-4.572492
H	-3.822315	-4.134110	-4.216384
H	-2.994505	-4.267017	-5.772221
H	0.060522	6.497116	-2.923341
H	-0.021623	7.024116	-4.615622
H	-1.009527	5.660440	-4.072822
H	3.906812	1.890362	5.442950
H	5.261310	0.763079	5.553257
H	5.504293	2.377057	4.863380
H	-1.690169	3.618810	-0.861904

135

Adduct TS5

C	-4.746595	1.255665	-0.210687
C	-4.156105	-0.014642	-0.353818
C	-3.953168	-0.560337	-1.638220
C	-4.367509	0.165242	-2.755964
C	-4.977834	1.417080	-2.641111
C	-5.147807	1.944980	-1.360734
N	-3.734224	-0.746609	0.796606
C	-4.617255	-1.577162	1.366357
C	-5.999804	-1.713572	0.759647
C	-3.321185	-1.916712	-1.799532
C	-5.459954	2.153457	-3.864892
C	-4.968915	1.860145	1.151213
Mg	-1.818984	-0.515875	1.530247
O	-1.520395	2.045101	2.120391
C	-0.449443	2.491117	2.415674
O	-0.046474	-0.407232	0.702166
C	0.750708	0.411407	1.508619
C	1.563418	0.148844	2.508238

O	2.337711	0.214538	3.404543
Mg	1.494211	0.289564	-0.559913
N	0.343979	1.550812	-1.910943
C	-0.782020	2.211552	-1.587726
C	-1.380886	3.160063	-2.393585
C	-0.810313	3.475319	-3.649094
C	0.363606	2.763301	-3.991362
C	0.887549	1.839436	-3.108436
N	-1.356583	4.410220	-4.478459
C	-2.485724	5.203291	-4.029439
N	2.157353	-1.283245	-1.792076
C	3.188061	-1.141166	-2.628112
C	3.549884	-2.272855	-3.571143
C	1.497593	-2.548079	-1.762921
C	0.410608	-2.809313	-2.621748
C	-0.220721	-4.053365	-2.558238
C	0.185240	-5.047886	-1.664421
C	1.254462	-4.760048	-0.816414
C	1.917403	-3.529195	-0.845661
C	-0.063187	-1.770040	-3.603588
C	3.062005	-3.260540	0.094530
C	-0.505026	-6.387420	-1.631125
C	4.029047	-0.011739	-2.684768
C	4.195468	1.027725	-1.739993
N	3.378452	1.253143	-0.717316
C	3.826389	2.152627	0.301905
C	4.674558	1.664394	1.317705
C	5.096120	2.531380	2.325957
C	4.701621	3.869723	2.367442
C	3.865366	4.329622	1.351502
C	3.413899	3.497238	0.323039
C	5.112480	0.224279	1.333443
C	5.139870	4.774330	3.489807
C	2.500583	4.056796	-0.734387
C	5.429801	1.890109	-1.925545
N	-2.125906	-1.717649	3.167839
C	-3.242841	-2.416831	3.352059
C	-3.342878	-3.357582	4.534742
C	-1.067652	-1.852188	4.121074
C	-1.025012	-1.005335	5.244893
C	0.041748	-1.129169	6.137508
C	1.065983	-2.057357	5.945559
C	0.996109	-2.885080	4.824117
C	-0.047024	-2.795801	3.900306
C	-2.105513	0.016102	5.489128
C	2.236428	-2.128537	6.890371
C	-0.069757	-3.686128	2.686198
C	-0.689725	4.753089	-5.719779
C	-4.375265	-2.352315	2.512553
H	-5.200080	-2.987234	2.815043
H	-6.529011	-0.753929	0.771924
H	-6.599350	-2.443604	1.307656
H	-5.946169	-2.024397	-0.289521
H	0.076793	-0.469676	7.003398
H	-2.542504	-4.105259	4.504523

H	-4.304514	-3.875408	4.549428
H	-3.223849	-2.815680	5.479198
H	-4.219751	-0.267442	-3.744736
H	-2.294973	-1.941694	-1.413917
H	-3.284522	-2.204400	-2.854645
H	-3.872877	-2.693630	-1.257201
H	1.785187	-3.615749	4.651525
H	0.058736	-3.108275	1.762253
H	-1.015234	-4.232829	2.587543
H	0.740554	-4.420726	2.730350
H	-5.624946	2.917629	-1.244541
H	-2.186466	0.726189	4.658278
H	-1.896534	0.589325	6.397957
H	-3.094701	-0.445010	5.603390
H	-5.499931	2.814234	1.069618
H	-4.022032	2.043930	1.670799
H	-5.558618	1.201974	1.800185
H	-5.796985	3.165944	-3.617205
H	-6.305691	1.635233	-4.335370
H	-4.673163	2.233353	-4.625159
H	2.647008	-3.142740	6.948868
H	1.957234	-1.817034	7.902923
H	3.044808	-1.466136	6.554237
H	1.802389	1.306126	-3.358303
H	0.878407	2.934128	-4.929223
H	4.778967	-0.039208	-3.468044
H	-2.287344	3.637257	-2.043463
H	-0.873742	-2.164441	-4.224866
H	-0.433141	-0.874593	-3.091689
H	0.739422	-1.437053	-4.272424
H	3.546558	5.371416	1.355724
H	1.589696	-5.516904	-0.108615
H	-1.053202	-4.251685	-3.232603
H	5.745084	2.142750	3.109451
H	5.780637	0.033273	2.179027
H	4.250893	-0.444511	1.433663
H	5.636093	-0.066431	0.414699
H	1.466023	3.728925	-0.576752
H	2.505613	5.151923	-0.709241
H	2.785301	3.736027	-1.742149
H	2.821211	-2.449658	0.793003
H	3.298648	-4.150953	0.685946
H	3.970226	-2.953519	-0.436760
H	6.179276	1.666176	-1.157464
H	5.885841	1.718959	-2.904096
H	5.190381	2.953636	-1.824606
H	-0.580867	3.874266	-6.367340
H	-1.290011	5.488667	-6.257353
H	0.307559	5.183463	-5.549178
H	2.682533	-2.636599	-4.130747
H	4.322311	-1.963371	-4.279256
H	3.933575	-3.129305	-3.003371
H	-0.337339	-6.948909	-2.559559
H	-0.140377	-7.003558	-0.802843
H	-1.590156	-6.278617	-1.513198

H	-2.238705	5.805923	-3.143937
H	-2.787166	5.881107	-4.829590
H	-3.345395	4.567778	-3.786501
H	5.080426	5.829712	3.201896
H	4.505497	4.640721	4.376216
H	6.171385	4.565721	3.796327
H	-1.214401	1.971660	-0.621940

135

TS5

C	0.410608	-2.809313	-2.621748
C	1.497593	-2.548079	-1.762921
C	1.917403	-3.529195	-0.845661
C	1.254462	-4.760048	-0.816414
C	0.185240	-5.047886	-1.664421
C	-0.220721	-4.053365	-2.558238
N	2.157353	-1.283245	-1.792076
C	3.188061	-1.141166	-2.628112
C	4.029047	-0.011739	-2.684768
C	4.195468	1.027725	-1.739993
C	5.429801	1.890109	-1.925545
C	3.062005	-3.260540	0.094530
C	-0.505026	-6.387420	-1.631125
C	-0.063187	-1.770040	-3.603588
N	3.378452	1.253143	-0.717316
Mg	1.494211	0.289564	-0.559913
O	-0.046474	-0.407232	0.702166
Mg	-1.818984	-0.515875	1.530247
N	-2.125906	-1.717649	3.167839
C	-1.067652	-1.852188	4.121074
C	-1.025013	-1.005335	5.244893
C	0.041747	-1.129169	6.137508
C	1.065982	-2.057356	5.945559
C	0.996109	-2.885079	4.824117
C	-0.047024	-2.795801	3.900306
C	-2.105514	0.016102	5.489128
C	2.236428	-2.128536	6.890371
C	-0.069757	-3.686128	2.686198
C	3.826390	2.152627	0.301905
C	4.674559	1.664394	1.317705
C	5.096121	2.531381	2.325957
C	4.701622	3.869724	2.367442
C	3.865367	4.329623	1.351502
C	3.413900	3.497239	0.323039
C	5.112481	0.224279	1.333443
C	2.500584	4.056797	-0.734387
C	5.139871	4.774331	3.489807
N	0.343979	1.550812	-1.910942
C	-0.782020	2.211552	-1.587725
C	-1.380886	3.160063	-2.393584
C	-0.810313	3.475319	-3.649094
C	0.363606	2.763301	-3.991362
C	0.887549	1.839436	-3.108436
N	-1.356583	4.410220	-4.478459
C	-0.689725	4.753089	-5.719779
C	-2.485724	5.203291	-4.029439

C	0.750708	0.411407	1.508619
C	1.563418	0.148844	2.508239
O	2.337711	0.214538	3.404544
O	-1.579952	1.534283	2.002680
C	-0.509000	1.980299	2.297963
N	-3.734224	-0.746609	0.796606
C	-4.156105	-0.014642	-0.353818
C	-3.953168	-0.560337	-1.638220
C	-4.367509	0.165242	-2.755964
C	-4.977834	1.417080	-2.641111
C	-5.147807	1.944980	-1.360734
C	-4.746595	1.255665	-0.210687
C	-3.321185	-1.916712	-1.799532
C	-5.459954	2.153457	-3.864892
C	-4.968915	1.860145	1.151213
C	-4.617255	-1.577162	1.366357
C	-4.375265	-2.352315	2.512553
C	-3.242841	-2.416831	3.352059
C	-3.342879	-3.357582	4.534742
C	-5.999804	-1.713572	0.759647
C	3.549884	-2.272855	-3.571142
H	-5.200080	-2.987234	2.815043
H	-6.529011	-0.753929	0.771924
H	-6.599350	-2.443604	1.307656
H	-5.946169	-2.024397	-0.289521
H	0.076792	-0.469675	7.003398
H	-2.542505	-4.105259	4.504523
H	-4.304515	-3.875408	4.549428
H	-3.223850	-2.815680	5.479198
H	-4.219751	-0.267442	-3.744736
H	-2.294973	-1.941694	-1.413917
H	-3.284522	-2.204400	-2.854645
H	-3.872877	-2.693630	-1.257201
H	1.785187	-3.615748	4.651525
H	0.058736	-3.108275	1.762253
H	-1.015234	-4.232829	2.587543
H	0.740554	-4.420726	2.730350
H	-5.624946	2.917629	-1.244541
H	-2.186467	0.726189	4.658278
H	-1.896535	0.589325	6.397957
H	-3.094702	-0.445010	5.603390
H	-5.499931	2.814234	1.069618
H	-4.022032	2.043930	1.670799
H	-5.558618	1.201974	1.800185
H	-5.796985	3.165944	-3.617205
H	-6.305691	1.635233	-4.335370
H	-4.673163	2.233353	-4.625159
H	2.647008	-3.142739	6.948869
H	1.957234	-1.817033	7.902923
H	3.044807	-1.466135	6.554237
H	1.802389	1.306126	-3.358303
H	0.878407	2.934128	-4.929223
H	4.778967	-0.039208	-3.468044
H	-2.287344	3.637257	-2.043463
H	-0.873742	-2.164441	-4.224865

H	-0.433141	-0.874593	-3.091688
H	0.739422	-1.437053	-4.272424
H	3.546559	5.371417	1.355724
H	1.589696	-5.516904	-0.108615
H	-1.053202	-4.251685	-3.232603
H	5.745085	2.142751	3.109451
H	5.780638	0.033274	2.179028
H	4.250894	-0.444511	1.433664
H	5.636094	-0.066431	0.414699
H	1.466023	3.728926	-0.576751
H	2.505614	5.151924	-0.709241
H	2.785301	3.736028	-1.742149
H	2.821211	-2.449658	0.793003
H	3.298648	-4.150953	0.685946
H	3.970226	-2.953519	-0.436760
H	6.179276	1.666176	-1.157464
H	5.885841	1.718959	-2.904096
H	5.190381	2.953636	-1.824606
H	-0.580867	3.874266	-6.367340
H	-1.290011	5.488667	-6.257353
H	0.307559	5.183463	-5.549178
H	2.682534	-2.636599	-4.130747
H	4.322311	-1.963371	-4.279255
H	3.933575	-3.129305	-3.003370
H	-0.337339	-6.948909	-2.559559
H	-0.140377	-7.003558	-0.802843
H	-1.590156	-6.278617	-1.513198
H	-2.238705	5.805923	-3.143937
H	-2.787166	5.881107	-4.829590
H	-3.345395	4.567778	-3.786501
H	5.080427	5.829713	3.201896
H	4.505498	4.640722	4.376216
H	6.171386	4.565722	3.796327
H	-1.214401	1.971660	-0.621939

135

Product TS5

C	0.207237	-2.497812	-3.520227
C	1.098204	-2.497212	-2.429691
C	1.205434	-3.642036	-1.611915
C	0.418811	-4.759565	-1.896150
C	-0.469542	-4.785489	-2.973835
C	-0.551221	-3.645636	-3.773647
N	1.888804	-1.347041	-2.120478
C	3.128000	-1.290835	-2.624698
C	4.105202	-0.339435	-2.275495
C	4.127973	0.591717	-1.209465
C	5.475601	1.204240	-0.890141
C	2.176637	-3.673067	-0.462098
C	-1.320321	-5.998134	-3.249495
C	0.066333	-1.299098	-4.420975
N	3.068338	0.919278	-0.479262
Mg	1.177643	0.249988	-0.989889
O	-0.159915	0.279894	0.491295
Mg	-1.657883	-0.059902	2.137770
N	-1.507945	-1.465011	3.630871

C	-0.338043	-1.521993	4.449288
C	-0.157764	-0.591713	5.493265
C	1.016861	-0.651938	6.247777
C	2.013174	-1.599687	6.004426
C	1.813837	-2.500860	4.956964
C	0.657725	-2.478358	4.172605
C	-1.208207	0.441786	5.803470
C	3.249551	-1.665333	6.864241
C	0.478507	-3.468410	3.052418
C	3.253631	1.742754	0.678875
C	3.459554	1.116741	1.924664
C	3.584390	1.906251	3.068643
C	3.509182	3.299828	3.014718
C	3.312974	3.893309	1.768564
C	3.174819	3.144945	0.595624
C	3.534905	-0.383234	2.028975
C	2.904611	3.856836	-0.703441
C	3.603346	4.132835	4.266692
N	0.362398	1.616917	-2.427266
C	-0.881425	2.133069	-2.411265
C	-1.333022	3.079296	-3.307506
C	-0.465114	3.571967	-4.308848
C	0.836489	3.013934	-4.331033
C	1.189296	2.068373	-3.390890
N	-0.857893	4.525947	-5.197871
C	0.085261	5.046059	-6.169085
C	-2.171989	5.129898	-5.074599
C	-0.374313	1.571172	0.828851
C	-0.499513	3.007406	0.738716
O	-0.304875	4.062336	0.154054
O	-1.839794	1.870899	2.806088
C	-1.087361	2.209700	1.806509
N	-3.426796	-0.778808	1.374684
C	-3.935482	-0.222798	0.163882
C	-3.650293	-0.860306	-1.061233
C	-4.108344	-0.283817	-2.247194
C	-4.836666	0.909745	-2.255893
C	-5.092471	1.526802	-1.031134
C	-4.657563	0.986126	0.184498
C	-2.867031	-2.144723	-1.087975
C	-5.344552	1.494629	-3.549376
C	-4.972514	1.682512	1.482370
C	-4.158309	-1.672049	2.039243
C	-3.755814	-2.313862	3.227986
C	-2.550851	-2.234194	3.953754
C	-2.472059	-3.095841	5.198139
C	-5.528178	-2.044785	1.510533
C	3.566359	-2.339791	-3.628568
H	-4.490881	-2.992022	3.646001
H	-6.172290	-1.161039	1.439706
H	-6.016108	-2.779562	2.154745
H	-5.459202	-2.460448	0.499169
H	1.152012	0.065204	7.056384
H	-1.642086	-3.808242	5.138642
H	-3.397658	-3.655125	5.350922

H	-2.287488	-2.478781	6.084758
H	-3.891403	-0.785504	-3.189715
H	-1.879134	-2.022065	-0.629020
H	-2.715746	-2.493718	-2.113028
H	-3.368591	-2.944313	-0.529132
H	2.578954	-3.245552	4.740397
H	0.251363	-2.968650	2.103817
H	-0.354382	-4.157891	3.240413
H	1.383305	-4.068837	2.915244
H	-5.657130	2.457972	-1.011860
H	-1.352982	1.139847	4.969955
H	-0.927198	1.022991	6.687932
H	-2.184788	-0.017689	6.000354
H	-5.542184	2.599467	1.299461
H	-4.062487	1.948629	2.033400
H	-5.568359	1.046272	2.149063
H	-5.753112	2.499861	-3.400250
H	-6.143109	0.877572	-3.981815
H	-4.549006	1.560034	-4.302080
H	4.089665	-2.118477	6.326697
H	3.077036	-2.269184	7.765494
H	3.560593	-0.669274	7.198447
H	2.190549	1.642860	-3.398895
H	1.574706	3.323175	-5.060985
H	5.044987	-0.440673	-2.806778
H	-2.345787	3.445509	-3.199087
H	-0.471957	-1.565261	-5.336838
H	-0.490341	-0.491209	-3.932043
H	1.037131	-0.879995	-4.706152
H	3.248700	4.978171	1.699912
H	0.511447	-5.639190	-1.260262
H	-1.225126	-3.645731	-4.629624
H	3.736285	1.414793	4.028411
H	3.753727	-0.690925	3.055434
H	2.582247	-0.848461	1.746917
H	4.301523	-0.808750	1.370149
H	1.821745	3.974551	-0.841104
H	3.342862	4.861136	-0.689724
H	3.306872	3.320166	-1.567745
H	2.014656	-2.839747	0.230013
H	2.082974	-4.606816	0.100861
H	3.216493	-3.588933	-0.802087
H	5.818421	0.874791	0.097897
H	6.227802	0.915758	-1.628182
H	5.416858	2.296630	-0.848973
H	0.460031	4.251724	-6.827356
H	-0.416029	5.786931	-6.793529
H	0.944885	5.531961	-5.686710
H	2.846977	-2.437481	-4.448299
H	4.546016	-2.098074	-4.047084
H	3.632364	-3.326706	-3.155682
H	-1.691873	-6.001368	-4.279679
H	-0.760242	-6.926709	-3.090658
H	-2.194683	-6.031284	-2.585945
H	-2.301354	5.627222	-4.104000

H	-2.297479	5.876922	-5.859649
H	-2.967437	4.382711	-5.190516
H	4.034802	5.118819	4.061788
H	2.610464	4.297610	4.705937
H	4.220321	3.644847	5.029443
H	-1.546772	1.765743	-1.636730

References

1. R. Savka and H. Plenio, *Eur. J. Inorg. Chem.*, 2014, 6246.
2. S. J. Bonyhady, D. Collis, N. Holzmann, A. J. Edwards, R. O. Piltz, G. Frenking, A. Stasch and C. Jones, *Nat. Commun.*, 2018, **9**, 3079.
3. S. J. Bonyhady, C. Jones, S. Nembenna, A. Stasch, A. J. Edwards and G. J. McIntyre, *Chem. Eur. J.*, 2010, **16**, 938.
4. R. Lalrempuia, C. E. Kefalidis, S. J. Bonhady, B. Schwarze, L. Maron, A. Stasch and C. Jones, *J. Am. Chem. Soc.*, 2015, **137**, 8944.
5. K. Yuvaraj, I. Douair, A. Paparo, L. Maron and C. Jones, *J. Am. Chem. Soc.*, 2019, **141**, 8764.
6. T. M. McPhillips, S. McPhillips, H. J. Chiu, A. E. Cohen, A. M. Deacon, P.J . Ellis, E. Garman, A. Gonzalez, N. K. Sauter, R. P. Phizackerley, S. M. Soltis and P. Kuhn, *J. Synchrotron Rad.*, 2002, **9**, 401.
7. W. J. Kabsch, *Appl. Cryst.*, 1993, **26**, 795.
8. G.M. Sheldrick, *SHELX-16*, University of Göttingen, 2016.
9. A. L. Spek, *Acta Cryst.*, 2015, **C71**, 9.
10. M. J. Frisch, G. W. Trucks, H. B. Schlegel, G. E. Scuseria, M. A. Robb, J. R. Cheeseman, G. Scalmani, V. Barone, B. Mennucci, G. A. Petersson, H. Nakatsuji, M. Caricato, X. Li, H. P. Hratchian, A. F. Izmaylov, J. Bloino, G. Zheng, J. L. Sonnenberg, M. Hada, M. Ehara, K. Toyota, R. Fukuda, J. Hasegawa, M. Ishida, T. Nakajima, Y. Honda, O. Kitao, H. Nakai, T. Vreven, J. A. Montgomery, Jr., J. E. Peralta, F. Ogliaro, M. Bearpark, J. J. Heyd, E. Brothers, K. N. Kudin, V. N. Staroverov, R. Kobayashi, J. Normand, K. Raghavachari, A. Rendell, J. C. Burant, S. S. Iyengar, J. Tomasi, M. Cossi, N. Rega, J. M. Millam, M. Klene, J. E. Knox, J. B. Cross, V. Bakken, C. Adamo, J. Jaramillo, R. Gomperts, R. E. Stratmann, O. Yazyev, A. J. Austin, R. Cammi, C. Pomelli, J. W. Ochterski, R. L. Martin, K. Morokuma, V. G. Zakrzewski, G. A. Voth, P. Salvador, J. J. Dannenberg, S. Dapprich, A. D.

Daniels, O. Farkas, J. B. Foresman, J. V. Ortiz, J. Cioslowski, D. J. Fox, GAUSSIAN 09, Revision A.02; Gaussian, Inc., Wallingford CT, 2009.

11. A. D. Becke, *J. Chem. Phys.*, 1993, **98**, 5648.
12. J. P. Perdew and Y. Wang, *Phys. Rev. B*, 1992, **45**, 13244.
13. (a) A. D. McLean and G. S. Chandler, *J. Chem. Phys.*, 1980, **72**, 5639. (b) W. J. Hehre, R. Ditchfield and J. A. Pople, *J. Chem. Phys.*, 1972, **56**, 2257.
14. (a) C. Gonzalez and H. B. Schlegel, *J. Chem. Phys.*, 1989, **90**, 2154. (b) C. Gonzalez and H. B. Schlegel, *J. Phys. Chem.*, 1990, **94**, 5523.
